# Association of Serum Mannose With Acute Respiratory Distress Syndrome Risk and Survival

**DOI:** 10.1001/jamanetworkopen.2020.34569

**Published:** 2021-01-27

**Authors:** Yongyue Wei, Hui Huang, Ruyang Zhang, Zhaozhong Zhu, Ying Zhu, Lijuan Lin, Xuesi Dong, Liangmin Wei, Xin Chen, Zhonghua Liu, Yang Zhao, Li Su, Feng Chen, David C. Christiani

**Affiliations:** 1 Department of Biostatistics, Nanjing Medical University School of Public Health, Nanjing, Jiangsu, China; 2 China International Cooperation Center for Environment and Human Health, Center for Global Health, Nanjing Medical University, Nanjing, Jiangsu, China; 3 Department of Environmental Health, Harvard T.H. Chan School of Public Health, Boston, Massachusetts; 4 Department of Statistics and Actuarial Science, The University of Hong Kong, Hong Kong, China; 5 Department of Medicine, Massachusetts General Hospital, Boston

## Abstract

**Importance:**

Acute respiratory distress syndrome (ARDS) confers high mortality risk among critically ill patients. Identification of biomarkers associated with ARDS risk may guide clinical diagnosis and prognosis.

**Objective:**

To systematically evaluate the association of blood metabolites with ARDS risk and survival.

**Design, Setting, and Participants:**

In this cohort study, data from the Molecular Epidemiology of ARDS (MEARDS) study, a prospective cohort of 403 patients with ARDS and 1227 non-ARDS controls, were analyzed. Patients were recruited in intensive care units (ICUs) at Massachusetts General Hospital and Beth Israel Deaconess Medical Center, both in Boston, Massachusetts, from January 1, 1998, to December 31, 2014. Data analysis was performed from December 9, 2018, to January 4, 2019.

**Main Outcomes and Measures:**

Participants were followed up daily for ARDS development defined by Berlin criteria, requiring fulfillment of chest radiograph and oxygenation criteria on the same calendar day during invasive ventilatory assistance. A 2-stage study design was used to explore novel metabolites associated with ARDS risk and survival.

**Results:**

Of the 1630 participants from MEARDS who were admitted to the ICU , 403 (24.7%) were diagnosed with ARDS (mean [SD] age, 63.0 [17.0] years; 251 [62.3%] male) and 1227 (75.3%) were at-risk but did not have ARDS (mean [SD] age, 62.3 [16.9] years; 753 [61.4%] male). Mendelian randomization suggested that genetically regulated serum mannose was associated with ARDS risk (odds ratio [OR], 0.64; 95% CI, 0.53-0.78; *P* = 7.46 × 10^−6^) in the discovery stage. In the functional validation stage incorporating 83 participants with ARDS and matched at-risk participants in the control group from the ICU, the protective association of mannose with ARDS risk was validated (OR, 0.67; 95% CI, 0.46-0.97; *P* = .03). Furthermore, serum mannose was associated with 28-day (OR, 0.25; 95% CI, 0.11-0.56; *P* = 6.95 × 10^−4^) and 60-day (OR, 0.36; 95% CI, 0.19-0.71; *P* = 3.12 × 10^−3^) mortality and 28-day (hazard ratio, 0.49; 95% CI, 0.32-0.74; *P* = 6.41 × 10^−4^) and 60-day (hazard ratio, 0.55; 95% CI, 0.37-0.80; *P* = 2.11 × 10^−3^) survival.

**Conclusions and Relevance:**

In this study, genetically regulated serum mannose appeared to be associated with ARDS risk and outcome, and increased serum mannose at admission was associated with reduced ARDS risk and better survival. These findings could inform prevention and clinical intervention in ARDS cases, which have increased with the expansion of the coronavirus disease 2019 pandemic.

## Key Points

**Question:**

Are serum metabolites associated with acute respiratory distress syndrome (ARDS) risk and survival among critically ill patients?

**Findings:**

This cohort study, a 2-sample mendelian randomization analysis of 1630 participants, found that genetically regulated high-level serum mannose is associated with reduced ARDS risk and improved ARDS survival.

**Meaning:**

This metabolome-wide association study identified mannose as a potential biomarker and therapeutic candidate for treating critically ill patients with ARDS.

## Teaser

This cohort study systematically evaluates the association of blood metabolites with acute respiratory distress syndrome risk and survival.

## Introduction

Acute respiratory distress syndrome (ARDS) management exerts a major health care burden worldwide,^1^ a burden undergoing rapid expansion because of the novel coronavirus disease 2019 (COVID-19) pandemic, which is affecting more than 25 million individuals worldwide^2^ and, when severe, causes pneumonia and a subtype of ARDS.^3^ ARDS is generally characterized by bilateral lung infiltrates with acute hypoxemic respiratory failure that is caused by intrapulmonary or extrapulmonary precipitants.^4^ The prevalence of ARDS in intensive care units (ICUs) is approximately 10%, but it carries a mortality rate of 40%.^5^ Treatment typically consists of life-sustaining ventilatory and nonventilatory strategies, and no effective pharmacotherapy is available.^1^ Because effective treatments remain elusive, emphasis has shifted from management strategies toward early identification and prevention of ARDS.^6,7^ Indeed, numerous predisposing factors (eg, sepsis, pneumonia, lung injury, and multiple transfusions) are well recognized as leading risk factors of ARDS onset. Furthermore, emerging trans-omics studies^8,9^ have identified biomarkers (eg, genes, transcripts, metabolites, and proteins) involved in the pathogenesis and progression of ARDS.

Metabolites, the low-molecular-mass endogenous and exogenous chemicals actively involved in biochemical processes in the body, are potential biomarkers for ARDS. As the intermediate or end products of metabolism, metabolites capture dynamic snapshots of physiologic status and environmental exposure.^10^ Metabolite changes are sensitive to pathophysiologic aberrations, providing an opportunity to determine susceptibility to ARDS.^11,12^ Metabolic studies^13,14^ of ARDS have expanded rapidly during the past decade, and candidates, such as proline, lysine, arginine, taurine, threonine, and glutamate, have been linked to ARDS risk. Metabolomics studies may aid in early diagnosis and ARDS severity assessment.^15^

Observational epidemiology studies, in contrast to randomized clinical trials, cannot infer a causal relationship because of potential unmeasured confounders and the unclear chronologic relationship between exposure and outcome.^16^ Mendelian randomization (MR) analysis integrates genetic variants as instrumental variables (IVs) and evaluates the association between exposure and outcome using summary-level data from observational studies^17^ and has identified reliable risk factors for various diseases.^18^

We used a 2-stage study design to identify novel metabolites associated with ARDS risk and survival. In the discovery stage, we performed MR analysis based on summary-level data from genome-wide association studies (GWASs) incorporating multiple single-nucleotide variations (SNVs) as IVs to evaluate systematically the association between blood metabolites and ARDS risk on a metabolome-wide scale. Candidate metabolites were replicated in an independent case-control study nested in the Molecular Epidemiology of ARDS (MEARDS) cohort.

## Methods

### Study Populations

#### Metabolite Quantitative Trait Locus Analysis

For this cohort study, association results of quantitative trait locus (QTL) analysis of SNVs and corresponding peripheral blood metabolites were acquired from publicly accessible resources^19,20^ that contained statistics for 529 metabolites with *P* < 1.00 × 10^−5^ based on 7824 European adults from the TwinsUK and KORA (Cooperative Health Research in the Region of Augsburg) F4 studies. Sample demographic characteristics, metabolome and genome profiles, quality control, and statistical methods are summarized in eTable 1 in the Supplement. Briefly, metabolites were profiled by liquid chromatography and gas chromatography combined with tandem mass spectrometry in peripheral plasma or serum. Annotation of all metabolites and results of metabolite QTL (mQTL) analysis are presented in eTable 2 and eTable 3 in the Supplement, respectively. The study was reviewed and approved by the institutional review boards of Massachusetts General Hospital (MGH), Beth Israel Deaconess Medical Center (BIDMC), and Harvard School of Public Health. All participants or their surrogate caregivers provided written informed consent. All data were deidentified. The study followed the Strengthening the Reporting of Observational Studies in Epidemiology (STROBE) reporting guideline.

#### MEARDS GWAS

The MEARDS GWAS samples were selected from the MEARDS cohort. Patients were recruited in ICUs at MGH and BIDMC, both in Boston, Massachusetts, from January 1, 1998, to December 31, 2014. Data analysis was performed from December 9, 2018, to January 4, 2019. Study population and recruitment procedures were described previously.^21^ Briefly, eligible individuals enrolled in the MEARDS GWAS were critically ill patients admitted to the ICU with at least 1 predisposing condition for ARDS: bacteremia, sepsis, septic shock, trauma, pneumonia, multiple fractures, pulmonary contusion, aspiration, or massive blood transfusion of packed red blood cells. Patients were eligible for the study if they did not have any of the following: age younger than 18 years, HIV infection, diffuse alveolar hemorrhage, chronic lung diseases other than chronic obstructive pulmonary disease or asthma, directive to withhold intubation, immunosuppression not secondary to corticosteroid, treatment with granulocyte colony–stimulating factor, cytotoxic therapy, or solid organ or bone marrow transplant. We collected demographic characteristics, medical history, vital signs, and hematologic and biochemical indicators and performed frequent arterial blood gas analyses and chest radiography within 24 hours after admission. Individuals were followed up daily for ARDS development defined by the Berlin criteria, requiring fulfillment of chest radiography and oxygenation criteria on the same calendar day during invasive ventilatory assistance.^22^ Participants from the control group were identified as at-risk patients who did not meet the criteria for ARDS during ICU stays and had no history of ARDS.

The MEARDS GWAS is part of the Identification of SNPs Predisposing to Altered Acute Lung Injury Risk (iSPAAR) consortium GWAS, which accounts for all at-risk control participants and approximately 40% of ARDS cases in the iSPAAR GWAS. DNA samples were genotyped using Illumina Human660W-Quad. version 1 A BeadChip (Illumina Inc). Standard quality control and imputation procedures were performed in all iSPAAR GWAS samples (eFigure 1 in the Supplement). Briefly, we excluded individuals with low call rates (<95%), familial relationships, or extreme heterozygosity rates. SNVs with minor allele frequencies less than 1%, low call rates (<95%), and *P* < 1 × 10^−6^ in Hardy-Weinberg equilibrium tests were excluded. After strict quality control, 983 patients with ARDS and 1227 at-risk patients without ARDS with complete clinical information from iSPAAR were retained. Whole genome imputation was performed with Minimac3 using the 1000 Genome Phase 3, version 5 database with 2504 individuals as reference.^23^ Imputed SNVs with *P* < 10^−6^ on the Hardy-Weinberg equilibrium test with imputation quality *r*^2^ < 0.6 and minor allele frequencies less than 1% were excluded as well. Finally, 9 047 773 SNVs were retained for analysis. To maintain homogeneity between cases and controls, 403 patients with ARDS and 1227 at-risk patients in the control group from the MEARDS cohort were included in the study analyzed through 2018 (Table). Association of SNVs with risk of ARDS was estimated by single variant logistic regression with adjustment for common covariates, including age, sex, sepsis, pneumonia, and the top 5 principle components that represented potential population structure.

**Table.** Demographic and Clinical Characteristics of the Study Population in Discovery and Validation Stages

Characteristic	MEARDS GWAS population at the discovery stage			Metabolomics study population at the validation stage		
	Patients with ARDS, No. (%) (n = 403)	At-risk patients without ARDS, No. (%) (n = 1227)	*P* value	Patients with ARDS, No. (%) (n = 83)	At-risk patients without ARDS, No. (%) (n = 83)	*P* value
Age, mean (SD), y	63.0 (17.0)	62.3 (16.9)	.06	57.0 (17.5)	57.4 (16.9)	.78
Sex						
Male	251 (62.3)	753 (61.4)	.79	59 (71.1)	59 (71.1)	>.99
Female	152 (37.7)	474 (38.6)		24 (28.9)	24 (28.9)	
Sepsis						
No	31 (7.7)	258 (21.0)	1.90 × 10^−9^	3 (3.6)	23 (27.7)	4.96 × 10^−5^
Yes	372 (92.3)	969 (79.0)		80 (96.4)	60 (72.3)	
Aspiration						
No	368 (91.3)	1151 (93.8)	.11	78 (94.0)	76 (91.6)	.76
Yes	35 (8.7)	76 (6.2)		5 (6.0)	7 (8.4)	
Pneumonia						
No	118 (29.3)	718 (58.5)	4.05 × 10^−24^	23 (24.0)	52 (62.6)	1.66 × 10^−6^
Yes	285 (70.7)	509 (41.5)		60 (76.0)	31 (37.3)	
Immune suppression						
No	378 (93.8)	1119 (91.2)	.12	75 (90.4)	73 (88.0)	.80
Yes	25 (6.2)	108 (8.8)		8 (9.6)	10 (12.0)	
Trauma						
No	395 (98.0)	1177 (95.9)	.07	80 (96.4)	78 (94.0)	.72
Yes	8 (2.0)	50 (4.1)		3 (3.6)	5 (6.0)	
Transfusion						
No	362 (89.8)	1198 (97.6)	5.09 × 10^−11^	76 (91.6)	82 (98.8)	.07
Yes	41 (10.2)	29 (2.4)		7 (8.4)	1 (1.2)	
APACHE III score, mean (SD)	66.0 (20.1)	52.05 (19.6)	1.69 × 10^−39^	64.0 (24.0)	49.76 (19.9)	3.34 × 10^−6^
Mortality						
28-d						
No	278 (69.0)	NA	NA	53 (63.9)	NA	NA
Yes	125 (31.0)	NA	NA	30 (36.1)	NA	NA
60-d						
No	254 (63.0)	NA	NA	52 (62.6)	NA	NA
Yes	149 (37.0)	NA	NA	31 (37.4)	NA	NA

Abbreviations: APACHE, Acute Physiology and Chronic Health Evaluation; ARDS, acute respiratory distress syndrome; GWAS, genome-wide association study; MEARDS, Molecular Epidemiology of ARDS; NA, not applicable.

### Functional Validation Analysis

To verify candidate metabolites, serum samples collected at the time of recruitment from an independent case-control study that included 83 patients with ARDS and age- and sex-matched at-risk patients without ARDS nested in MEARDS but not overlapping with the samples in the MEARDS GWAS were pooled for metabolomics testing. Demographic and clinical descriptions of the study population are listed in the Table.

Candidate metabolites were quantified by Metabolon Inc. Samples were prepared using the automated MicroLab STAR system (Hamilton Co). Several recovery standards were added before the first step in the extraction process for quality control purposes. Sample extracts were stored overnight in liquid nitrogen before analysis. Instrument variability (relative SD, 4%) and overall process variability (relative SD, 8%) met Metabolon’s acceptance criteria (eAppendix in the Supplement).

### Statistical Analysis

**Figure 1.**
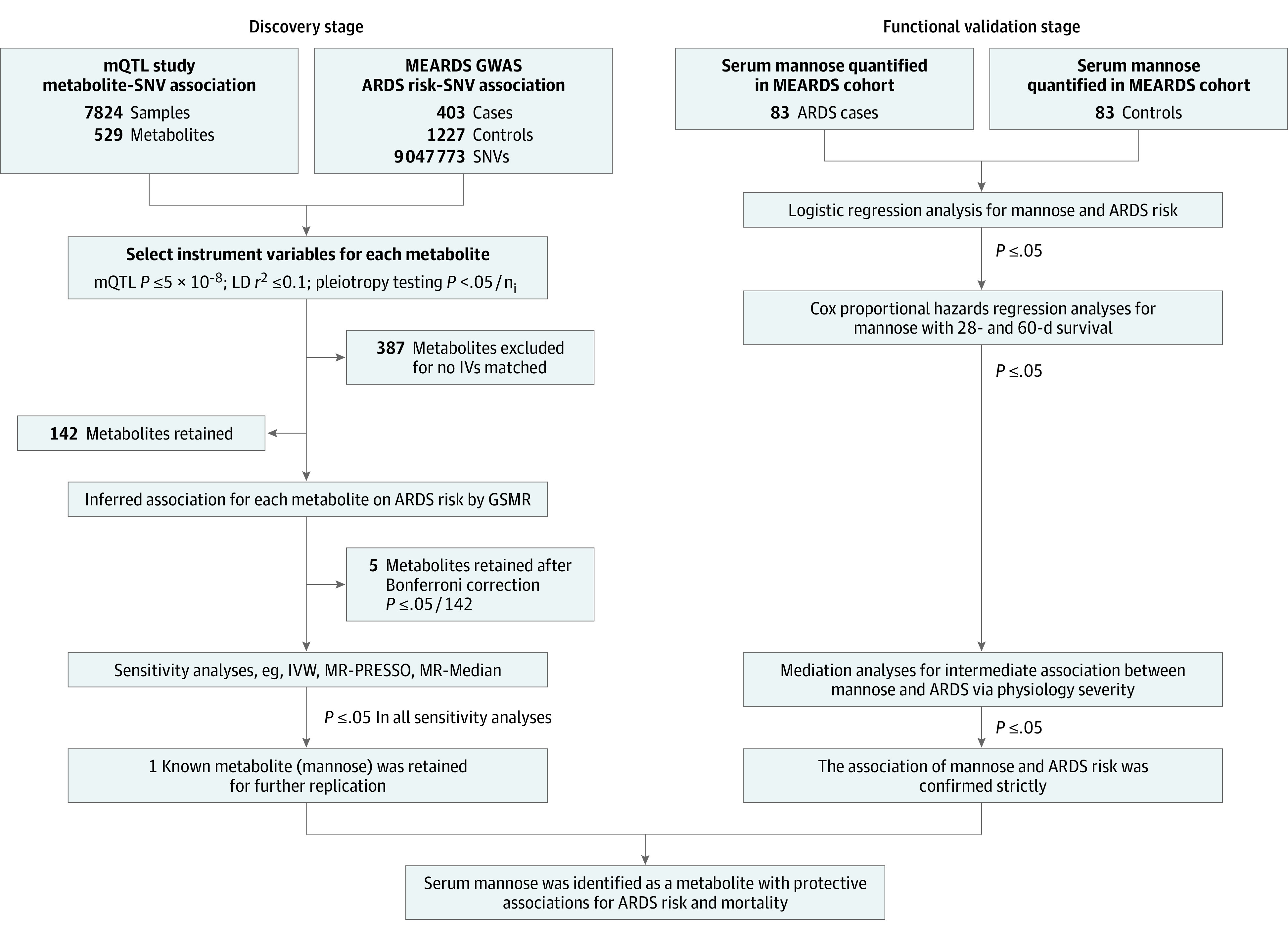
Study Workflow ARDS indicates acute respiratory distress syndrome; GSMR, generalized summary databased mendelian randomization; GWAS, genome-wide association study; IV, instrumental variable; IVW, inverse variance weighted; LD, linkage disequilibrium; MEARDS, Molecular Epidemiology of ARDS; mQTL, metabolite quantitative trait locus; MR, mendelian randomization; MR-PRESSO, MR Pleiotropy Residual Sum and Outlier; SNV, single-nucleotide variant.

The statistical analysis workflow is presented in Figure 1. Baseline characteristics of patients with ARDS and at-risk patients without ARDS at the discovery and validation stages were compared using the unpaired, 2-tailed *t* test or Mann-Whitney test for continuous variables depending on the data distribution, and the χ^2^ test was used for categorical variables. Continuous variables are presented as mean (SD); categorical variables are presented as number (percentage). In the discovery stage, a generalized summary databased MR (GSMR) analysis method was used to explore the associations or alterations in metabolites and ARDS risk with summary-level data derived from the mQTL analysis and MEARDS GWAS.^24,25^ The GSMR algorithms and workflow are summarized in eFigure 2 and the eAppendix in the Supplement. First, SNVs associated with each metabolite (*P* ≤ 5 × 10^−8^ for mQTL) were identified as genetic IVs and further examined for potential pleiotropic effects by heterogeneity in dependent instruments outlier detection (threshold *P* = .01).^24^ Second, association of these genetic IVs and ARDS risk was analyzed in the ARDS GWAS. Third, GSMR analysis was performed to evaluate the association between metabolites and ARDS risk (eFigure 3 in the Supplement). In addition, linkage disequilibrium among genetic IVs was estimated in the MEARDS GWAS data set and incorporated into the GSMR analysis to filter out nonindependent (linkage disequilibrium *r*^2^ > 0.1) SNVs. The association between metabolites and ARDS risk was measured by odds ratios (ORs) per SD and the corresponding 95% CI. Bonferroni correction was used to control for multiple testing. The significance level was set at *P* ≤ .05 divided by 142, resulting in *P* ≤ 3.52 × 10^−4^. Fourth, we also performed 3 additional sensitivity analyses at the discovery stage: (1) including all participants from the iSPAAR cohort in genome association analysis; (2) including all cases from the ARDS Network cohort and control group patients from the MEARDS cohort in genome association analysis; and (3) including only MEARDS GWAS data for patients with ARDS and at-risk patients without mild acute lung injury (ALI). In addition, we performed sensitivity analyses using a series of comparable MR methods to verify the robustness of results, including inverse variance weighted,^26^ MR Pleiotropy Residual Sum and Outlier (MR-PRESSO),^27^ MR weighted median,^28^ MR maximum likelihood,^29^ MR mode-based estimation,^30^ and MR Robust Adjusted Profile Score (MR-RAPS).^31^

In the functional validation stage, logistic regression validated the association between the candidate metabolite (mannose) and ARDS risk using individual-level data of 83 pairs of patients with ARDS and at-risk patients, adjusted for common covariates, including age, sex, sepsis, and pneumonia at ICU recruitment. To further explore nonlinear associations between mannose and ARDS risk, restricted cubic spline was used with 4 knots at the 20th, 40th, 60th, and 80th percentiles. The 10th percentile of mannose was the reference in corresponding curves.

The Acute Physiology and Chronic Health Evaluation (APACHE) III Score describes the degree of physiologic severity or multiple organ dysfunction. It represents a surrogate of disease severity and is well recognized as an important prognostic factor of clinical outcomes for critically ill patients in the ICU.^32^ The association between mannose and the APACHE III score was evaluated using a linear regression model. The association of mannose at ICU recruitment with both 28- and 60-day mortality of patients with ARDS was evaluated by logistic regression followed by survival analysis using a log-rank test and Cox proportional hazards regression with adjustment for common covariates as above. The indirect associations of mannose with ARDS risk and 28- or 60-day mortality via the APACHE III score were evaluated by mediation analysis.

Associations between SNVs and ARDS risk were analyzed using PLINK, version 1.9. All other statistical analyses were performed using R Software, version 3.5.1 (R Foundation for Statistical Computing).

## Results

### Characteristics of the Cohort Study Population

Of the 1630 participants from MEARDS who were admitted to the ICU, 403 participants (24.7%) were diagnosed with ARDS (mean [SD] age, 63.0 [17.0] years; 251 [62.3%] male) and 1227 (75.3%) were at-risk without ARDS (mean [SD] age, 62.3 [16.9] years; 753 [61.4%] male) (Table). The APACHE III scores were significantly higher for patients with ARDS than the at-risk patients without ARDS. The incidences of sepsis, pneumonia, and transfusion were higher among participants diagnosed as having ARDS than among participants in the control group (Table). Of the patients with ARDS, 125 (31.0%) died within 28 days and 149 (37.0%) died within 60 days of ICU admission.

### Serum Metabolites and ARDS Risk in the Discovery Stage

**Figure 2.**
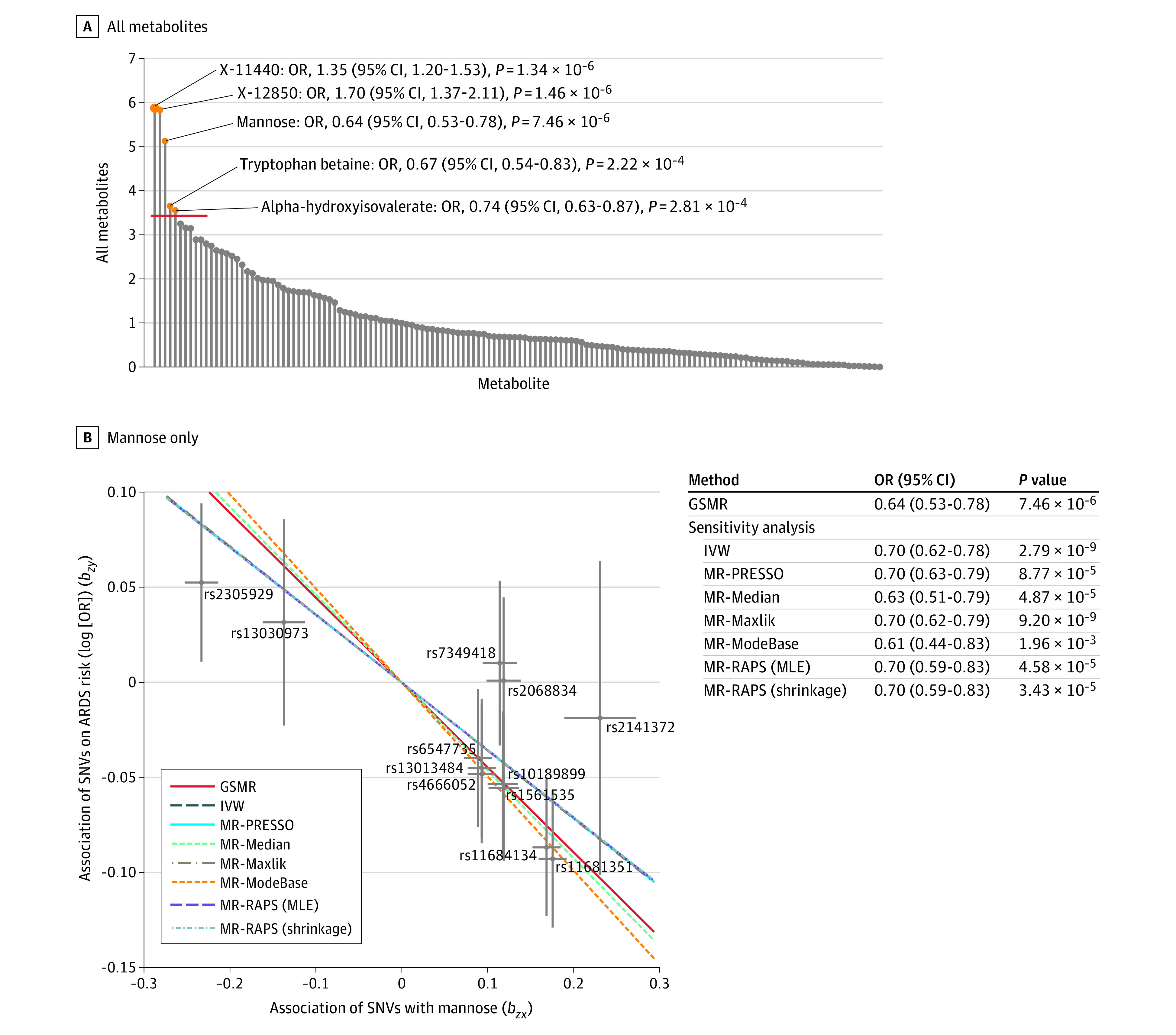
Associations Between Metabolites and Acute Respiratory Distress Syndrome (ARDS) Risk A, Associations between all metabolites and ARDS risk inferred by mendelian randomization (MR) analysis. B, Association between mannose and ARDS risk was inferred using different MR analytical methods for association between genetic variants and metabolites, including generalized summary databased MR (GSMR) analysis, inverse variance weighted (IVW); MR Pleiotropy Residual Sum and Outlier (MR-PRESSO), MR-Median, MR maximum likelihood (MR-Maxlik), MR mode-based estimation (MR-ModeBase), MR Robust Adjusted Profile Score (MR-RAPS), maximum likelihood estimate (MLE), and MR-RAPS (shrinkage). Each cross represents an instrumental variable. Error bars indicate 95% CIs. There are 12 instrumental variables in total. The lines with different color were estimated by different MR methods; the slope of each line represents the estimated association effect between mannose and ARDS risk of different MR methods. OR indicates odds ratio; SNVs, single-nucleotide variants.

In the discovery stage, 8947 SNVs with *P* ≤ 5 × 10^−8^ for mQTL were selected as genetic IVs based on summary-level mQTL analysis (eTable 3 in the Supplement). Associations between genetic IVs and ARDS risk are listed in eTable 4 in the Supplement. In MR analysis using summary-level data, 5 metabolites were significantly associated with ARDS risk (*P* ≤ 3.5 × 10^−4^) (Figure 2A). Three retained nominal statistical significance after a series of sensitivity analyses (Figure 2B and eFigure 4 in the Supplement). Mannose was identified as a potential protective factor for ARDS risk (OR, 0.64; 95% CI, 0.53-0.78; *P* = 1.46 × 10^−6^) (Figure 2B). However, the other 2 metabolites with unknown chemical identity were associated with increased ARDS risk (metabolite X-11440: OR, 1.35; 95% CI, 1.20-1.53; *P* = 1.34 × 10^−6^; metabolite X-12850: OR, 1.70; 95% CI, 1.37-2.11; *P* = 7.46 × 10^−6^) (eTable 5 in the Supplement). In addition, we also performed a sensitivity analysis and used a series of comparable MR methods to show that the significant GSMR results were robust under different data sets and different MR approaches (eTable 6 in the Supplement). We also conducted sensitivity analyses to test for the influence of horizontal pleiotropy (Egger regression for MR [MR-Egger], MR weighted median, MR-PRESSO, and MR-RAPS). The MR-Egger model was performed, and its intercept, representing horizontal pleiotropy, was not significant (MR-Egger intercept *P* > .06). In addition, MR-PRESSO and MR-RAPS models found no evidence of horizontal pleiotropy.

### Serum Mannose and ARDS Risk in the Functional Validation Stage

**Figure 3.**
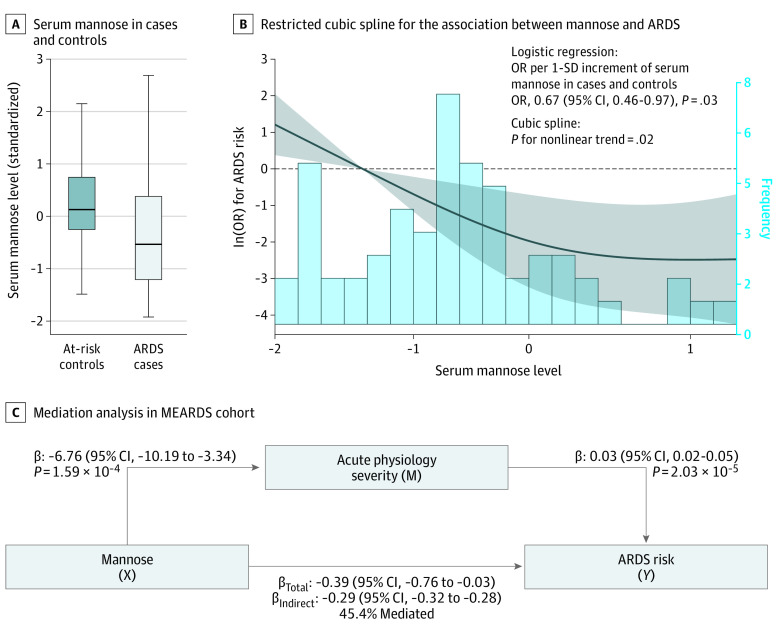
Serum Mannose in Patients Without Acute Respiratory Distress Syndrome (ARDS) and Patients With ARDS in the Validation Stage A, Box plot depicting serum mannose in patients with ARDS and patients without ARDS in the validation study. Boxes show the first quartile (lower border of the box), median (horizontal line within the box), and third quartile (top border of the box). Whiskers show the range of nonoutlier values. B, Restricted cubic spline for the association between mannose and ARDS in the validation study. The line represents adjusted odds ratios (ORs) based on restricted cubic spline for log-transformed level of serum mannose in the logistic model. The dashed line represents the reference line (ln(OR)=0). Knots were placed at the 20th, 40th, 60th, and 80th percentiles of serum mannose distribution, and the reference value was set at the 10th percentile. C, Mediation analysis shows the association among mannose, acute physiologic status, and ARDS risk in the validation study. The β values and 95% CIs represent indirect effects of mannose on ARDS risk mediated through acute physiologic status, and the percentage of mannose on ARDS was mediated by affecting acute physiologic status. The acute physiologic severity at intensive care unit admission was represented by the Acute Physiology and Chronic Health Evaluation III score. MEARDS indicates Molecular Epidemiology of ARDS.

In the functional validation stage, serum mannose was quantified in an independent set of 83 pairs of patients with ARDS and at-risk patients without ARDS in the control group. Serum mannose levels were significantly lower in patients with ARDS than in those in the control group (*t* = 3.07; *P* = 2.5 × 10^−3^) (Figure 3A). The association was confirmed by logistic regression (OR, 0.64; 95% CI, 0.47-0.89; *P* = .01) and retained significance after adjustment for age, sex, sepsis, and pneumonia (OR, 0.67; 95% CI, 0.46-0.97; *P* = .03). In addition, restricted cubic spline analysis indicated a nonlinear association between serum mannose and ARDS risk (χ^2^_v = 1_ = 5.24; *P* = .02) (Figure 3B).

### Serum Mannose and Degree of Acute Physiologic Severity

Interestingly, MR analysis suggested an association between mannose and degree of acute physiologic severity at ICU admission, as represented by the APACHE III score (β = −0.28 per 1-SD increment; 95% CI, −0.37 to −0.19; *P* = 5.38 × 10^−10^) (eFigure 5 in the Supplement). Furthermore, mediation analysis indicated that 45.4% of the total protective association of mannose with ARDS risk was mediated via attenuating baseline acute physiologic severity (b = −0.29 for indirect association; 95% CI, −0.32 to −0.28; *P* = 1.14 × 10^−17^) (Figure 3C).

### Serum Mannose, Acute Physiologic Severity, and Mortality

**Figure 4.**
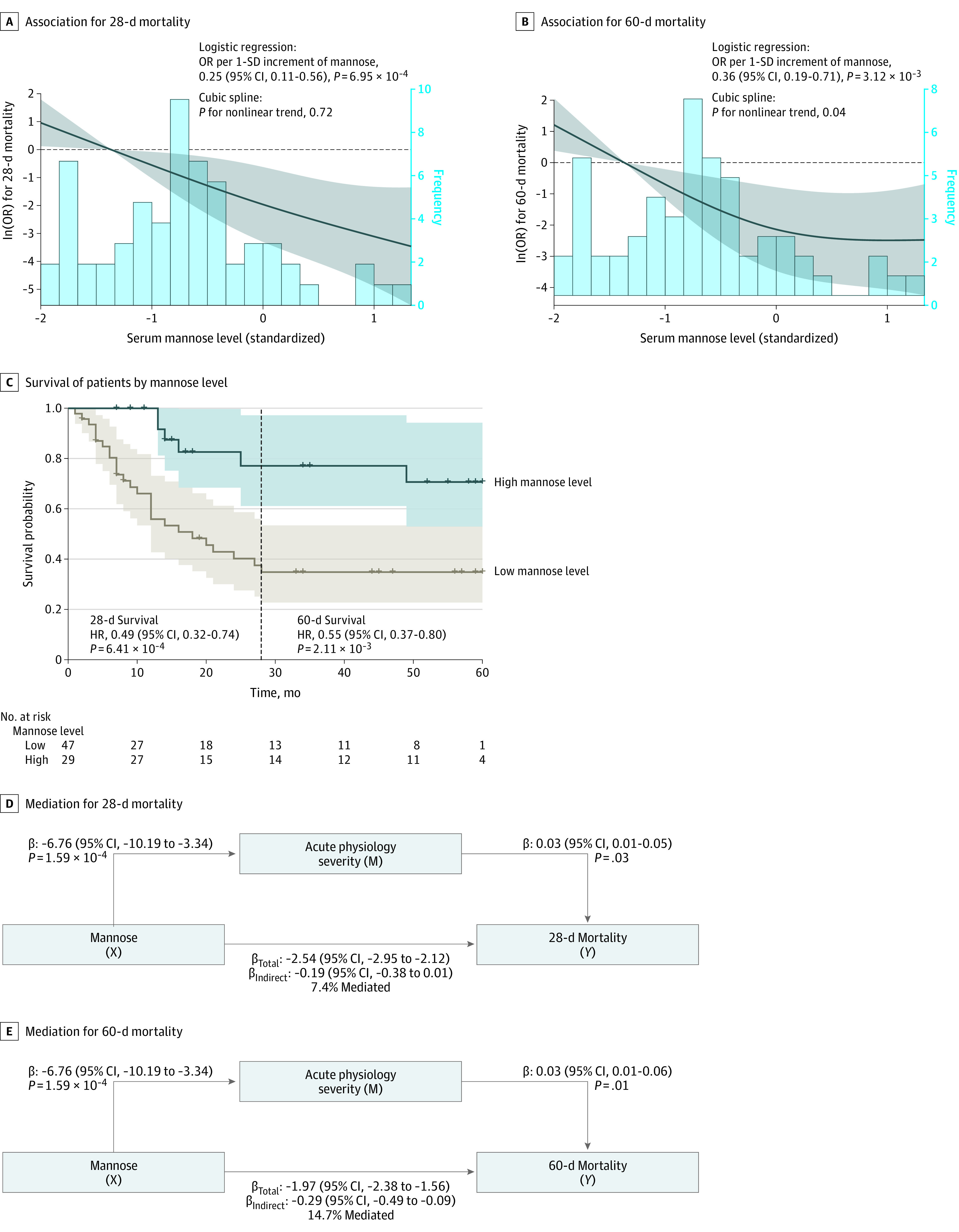
Nonlinear Association Between Mannose and 28-Day Mortality or 60-Day Mortality Risk in the Functional Validation Study A and B, Restricted cubic spline for the association between serum mannose and 28-day mortality (A) or 60-day mortality (B) in the validation study. The dashed line is the reference line. C, Kaplan-Meier survival curves showing 28-day and 60-day survival of patients with high-level mannose versus low-level mannose. D and E, Mediation analyses showing the associations among mannose, acute physiology status, and 28-day mortality (D) and 60-day mortality (E) in the functional validation study. HR indicates hazard ratio; OR, odds ratio.

The apparent protective association of mannose on both 28-day (OR_,_ 0.25; 95% CI, 0.11-0.56; *P* = 6.95 × 10^−4^) (Figure 4A) and 60-day (OR, 0.36; 95% CI, 0.19-0.71; *P* = 3.12 × 10^−3^) (Figure 4B) mortality was functionally validated in 83 patients with ARDS using a logistic regression model adjusted for age, sex, sepsis, and pneumonia. Compared with patients with serum mannose levels less than the median (low-level group), patients with serum mannose levels greater than the median (high-level group) had significantly improved survival at 28 days (hazard ratio [HR], 0.49; 95% CI, 0.32-0.74; *P* = 6.41 × 10^−4^) and 60 days (HR, 0.55; 95% CI, 0.37-0.80; *P* = 2.11 × 10^−3^) (Figure 4C) after adjustment for all potential confounders. A portion of these apparent protective associations was mediated by attenuated baseline acute physiologic severity (7.4% mediated for 28-day mortality and 14.7% mediated for 60-day mortality) (Figure 4D and 4E). To evaluate whether the apparent association of serum mannose with outcomes was mediated by acute physiologic severity, the models were additionally adjusted for the degree of baseline acute physiologic severity. Serum mannose retained a significant direct association with 28-day (OR, 0.28; 95% CI, 0.11-0.57; *P* = 1.65 × 10^−3^) and 60-day (OR, 0.40; 95% CI, 0.19-0.78; *P* = .01) mortality and 28-day (HR_,_ 0.50; 95% CI, 0.08-0.93; *P* = 1.42 × 10^−3^) and 60-day (HR_,_ 0.58; 95% CI, 0.39-0.85; *P* = 5.36 × 10^−3^) survival for patients with ARDS.

## Discussion

To the best of our knowledge, this cohort study is the first metabolome-wide association study on ARDS risk and mortality and incorporates the largest sample size at the discovery stage (n = 7824 in the mQTL analysis and n = 1630 in the GWAS) followed by an independent functional validation (n_v_ = 166). The findings of this study indicated that serum mannose may be a protective metabolite regarding ARDS risk and survival.

Dozens of articles in the literature revealed a body of research on candidate metabolites showing potential associations with ARDS outcomes. However, most associations were identified in observational studies, and despite efforts being made to control for observed confounders that can result in false associations in epidemiologic studies, unobserved or unknown confounders may still exist. Such confounders cannot be adjusted by traditional statistical models. Thus, results from observational studies may be biased by unmeasured confounders and a lack of evidence regarding causality.

This study overcomes some analytic limitations. The discovery stage of this study takes advantage of the strengths of MR analysis. By incorporating genetic variants as IVs, MR analysis appears to efficiently infer a relationship.^16^ A genetic IV was defined as an SNV that is relevant to the focused exposure (eg, mannose) but irrelevant to any confounders or has no effect on outcome other than through exposure.^33^ However, the main difficulty in MR analysis is the choice of IVs and assessment of the assumptions. If IV assumptions are violated, then inference will be unreliable.^34^ In this study, IVs were selected according to stringent Bonferroni correction to ensure the association with metabolites, which were further trimmed according to the correlations among IVs. Meanwhile, IVs with potential pleiotropic effects were excluded. In addition, the populations of mQTL and ARDS GWAS analyses were both of White/European ancestry, ensuring homogeneity of the target population. These procedures, to some extent, maximize the validity of genetic IVs in this study.

These findings suggest that serum mannose exhibits a protective effect not only on ARDS risk but also on ARDS mortality, indicating a complex role for mannose in ARDS development and progression. Inflammatory cells and inflammatory mediators have constituted ALI and ARDS inflammatory reactions and immunomodulating cellular networks and cytokine networks.^35^ They regulate and control inflammatory reaction by different signal transduction pathways. The damage caused by endotoxin during septicemia (lipopolysaccharide [LPS]) is the main cause of ALI and ARDS.^36^ Mannose, a simple hexose sugar with a molecular weight of 180.2 kDa, inhibits neutrophil oxidative bursts, playing an important role in inflammation.^37^ Mannose effectively reduces LPS-induced ALI.^38^ Furthermore, mannose pretreatment may reduce LPS induction by inhibiting activation of alveolar macrophages and subsequent inflammatory cytokines in lung injury, reducing production of proinflammatory cytokines, upregulating expression of peroxisome proliferator–activated receptor γ and mannose receptor, and downregulating expression of transforming growth factor β1.^39^ Similarly, Xu et al^38,39^ found that intravenous administration of mannose appears to ameliorate the increase of pulmonary capillary permeability in rats with LPS-induced ALI. In these animals, mannose reduced polymorphonuclear infiltration, blocked the increase in tumor necrosis factor levels, alleviated lung tissue neutrophil infiltration degree, suppressed capillary permeability, and reduced albumen exudation and edema caused by the lung injury. It also reduced the myeloperoxidase level of the bronchoalveolar lavage fluid supernatant and ameliorated the inflammatory and pathological changes in lung tissue.^38^ In addition, upregulation of mannose receptor expression is a major preventive response to ALI,^39^ which may have a synergistic effect with mannose administration.

### Strengths and Limitations

This study has several strengths. First, MR was used to infer the association between serum mannose and ARDS outcomes to avoid potential false associations caused by unmeasured confounders. The association of mannose with ARDS risk and mortality is robust based on a series of sensitivity analyses and independent validation. Second, multiple genetic IVs were used to further boost statistical power.^27^ Third, mediation analysis indicates that physiologic severity status may play a role in the contribution of mannose to ARDS risk and mortality.

The study also has some limitations. First, serum metabolites were tested only at ICU admission in the validation study. Serum metabolites are dynamic during ICU hospitalization and may reflect the development of disease. However, evaluation of mannose at an early time point is useful for prognostic evaluation and prevention.^40^ Second, several unknown metabolites were not further verified because of unclear molecular identity and mechanism. Third, the findings may not apply to populations other than White people.

## Conclusions

In this study, genetically regulated serum mannose appeared to be associated with ARDS risk and mortality. Increased serum mannose levels appeared to be associated with reduced ARDS risk and improved survival. The findings of this study may shed light on biomarkers and pathways for ARDS prevention and clinical intervention.

## Notes

Supplement. **eTable 1.** Descriptions of mQTL Study **eTable 2.** Annotation of Metabolites in mQTL Study **eTable 3.** Association Results Derived From mQTL Analysis of SNPs on Mannose With P Value <5×10^−8^ **eTable 4.** Association Between SNPs Selected as Genetic IV for Mannose and ARDS Risk in MEARDS GWAS **eTable 5.** Pearson’s Correlation Coefficient *r* Between Pairs of Metabolites (X-11440 and X-12850) Measured in Metabolome-wide Association Study **eTable 6.** Sensitivity Analyses for Association Between Mannose and ARDS Risk Using Different Mendelian Randomization Methods and Different Sources of Association Results Between Genetic Instrumental Variables (SNPs) and ARDS Risk **eFigure 1.** Standard Quality Control Procedure for GWAS Data **eFigure 2.** GSMR Analysis Workflow **eFigure 3.** Framework Diagram of Mendelian Randomization **eFigure 4.** Sensitivity Analysis for Candidate Metabolites Associated With ARDS Risk **eFigure 5.** Association Between Serum Mannose and Acute Physiology Severity Using GSMR **eAppendix.** Supplementary Methods
