# Supplementary material for: Association of Serum Mannose With Acute Respiratory Distress Syndrome Risk and Survival
Source: JAMA Netw Open. 2021 Jan 27;4(1):e2034569. doi: 10.1001/jamanetworkopen.2020.34569 (PMC7841460; doi:10.1001/jamanetworkopen.2020.34569)
Supplement: Supplement. — eTable 1. Descriptions of mQTL Study eTable 2. Annotation of Metabolites in mQTL Study eTable 3. Association Results Derived From mQTL Analysis of SNPs on Mannose With P Value <5×10^−8^ eTable 4. Association Between SNPs Selected as Genetic IV for Mannose and ARDS Risk in MEARDS GWAS eTable 5. Pearson’s Correlation Coefficient r Between Pairs of Metabolites (X-11440 and X-12850) Measured in Metabolome-wide Association Study eTable 6. Sensitivity Analyses for Association Between Mannose and ARDS Risk Using Different Mendelian Randomization Methods and Different Sources of Association Results Between Genetic Instrumental Variables (SNPs) and ARDS Risk eFigure 1. Standard Quality Control Procedure for GWAS Data eFigure 2. GSMR Analysis Workflow eFigure 3. Framework Diagram of Mendelian Randomization eFigure 4. Sensitivity Analysis for Candidate Metabolites Associated With ARDS Risk eFigure 5. Association Between Serum Mannose and Acute Physiology Severity Using GSMR eAppendix. Supplementary Methods [file jamanetwopen-e2034569-s001.pdf]

Supplementary Online Content

Wei Y, Huang H, Zhang R, et al. Association of serum mannose with acute respiratory distress syndrome risk and survival. *JAMA Netw Open*. 2021;4(1):e2034569. doi:10.1001/jamanetworkopen.2020.34569

eTable 1. Descriptions of mQTL Study

eTable 2. Annotation of Metabolites in mQTL Study

eTable 3. Association Results Derived From mQTL Analysis of SNPs on Mannose With P Value<5×10^{-8}

eTable 4. Association Between SNPs Selected as Genetic IV for Mannose and ARDS Risk in MEARDS GWAS

eTable 5. Pearson's Correlation Coefficient *r* Between Pairs of Metabolites (X-11440 and X-12850) Measured in Metabolome-wide Association Study

eTable 6. Sensitivity Analyses for Association Between Mannose and ARDS Risk Using Different Mendelian Randomization Methods and Different Sources of Association Results Between Genetic Instrumental Variables (SNPs) and ARDS Risk

eFigure 1. Standard Quality Control Procedure for GWAS Data

eFigure 2. GSMR Analysis Workflow

eFigure 3. Framework Diagram of Mendelian Randomization

eFigure 4. Sensitivity Analysis for Candidate Metabolites Associated With ARDS Risk

eFigure 5. Association Between Serum Mannose and Acute Respiratory Distress Syndrome (ARDS) Risk

eAppendix. Supplementary Methods

This supplementary material has been provided by the authors to give readers additional information about their work.

eTable 1. Descriptions of mQTL Study

Study Procedure	Study information	TwinsUK	KORA F4
Sample description	Country of origin	UK	Germany
	Number of samples (Male/Female)	6,056 (433/5,623)	1,768 (858/910)
	Age (years, mean±SD)	53.4±14.0	60.8±8.8
	BMI (kg/m^2, mean±SD)	26.1±4.9	28.2±4.8
Metabolite measurements	Platform	UPLC-MS/MS and GC-MS	UPLC-MS/MS and GC-MS
	Sample type (serum)	1,052	1,768
	Sample type (plasma)	5,004	0
	Number of metabolites (known/unknown)	296/207	305/212
QC of samples	Outlier exclusion	outlier exclusion with high missing rate (one sample excluded)	
QC metabolites	Data transformation	block correction, log-transformation	block correction, log-transformation
	Missing data	3 metabolites with <10 samples excluded	4 metabolites with <10 samples excluded
	Outlier exclusion	>4 SD from mean	>4 SD from mean
SNP genotyping	Platform	Illumina HumanHap300, 610Q, 1M-Duo, 1.2MDuo 1M	Affymetrix Genome-Wide Human SNP Array 6.0
	Calling algorithm	BeadStudio	Birdseed2
QC of SNPs	Sample Call Rate	98%	93%
	SNP Call Rate	97% (MAF ≥5%)	93%
		99% (1≤ MAF ≤5%)	
	P value of HWE test	>10^{-6}	NA
	MAF	>1%	NA
Imputation of SNPs	Number of autosomal SNPs	489,767	655,658
	Reference panel	NCBI Build 36 HapMap CEU HM2 rel22	NCBI Build 36
	Software	IMPUTE v2	IMPUTE
	MAF	>1%	>1%

© 2021 Wei Y et al. *JAMA Network Open.*

Study Procedure	Study information	TwinsUK	KORA F4
	Imputation quality score	>0.4	>0.4
	Imputation posterior probability	>90%	
Statistical analysis	Number of samples with genotypes	5,605 (MZ duplicated)	1,768
	Number of SNPs	3,039,184	2,163,597
	Number of metabolites	293/207	301/212
	Model	Additive	Additive
	Software	MERLIN	QUICKTEST
	Adjustments	Age, sex, batch of specimen and zygosity	Age and sex
Meta-analysis	Number of overlapped metabolites	486	
	Model	Inverse variance model	
	Software	METAL	

© 2021 Wei Y et al. *JAMA Network Open.*

eTable 2. Annotation of Metabolites in mQTL Study

ID	Metabolite	Status	Pathway	Super-pathway	Platform	Compound ID	Quant. Mass.	Retention Time	CV (%)	Total N
M32339	alanine	Known	Alanine and aspartate metabolism	Amino acid	GC/MS				13.40	7,788
M34283	asparagine	Known	Alanine and aspartate metabolism	Amino acid	GC/MS				27.90	7,761
M15996	aspartate	Known	Alanine and aspartate metabolism	Amino acid	GC/MS				29.00	7,721
M01585	N-acetylalanine	Known	Alanine and aspartate metabolism	Amino acid	LC/MS neg				12.10	7,720
M32348	2-aminobutyrate	Known	Butanoate metabolism	Amino acid	LC/MS pos				9.60	7,814
M27718	creatine	Known	Creatine metabolism	Amino acid	LC/MS pos				5.70	7,822
M00513	creatinine	Known	Creatine metabolism	Amino acid	LC/MS pos				9.40	7,810
M21044	2-hydroxybutyrate (AHB)	Known	Cysteine, methionine, SAM, taurine metabolism	Amino acid	GC/MS				7.40	7,815
M31453	cysteine	Known	Cysteine, methionine, SAM, taurine metabolism	Amino acid	GC/MS				21.70	7,692
M31454	cystine	Known	Cysteine, methionine, SAM, taurine metabolism	Amino acid	GC/MS				NA	1,409
M01302	methionine	Known	Cysteine, methionine, SAM, taurine metabolism	Amino acid	LC/MS neg				5.70	7,795
M32322	glutamate	Known	Glutamate metabolism	Amino acid	GC/MS				14.30	7,804
M00053	glutamine	Known	Glutamate metabolism	Amino acid	LC/MS pos				7.20	7,821
M32672	pyroglutamine*	Known	Glutamate metabolism	Amino acid	LC/MS pos				9.00	7,800
M01494	5-oxoproline	Known	Glutathione metabolism	Amino acid	LC/MS pos				20.80	7,802
M35159	cysteine-glutathione disulfide	Known	Glutathione metabolism	Amino acid	LC/MS pos				NA	1,997
M03141	betaine	Known	Glycine, serine and threonine metabolism	Amino acid	LC/MS pos				8.50	7,806
M32338	glycine	Known	Glycine, serine and threonine metabolism	Amino acid	GC/MS				13.90	7,802
M27710	N-acetylglycine	Known	Glycine, serine and threonine metabolism	Amino acid	GC/MS				19.00	7,135

© 2021 Wei Y et al. *JAMA Network Open.*

ID	Metabolite	Status	Pathway	Super-pathway	Platform	Compound ID	Quant. Mass.	Retention Time	CV (%)	Total N
M33939	N-acetylthreonine	Known	Glycine, serine and threonine metabolism	Amino acid	LC/MS neg				24.50	6,909
M32315	serine	Known	Glycine, serine and threonine metabolism	Amino acid	GC/MS				16.10	7,796
M01284	threonine	Known	Glycine, serine and threonine metabolism	Amino acid	LC/MS pos				12.60	6,020
M01558	4-acetamidobutanoate	Known	Guanidino and acetamido metabolism	Amino acid	LC/MS pos				17.30	6,930
M15677	3-methylhistidine	Known	Histidine metabolism	Amino acid	LC/MS neg				6.80	5,885
M00059	histidine	Known	Histidine metabolism	Amino acid	LC/MS neg				5.90	7,804
M35439	glutaroyl carnitine	Known	Lysine metabolism	Amino acid	LC/MS pos				10.90	7,701
M01301	lysine	Known	Lysine metabolism	Amino acid	LC/MS pos				16.60	7,812
M01444	pipecolate	Known	Lysine metabolism	Amino acid	LC/MS pos				7.60	7,792
M35635	3-(3-hydroxyphenyl)propionate	Known	Phenylalanine & tyrosine metabolism	Amino acid	LC/MS neg				0.00	1,163
M32197	3-(4-hydroxyphenyl)lactate	Known	Phenylalanine & tyrosine metabolism	Amino acid	LC/MS neg				6.90	7,795
M12017	3-methoxytyrosine	Known	Phenylalanine & tyrosine metabolism	Amino acid	LC/MS pos				21.60	5,997
M15749	3-phenylpropionate (hydrocinnamate)	Known	Phenylalanine & tyrosine metabolism	Amino acid	LC/MS neg				17.10	6,182
M00541	4-hydroxyphenylacetate	Known	Phenylalanine & tyrosine metabolism	Amino acid	GC/MS				17.90	387
M36103	p-cresol sulfate	Known	Phenylalanine & tyrosine metabolism	Amino acid	LC/MS neg				4.80	7,758
M32553	phenol sulfate	Known	Phenylalanine & tyrosine metabolism	Amino acid	LC/MS neg				5.70	7,810
M15958	phenylacetate	Known	Phenylalanine & tyrosine metabolism	Amino acid	LC/MS neg				20.30	4,754
M35126	phenylacetylglutamine	Known	Phenylalanine & tyrosine metabolism	Amino acid	LC/MS pos				6.90	7,812
M00064	phenylalanine	Known	Phenylalanine & tyrosine metabolism	Amino acid	LC/MS pos				5.90	7,803
M22130	phenyllactate (PLA)	Known	Phenylalanine & tyrosine metabolism	Amino acid	LC/MS neg				17.50	6,064

© 2021 Wei Y et al. *JAMA Network Open.*

ID	Metabolite	Status	Pathway	Super-pathway	Platform	Compound ID	Quant. Mass.	Retention Time	CV (%)	Total N
M01299	tyrosine	Known	Phenylalanine & tyrosine metabolism	Amino acid	LC/MS pos				6.10	7,807
M27672	3-indoxyl sulfate	Known	Tryptophan metabolism	Amino acid	LC/MS neg				6.60	7,787
M32675	C-glycosyltryptophan*	Known	Tryptophan metabolism	Amino acid	LC/MS pos				8.50	7,786
M27513	indoleacetate	Known	Tryptophan metabolism	Amino acid	LC/MS pos				12.50	7,618
M18349	indolelactate	Known	Tryptophan metabolism	Amino acid	LC/MS pos				24.00	7,378
M32405	indolepropionate	Known	Tryptophan metabolism	Amino acid	LC/MS pos				17.00	7,803
M15140	kynurenine	Known	Tryptophan metabolism	Amino acid	LC/MS pos				7.30	7,816
M02342	serotonin (5HT)	Known	Tryptophan metabolism	Amino acid	LC/MS pos				27.10	6,139
M00054	tryptophan	Known	Tryptophan metabolism	Amino acid	LC/MS pos				6.40	7,804
M37097	tryptophan betaine	Known	Tryptophan metabolism	Amino acid	LC/MS Pos				7.20	7,439
M01638	arginine	Known	Urea cycle; arginine-, proline-, metabolism	Amino acid	LC/MS neg				16.80	7,528
M02132	citrulline	Known	Urea cycle; arginine-, proline-, metabolism	Amino acid	LC/MS pos				9.50	7,773
M36808	dimethylarginine (SDMA + ADMA)	Known	Urea cycle; arginine-, proline-, metabolism	Amino acid	LC/MS pos				14.70	7,347
M22138	homocitrulline	Known	Urea cycle; arginine-, proline-, metabolism	Amino acid	LC/MS pos				26.00	4,135
M15630	N-acetylornithine	Known	Urea cycle; arginine-, proline-, metabolism	Amino acid	LC/MS pos				23.20	7,574
M01493	ornithine	Known	Urea cycle; arginine-, proline-, metabolism	Amino acid	LC/MS pos				22.90	7,746
M01898	proline	Known	Urea cycle; arginine-, proline-, metabolism	Amino acid	LC/MS pos				6.20	7,816
M32319	trans-4-hydroxyproline	Known	Urea cycle; arginine-, proline-, metabolism	Amino acid	GC/MS				10.90	7,802
M01670	urea	Known	Urea cycle; arginine-, proline-, metabolism	Amino acid	GC/MS				12.60	7,796
M22030	2-hydroxyisobutyrate	Known	Valine, leucine and isoleucine metabolism	Amino acid	GC/MS				22.00	6,539
M35431	2-methylbutyroylcarnitine	Known	Valine, leucine and isoleucine metabolism	Amino acid	LC/MS pos				15.60	7,420

© 2021 Wei Y et al. *JAMA Network Open.*

ID	Metabolite	Status	Pathway	Super-pathway	Platform	Compound ID	Quant. Mass.	Retention Time	CV (%)	Total N
M32397	3-hydroxy-2-ethylpropionate	Known	Valine, leucine and isoleucine metabolism	Amino acid	GC/MS				18.90	889
M21047	3-methyl-2-oxobutyrate	Known	Valine, leucine and isoleucine metabolism	Amino acid	LC/MS neg				18.50	7,648
M15676	3-methyl-2-oxovalerate	Known	Valine, leucine and isoleucine metabolism	Amino acid	LC/MS neg				6.70	7,779
M22116	4-methyl-2-oxopentanoate	Known	Valine, leucine and isoleucine metabolism	Amino acid	LC/MS neg				7.10	7,776
M33937	alpha-hydroxyisovalerate	Known	Valine, leucine and isoleucine metabolism	Amino acid	LC/MS neg				9.00	7,668
M12129	beta-hydroxyisovalerate	Known	Valine, leucine and isoleucine metabolism	Amino acid	LC/MS neg				18.80	7,283
M35433	hydroxyisovaleroyl carnitine	Known	Valine, leucine and isoleucine metabolism	Amino acid	LC/MS pos				33.30	5,588
M33441	isobutyrylcarnitine	Known	Valine, leucine and isoleucine metabolism	Amino acid	LC/MS pos				9.60	7,812
M01125	isoleucine	Known	Valine, leucine and isoleucine metabolism	Amino acid	LC/MS pos				6.20	7,801
M34407	isovalerylcarnitine	Known	Valine, leucine and isoleucine metabolism	Amino acid	LC/MS pos				17.20	7,789
M00060	leucine	Known	Valine, leucine and isoleucine metabolism	Amino acid	LC/MS pos				6.10	7,799
M22177	levulinate (4-oxovalerate)	Known	Valine, leucine and isoleucine metabolism	Amino acid	LC/MS pos or neg				28.60	6,982
M35428	tiglyl carnitine	Known	Valine, leucine and isoleucine metabolism	Amino acid	LC/MS pos				24.30	1,328
M01649	valine	Known	Valine, leucine and isoleucine metabolism	Amino acid	LC/MS pos				6.20	7,808
M33477	erythronate*	Known	Aminosugars metabolism	Carbohydrate	GC/MS				13.80	7,752
M27722	erythrose	Known	Fructose, mannose, galactose, starch, and sucrose metabolism	Carbohydrate	GC/MS				40.70	7,263
M00577	fructose	Known	Fructose, mannose, galactose, starch, and sucrose metabolism	Carbohydrate	GC/MS				22.58	7781
M15335	mannitol	Known	Fructose, mannose, galactose, starch, and sucrose metabolism	Carbohydrate	GC/MS				30.45	5917
M00584	mannose	Known	Fructose, mannose, galactose, starch, and sucrose metabolism	Carbohydrate	GC/MS				17.24	7793

© 2021 Wei Y et al. *JAMA Network Open.*

ID	Metabolite	Status	Pathway	Super-pathway	Platform	Compound ID	Quant. Mass.	Retention Time	CV (%)	Total N
M20675	1,5-anhydroglucitol (1,5-AG)	Known	Glycolysis, gluconeogenesis, pyruvate metabolism	Carbohydrate	LC/MS neg				6.70	7,746
M21049	1,6-anhydroglucose	Known	Glycolysis, gluconeogenesis, pyruvate metabolism	Carbohydrate	GC/MS				17.81	3663
M20489	glucose	Known	Glycolysis, gluconeogenesis, pyruvate metabolism	Carbohydrate	GC/MS				12.70	7,773
M01572	glycerate	Known	Glycolysis, gluconeogenesis, pyruvate metabolism	Carbohydrate	GC/MS				10.84	7778
M00527	lactate	Known	Glycolysis, gluconeogenesis, pyruvate metabolism	Carbohydrate	GC/MS				6.70	7,814
M00599	pyruvate	Known	Glycolysis, gluconeogenesis, pyruvate metabolism	Carbohydrate	GC/MS				30.31	7682
M00575	arabinose	Known	Nucleotide sugars, pentose metabolism	Carbohydrate	GC/MS				25.70	5,889
M15964	arabitol	Known	Nucleotide sugars, pentose metabolism	Carbohydrate	GC/MS				18.91	1744
M35854	threitol	Known	Nucleotide sugars, pentose metabolism	Carbohydrate	GC/MS				23.00	7,381
M01640	ascorbate (Vitamin C)	Known	Ascorbate and aldarate metabolism	Cofactors and vitamins	GC/MS				0.00	2085
M27738	threonate	Known	Ascorbate and aldarate metabolism	Cofactors and vitamins	GC/MS				13.90	7,782
M32586	bilirubin (E,E)*	Known	Hemoglobin and porphyrin metabolism	Cofactors and vitamins	LC/MS pos				24.80	7748
M34106	bilirubin (E,Z or Z,E)*	Known	Hemoglobin and porphyrin metabolism	Cofactors and vitamins	LC/MS pos				29.20	5,295
M27716	bilirubin (Z,Z)	Known	Hemoglobin and porphyrin metabolism	Cofactors and vitamins	LC/MS neg				36.80	6812
M32593	heme*	Known	Hemoglobin and porphyrin metabolism	Cofactors and vitamins	LC/MS pos				24.40	6,380
M02137	biliverdin	Known	Hemoglobin and porphyrin metabolism	Cofactors and vitamins	LC/MS neg				18.65	6686
M32401	trigonelline (N'-methylnicotinate)	Known	Nicotinate and nicotinamide metabolism	Cofactors and vitamins	LC/MS pos				13.90	739
M01508	pantothenate	Known	Pantothenate and CoA metabolism	Cofactors and vitamins	LC/MS pos				15.03	7604
M01827	riboflavin (Vitamin B2)	Known	Riboflavin metabolism	Cofactors and vitamins	LC/MS pos				NA	305

© 2021 Wei Y et al. *JAMA Network Open.*

ID	Metabolite	Status	Pathway	Super-pathway	Platform	Compound ID	Quant. Mass.	Retention Time	CV (%)	Total N
M01561	alpha-tocopherol	Known	Tocopherol metabolism	Cofactors and vitamins	GC/MS				16.71	7725
M33420	gamma-tocopherol	Known	Tocopherol metabolism	Cofactors and vitamins	GC/MS				19.70	6,226
M31555	pyridoxate	Known	Vitamin B6 metabolism	Cofactors and vitamins	LC/MS neg				10.94	7703
M33453	alpha-ketoglutarate	Known	Krebs cycle	Energy	GC/MS				27.10	6,130
M01564	citrate	Known	Krebs cycle	Energy	GC/MS				8.01	7813
M01303	malate	Known	Krebs cycle	Energy	GC/MS				20.80	7,384
M37058	succinylcarnitine	Known	Krebs cycle	Energy	LC/MS pos				28.12	6948
M15488	acetylphosphate	Known	Oxidative phosphorylation	Energy	GC/MS				22.10	7,789
M11438	phosphate	Known	Oxidative phosphorylation	Energy	GC/MS				7.22	7789
M22842	cholate	Known	Bile acid metabolism	Lipid	LC/MS neg				28.90	5,929
M01114	deoxycholate	Known	Bile acid metabolism	Lipid	LC/MS neg				24.70	5194
M32346	glycochenodeoxycholate	Known	Bile acid metabolism	Lipid	LC/MS neg				12.10	7,087
M18476	glycocholate	Known	Bile acid metabolism	Lipid	LC/MS pos				12.37	5995
M18477	glycodeoxycholate	Known	Bile acid metabolism	Lipid	LC/MS neg				18.80	1,477
M39379	glycoursodeoxycholate	Known	Bile acid metabolism	Lipid	LC/MS Neg				7.02	4675
M27531	hyodeoxycholate	Known	Bile acid metabolism	Lipid	LC/MS neg				13.00	6,025
M18494	taurochenodeoxycholate	Known	Bile acid metabolism	Lipid	LC/MS neg				16.24	5716
M18497	taurocholate	Known	Bile acid metabolism	Lipid	LC/MS neg				23.30	4,032
M12261	taurodeoxycholate	Known	Bile acid metabolism	Lipid	LC/MS neg				21.24	1564
M36850	taurolithocholate 3-sulfate	Known	Bile acid metabolism	Lipid	LC/MS neg				23.80	6,904
M01605	ursodeoxycholate	Known	Bile acid metabolism	Lipid	LC/MS neg				13.53	5477
M35472	2-tetradecenoyl carnitine	Known	Carnitine metabolism	Lipid	LC/MS pos				25.20	6,998

© 2021 Wei Y et al. *JAMA Network Open.*

ID	Metabolite	Status	Pathway	Super-pathway	Platform	Compound ID	Quant. Mass.	Retention Time	CV (%)	Total N
M32654	3-dehydrocarnitine*	Known	Carnitine metabolism	Lipid	LC/MS pos				13.50	7809
M32198	acetylcarnitine	Known	Carnitine metabolism	Lipid	LC/MS pos				8.40	7,805
M15500	carnitine	Known	Carnitine metabolism	Lipid	LC/MS pos				5.22	7797
M38178	cis-4-decenoyl carnitine	Known	Carnitine metabolism	Lipid	LC/MS Pos				9.70	7,660
M33941	decanoylcarnitine	Known	Carnitine metabolism	Lipid	LC/MS pos				14.23	7766
M32328	hexanoylcarnitine	Known	Carnitine metabolism	Lipid	LC/MS pos				14.30	7,786
M34534	laurylcarnitine	Known	Carnitine metabolism	Lipid	LC/MS pos				27.87	5170
M33936	octanoylcarnitine	Known	Carnitine metabolism	Lipid	LC/MS pos				12.50	7,790
M35160	oleoylcarnitine	Known	Carnitine metabolism	Lipid	LC/MS pos				20.54	7708
M22189	palmitoylcarnitine	Known	Carnitine metabolism	Lipid	LC/MS pos				24.90	7,701
M34409	stearoylcarnitine	Known	Carnitine metabolism	Lipid	LC/MS pos				25.46	7183
M35718	dihomo-linolenate (20:3n3 or n6)	Known	Essential fatty acid	Lipid	LC/MS neg				10.30	7,805
M19323	docosahexaenoate (DHA; 22:6n3)	Known	Essential fatty acid	Lipid	LC/MS neg				11.47	7818
M32504	docosapentaenoate (n3 DPA; 22:5n3)	Known	Essential fatty acid	Lipid	LC/MS neg				14.10	7,821
M18467	eicosapentaenoate (EPA; 20:5n3)	Known	Essential fatty acid	Lipid	LC/MS neg				11.50	7816
M01105	linoleate (18:2n6)	Known	Essential fatty acid	Lipid	LC/MS neg				7.90	7,780
M34035	linolenate [alpha or gamma; (18:3n3 or 6)]	Known	Essential fatty acid	Lipid	LC/MS neg				9.28	7786
M34732	isovalerate	Known	Fatty acid metabolism	Lipid	LC/MS neg				14.10	7,080
M32412	butyrylcarnitine	Known	Fatty acid metabolism (also BCAA metabolism)	Lipid	LC/MS pos				13.67	7796
M32452	propionylcarnitine	Known	Fatty acid metabolism (also BCAA metabolism)	Lipid	LC/MS pos				9.40	7,813

© 2021 Wei Y et al. *JAMA Network Open.*

ID	Metabolite	Status	Pathway	Super-pathway	Platform	Compound ID	Quant. Mass.	Retention Time	CV (%)	Total N
M32455	linoleamide (18:2n6)	Known	Fatty acid, amide	Lipid	LC/MS pos				NA	1767
M32458	oleamide	Known	Fatty acid, amide	Lipid	LC/MS pos				83.40	1,765
M38768	15-methylpalmitate (isobar with 2-methylpalmitate)	Known	Fatty acid, branched	Lipid	LC/MS Neg				16.98	7371
M37253	2-hydroxyglutarate	Known	Fatty acid, dicarboxylate	Lipid	GC/MS				27.30	6,287
M31787	3-carboxy-4-methyl-5-propyl-2-furanpropanoate (CMPF)	Known	Fatty acid, dicarboxylate	Lipid	LC/MS neg				7.96	7812
M32388	dodecanedioate	Known	Fatty acid, dicarboxylate	Lipid	LC/MS neg				22.60	6,478
M35678	hexadecanedioate	Known	Fatty acid, dicarboxylate	Lipid	LC/MS neg				12.40	6887
M36754	octadecanedioate	Known	Fatty acid, dicarboxylate	Lipid	LC/MS neg				19.50	7,300
M32398	sebacate (decanedioate)	Known	Fatty acid, dicarboxylate	Lipid	LC/MS neg				NA	398
M35669	tetradecanedioate	Known	Fatty acid, dicarboxylate	Lipid	LC/MS neg				12.80	6,046
M36802	n-Butyl Oleate	Known	Fatty acid, ester	Lipid	GC/MS				28.89	4542
M35675	2-hydroxypalmitate	Known	Fatty acid, monohydroxy	Lipid	LC/MS neg				14.30	7,797
M17945	2-hydroxystearate	Known	Fatty acid, monohydroxy	Lipid	LC/MS neg				16.40	7763
M15506	choline	Known	Glycerolipid metabolism	Lipid	LC/MS pos				8.10	7,750
M15122	glycerol	Known	Glycerolipid metabolism	Lipid	GC/MS				8.23	7800
M15365	glycerol 3-phosphate (G3P)	Known	Glycerolipid metabolism	Lipid	GC/MS				14.50	7,781
M15990	glycerophosphorylcholine (GPC)	Known	Glycerolipid metabolism	Lipid	LC/MS pos				10.26	7156
M37112	chiro-inositol	Known	Inositol metabolism	Lipid	GC/MS				20.80	2,729
M01481	inositol 1-phosphate (I1P)	Known	Inositol metabolism	Lipid	GC/MS				NA	1379
M19934	myo-inositol	linoleat	Inositol metabolism	Lipid	GC/MS				15.30	7,803
M32379	scyllo-inositol	Known	Inositol metabolism	Lipid	GC/MS				33.87	6500
M00542	3-hydroxybutyrate (BHBA)	Known	Ketone bodies	Lipid	GC/MS				7.40	7,820

© 2021 Wei Y et al. *JAMA Network Open.*

ID	Metabolite	Status	Pathway	Super-pathway	Platform	Compound ID	Quant. Mass.	Retention Time	CV (%)	Total N
M33971	10-heptadecenoate (17:1n7)	Known	Long chain fatty acid	Lipid	LC/MS neg				11.55	7795
M33972	10-nonadecenoate (19:1n9)	Known	Long chain fatty acid	Lipid	LC/MS neg				12.60	7,789
M32980	adrenate (22:4n6)	Known	Long chain fatty acid	Lipid	LC/MS neg				23.17	7778
M01110	arachidonate (20:4n6)	Known	Long chain fatty acid	Lipid	LC/MS neg				9.10	7,816
M17805	dihomo-linoleate (20:2n6)	Known	Long chain fatty acid	Lipid	LC/MS neg				11.92	7800
M33587	eicosenoate (20:1n9 or 11)	Known	Long chain fatty acid	Lipid	LC/MS neg				12.10	7,799
M01121	margarate (17:0)	Known	Long chain fatty acid	Lipid	LC/MS neg				13.19	7796
M01365	myristate (14:0)	Known	Long chain fatty acid	Lipid	LC/MS neg				8.60	7,811
M32418	myristoleate (14:1n5)	Known	Long chain fatty acid	Lipid	LC/MS neg				8.42	7804
M01356	nonadecanoate (19:0)	Known	Long chain fatty acid	Lipid	LC/MS neg				17.10	7,783
M01359	oleate (18:1n9)	Known	Long chain fatty acid	Lipid	LC/MS neg				8.59	7768
M01336	palmitate (16:0)	Known	Long chain fatty acid	Lipid	LC/MS neg				9.00	7,800
M33447	palmitoleate (16:1n7)	Known	Long chain fatty acid	Lipid	LC/MS neg				8.90	7776
M01361	pentadecanoate (15:0)	Known	Long chain fatty acid	Lipid	GC/MS				24.10	7,502
M01358	stearate (18:0)	Known	Long chain fatty acid	Lipid	LC/MS neg				9.90	7803
M33969	stearidonate (18:4n3)	Known	Long chain fatty acid	Lipid	LC/MS neg				14.90	7,775
M33228	1-arachidonoylglycerophosphocholine*	Known	Lysolipid	Lipid	LC/MS pos				39.04	7507
M35186	1-arachidonoylglycerophosphoethanolamine*	Known	Lysolipid	Lipid	LC/MS neg				33.70	7,798
M34214	1-arachidonoylglycerophosphoinositol*	Known	Lysolipid	Lipid	LC/MS neg				22.38	7797
M33822	1-docosahexaenoylgl	Known	Lysolipid	Lipid	LC/MS pos				45.00	7,798

© 2021 Wei Y et al. *JAMA Network Open.*

ID	Metabolite	Status	Pathway	Super-pathway	Platform	Compound ID	Quant. Mass.	Retention Time	CV (%)	Total N
	ycerophosphocholine*									
M33871	1-eicosadienoylglycerophosphocholine*	Known	Lysolipid	Lipid	LC/MS pos				73.34	6892
M33821	1-eicosatrienoylglycerophosphocholine*	Known	Lysolipid	Lipid	LC/MS pos				39.90	7,809
M33957	1-heptadecanoylglycerophosphocholine	Known	Lysolipid	Lipid	LC/MS pos				83.43	7422
M34419	1-linoleoylglycerophosphocholine	Known	Lysolipid	Lipid	LC/MS pos				35.00	7,795
M32635	1-linoleoylglycerophosphoethanolamine*	Known	Lysolipid	Lipid	LC/MS neg				30.97	7817
M35626	1-myristoylglycerophosphocholine	Known	Lysolipid	Lipid	LC/MS pos				42.80	7,812
M33960	1-oleoylglycerophosphocholine	Known	Lysolipid	Lipid	LC/MS pos				39.88	7812
M35628	1-oleoylglycerophosphoethanolamine	Known	Lysolipid	Lipid	LC/MS neg				33.00	7,749
M33230	1-palmitoleoylglycerophosphocholine*	Known	Lysolipid	Lipid	LC/MS pos				41.93	7812
M33955	1-palmitoylglycerophosphocholine	Known	Lysolipid	Lipid	LC/MS pos				24.30	7,803
M35631	1-palmitoylglycerophosphoethanolamine	Known	Lysolipid	Lipid	LC/MS neg				28.76	7763
M35305	1-palmitoylglycerophosphoinositol*	Known	Lysolipid	Lipid	LC/MS neg				23.30	6,282
M39270	1-palmitoylplasmenylethanolamine*	Known	Lysolipid	Lipid	LC/MS Neg				31.63	4948
M33961	1-stearoylglycerophosphocholine	Known	Lysolipid	Lipid	LC/MS pos				84.90	7,817
M34416	1-stearoylglycerophosphoethanolamine	Known	Lysolipid	Lipid	LC/MS neg				38.48	7369

© 2021 Wei Y et al. *JAMA Network Open.*

ID	Metabolite	Status	Pathway	Super-pathway	Platform	Compound ID	Quant. Mass.	Retention Time	CV (%)	Total N
M19324	1-stearoylglycerophosphoinositol	Known	Lysolipid	Lipid	LC/MS neg				21.30	7,694
M35257	2-linoleoylglycerophosphocholine*	Known	Lysolipid	Lipid	LC/MS pos				45.91	6894
M36593	2-linoleoylglycerophosphoethanolamine*	Known	Lysolipid	Lipid	LC/MS neg				42.40	1,072
M35254	2-oleoylglycerophosphocholine*	Known	Lysolipid	Lipid	LC/MS pos				113.14	7524
M35253	2-palmitoylglycerophosphocholine*	Known	Lysolipid	Lipid	LC/MS pos				42.70	7,695
M35255	2-stearoylglycerophosphocholine*	Known	Lysolipid	Lipid	LC/MS pos				96.63	7730
M32497	10-undecenoate (11:1n1)	Known	Medium chain fatty acid	Lipid	LC/MS neg				15.90	7,806
M33968	5-dodecenoate (12:1n7)	Known	Medium chain fatty acid	Lipid	LC/MS neg				12.54	7770
M10642	caprate (10:0)	Known	Medium chain fatty acid	Lipid	LC/MS Neg				NA	7,799
M32489	caproate (6:0)	Known	Medium chain fatty acid	Lipid	LC/MS neg				11.93	7811
M32492	caprylate (8:0)	Known	Medium chain fatty acid	Lipid	LC/MS neg				9.60	7,802
M01644	heptanoate (7:0)	Known	Medium chain fatty acid	Lipid	LC/MS neg				11.27	7802
M01645	laurate (12:0)	Known	Medium chain fatty acid	Lipid	LC/MS neg				8.60	7,793
M12035	pelargonate (9:0)	Known	Medium chain fatty acid	Lipid	LC/MS neg				9.62	7803
M12067	undecanoate (11:0)	Known	Medium chain fatty acid	Lipid	LC/MS neg				15.50	7,500
M27447	1-linoleoylglycerol (1-monolinolein)	Known	Monoacylglycerol	Lipid	LC/MS neg				75.01	2797
M21184	1-oleoylglycerol (1-monoolein)	Known	Monoacylglycerol	Lipid	LC/MS pos				111.40	5,717
M21127	1-palmitoylglycerol (1-monopalmitin)	Known	Monoacylglycerol	Lipid	GC/MS				19.92	7429
M21188	1-stearoylglycerol (1-monostearin)	Known	Monoacylglycerol	Lipid	GC/MS				22.20	6,965

© 2021 Wei Y et al. *JAMA Network Open.*

ID	Metabolite	Status	Pathway	Super-pathway	Platform	Compound ID	Quant. Mass.	Retention Time	CV (%)	Total N
M33443	valerate	Known	Short chain fatty acid	Lipid	LC/MS neg				26.05	4102
M37506	palmitoyl sphingomyelin	Known	Sphingolipid	Lipid	GC/MS				12.00	7,814
M37202	4-androsten-3beta,17beta-diol disulfate 1*	Known	Sterol, Steroid	Lipid	LC/MS Neg				5.83	7804
M37203	4-androsten-3beta,17beta-diol disulfate 2*	Known	Sterol, Steroid	Lipid	LC/MS Neg				6.50	7,776
M37190	5alpha-androstan-3beta,17beta-diol disulfate	Known	Sterol, Steroid	Lipid	LC/MS Neg				7.45	7345
M37198	5alpha-pregnan-3beta,20alpha-diol disulfate	Known	Sterol, Steroid	Lipid	LC/MS Neg				9.30	4,229
M36776	7-alpha-hydroxy-3-oxo-4-cholestenoate (7-Hoca)	Known	Sterol, Steroid	Lipid	LC/MS neg				32.22	7784
M31591	androsterone sulfate	Known	Sterol, Steroid	Lipid	LC/MS neg				7.40	7,785
M00063	cholesterol	Known	Sterol, Steroid	Lipid	GC/MS				9.27	7813
M01712	cortisol	Known	Sterol, Steroid	Lipid	LC/MS pos				7.20	7,795
M01769	cortisone	Known	Sterol, Steroid	Lipid	LC/MS pos				15.26	7575
M32425	dehydroisoandrosterone sulfate (DHEA-S)	Known	Sterol, Steroid	Lipid	LC/MS neg				6.00	7,793
M33973	epiandrosterone sulfate	Known	Sterol, Steroid	Lipid	LC/MS neg				6.88	7769
M18474	estrone 3-sulfate	Known	Sterol, Steroid	Lipid	LC/MS neg				22.80	957
M33488	lathosterol	Known	Sterol, Steroid	Lipid	GC/MS				30.26	5457
M03127	hypoxanthine	Known	Purine metabolism, (hypo)xanthine, inosine containing	Nucleotide	LC/MS neg				29.00	7,287
M01123	inosine	Known	Purine metabolism, (hypo)xanthine, inosine containing	Nucleotide	LC/MS neg				NA	2675
M03147	xanthine	Known	Purine metabolism, (hypo)xanthine, inosine containing	Nucleotide	LC/MS pos				33.80	6,845
M00555	adenosine	Known	Purine metabolism, adenine containing	Nucleotide	LC/MS pos				NA	406

© 2021 Wei Y et al. *JAMA Network Open.*

ID	Metabolite	Status	Pathway	Super-pathway	Platform	Compound ID	Quant. Mass.	Retention Time	CV (%)	Total N
M15650	N1-methyladenosine	Known	Purine metabolism, adenine containing	Nucleotide	LC/MS pos				13.10	7,813
M35114	7-methylguanine	Known	Purine metabolism, guanine containing	Nucleotide	LC/MS pos				43.28	6091
M01573	guanosine	Known	Purine metabolism, guanine containing	Nucleotide	LC/MS pos				NA	2,343
M35137	N2,N2-dimethylguanosine	Known	Purine metabolism, guanine containing	Nucleotide	LC/MS pos				18.37	5228
M01107	allantoin	Known	Purine metabolism, urate metabolism	Nucleotide	GC/MS				27.90	5,705
M01604	urate	Known	Purine metabolism, urate metabolism	Nucleotide	LC/MS neg				5.15	7819
M33442	pseudouridine	Known	Pyrimidine metabolism, uracil containing	Nucleotide	LC/MS pos				12.60	7,785
M00606	uridine	Known	Pyrimidine metabolism, uracil containing	Nucleotide	LC/MS neg				6.26	7800
M22175	aspartylphenylalanine	Known	Dipeptide	Peptide	LC/MS pos				22.50	3,948
M37104	cyclo(leu-pro)	Known	Dipeptide	Peptide	LC/MS Pos				27.35	4833
M18357	glycylvaline	Known	Dipeptide	Peptide	LC/MS pos				NA	2,108
M36756	leucylleucine	Known	Dipeptide	Peptide	LC/MS pos				10.60	3386
M38150	phenylalanylphenylalanine	Known	Dipeptide	Peptide	LC/MS Pos				26.40	4,961
M35127	pro-hydroxy-pro	Known	Dipeptide	Peptide	LC/MS pos				18.62	7787
M31522	pyroglutamylglycine	Known	Dipeptide	Peptide	LC/MS neg				8.20	1,586
M33801	ADpSGEGDFXAEGGGVR*	Known	Fibrinogen cleavage peptide	Peptide	LC/MS pos				22.23	3939
M33084	ADSGEGDFXAEGGGVR*	Known	Fibrinogen cleavage peptide	Peptide	LC/MS pos				11.90	5,588
M31548	DSGEGDFXAEGGGVR*	Known	Fibrinogen cleavage peptide	Peptide	LC/MS pos				17.82	5371
M36738	gamma-glutamylglutamate	Known	gamma-glutamyl	Peptide	LC/MS pos				20.10	931
M02730	gamma-glutamylglutamine	Known	gamma-glutamyl	Peptide	LC/MS pos				29.73	7662
M34456	gamma-glutamylisoleucine*	Known	gamma-glutamyl	Peptide	LC/MS pos				12.80	5,522

© 2021 Wei Y et al. *JAMA Network Open.*

ID	Metabolite	Status	Pathway	Super-pathway	Platform	Compound ID	Quant. Mass.	Retention Time	CV (%)	Total N
M18369	gamma-glutamylleucine	Known	gamma-glutamyl	Peptide	LC/MS pos				9.25	7802
M33363	gamma-glutamylmethionine*	Known	gamma-glutamyl	Peptide	LC/MS pos				23.10	2,240
M33422	gamma-glutamylphenylalanine	Known	gamma-glutamyl	Peptide	LC/MS pos				13.29	7753
M33364	gamma-glutamylthreonine*	Known	gamma-glutamyl	Peptide	LC/MS pos				24.30	4,016
M02734	gamma-glutamyltyrosine	Known	gamma-glutamyl	Peptide	LC/MS pos				14.13	7468
M32393	gamma-glutamylvaline	Known	gamma-glutamyl	Peptide	LC/MS pos				9.30	7,753
M34420	bradykinin, des-arg(9)	Known	Polypeptide	Peptide	LC/MS pos				9.58	4570
M32836	HWESASXX*	Known	Polypeptide	Peptide	LC/MS pos				11.90	7,700
M18281	2-hydroxyhippurate (salicylurate)	Known	Benzoate metabolism	Xenobiotics	LC/MS neg				22.61	2592
M36848	3-ethylphenylsulfate*	Known	Benzoate metabolism	Xenobiotics	LC/MS neg				24.90	165
M36099	4-ethylphenylsulfate	Known	Benzoate metabolism	Xenobiotics	LC/MS neg				15.46	6012
M35527	4-hydroxyhippurate	Known	Benzoate metabolism	Xenobiotics	LC/MS Neg				20.90	4,368
M36098	4-vinylphenol sulfate	Known	Benzoate metabolism	Xenobiotics	LC/MS neg				6.75	7483
M15778	benzoate	Known	Benzoate metabolism	Xenobiotics	LC/MS neg				8.90	7,756
M35320	catechol sulfate	Known	Benzoate metabolism	Xenobiotics	LC/MS neg				5.77	7807
M15753	hippurate	Known	Benzoate metabolism	Xenobiotics	LC/MS pos				11.60	7,806
M27728	glycerol 2-phosphate	Known	Chemical	Xenobiotics	GC/MS				17.81	5912
M33173	2-hydroxyacetaminophen sulfate*	Known	Drug	Xenobiotics	LC/MS neg				14.20	1,688
M33178	2-methoxyacetaminophen sulfate*	Known	Drug	Xenobiotics	LC/MS neg				10.26	1086
M34365	3-(cystein-S-yl)acetaminophen*	Known	Drug	Xenobiotics	LC/MS pos				0.00	1,024

© 2021 Wei Y et al. *JAMA Network Open.*

ID	Metabolite	Status	Pathway	Super-pathway	Platform	Compound ID	Quant. Mass.	Retention Time	CV (%)	Total N
M12032	4-acetamidophenol	Known	Drug	Xenobiotics	GC/MS				0.00	756
M36097	4-acetaminophen sulfate	Known	Drug	Xenobiotics	LC/MS neg				8.60	498
M38658	atenolol	Known	Drug	Xenobiotics	LC/MS Pos				NA	182
M37033	carbamazepine*	Known	Drug	Xenobiotics	LC/MS pos				NA	24
M35322	hydroquinone sulfate	Known	Drug	Xenobiotics	LC/MS neg				7.19	451
M33085	hydroxypioglitazone*	Known	Drug	Xenobiotics	LC/MS pos				NA	8
M17799	ibuprofen	Known	Drug	Xenobiotics	LC/MS neg				11.23	1976
M18037	metoprolol	Known	Drug	Xenobiotics	LC/MS pos				NA	69
M34109	metoprolol acid metabolite*	Known	Drug	Xenobiotics	LC/MS pos				NA	286
M12122	naproxen	Known	Drug	Xenobiotics	LC/MS neg				NA	36
M33423	p-acetamidophenylglucuronide	Known	Drug	Xenobiotics	LC/MS pos				31.56	1494
M33139	pioglitazone*	Known	Drug	Xenobiotics	LC/MS pos				NA	8
M01515	salicylate	Known	Drug	Xenobiotics	GC/MS				27.34	2374
M33384	salicyluric glucuronide*	Known	Drug	Xenobiotics	LC/MS neg				0.00	832
M37459	ergothioneine	Known	Food component, Plant	Xenobiotics	LC/MS Pos				29.15	4453
M33009	homostachydrine*	Known	Food component, Plant	Xenobiotics	LC/MS pos				15.90	3,003
M31536	N-(2-furoyl)glycine	Known	Food component, Plant	Xenobiotics	LC/MS pos				23.19	604
M33935	piperine	Known	Food component, Plant	Xenobiotics	LC/MS pos				16.60	7,173
M18335	quinate	Known	Food component, Plant	Xenobiotics	GC/MS				23.80	6265
M21151	saccharin	Known	Food component, Plant	Xenobiotics	LC/MS neg				NA	2,449
M34384	stachydrine	Known	Food component, Plant	Xenobiotics	LC/MS pos				6.95	6745
M36095	thymol sulfate	Known	Food component, Plant	Xenobiotics	LC/MS neg				14.30	4,172

© 2021 Wei Y et al. *JAMA Network Open.*

ID	Metabolite	Status	Pathway	Super-pathway	Platform	Compound ID	Quant. Mass.	Retention Time	CV (%)	Total N
M20699	erythritol	Known	Sugar, sugar substitute, starch	Xenobiotics	GC/MS				12.86	7776
M00553	cotinine	Known	Tobacco metabolite	Xenobiotics	LC/MS pos				12.10	1,270
M34404	1,3,7-trimethylurate	Known	Xanthine metabolism	Xenobiotics	LC/MS neg				32.28	796
M34400	1,7-dimethylurate	Known	Xanthine metabolism	Xenobiotics	LC/MS neg				19.30	6,057
M34395	1-methylurate	Known	Xanthine metabolism	Xenobiotics	LC/MS pos				36.38	5520
M34389	1-methylxanthine	Known	Xanthine metabolism	Xenobiotics	LC/MS pos				30.60	5,504
M32445	3-methylxanthine	Known	Xanthine metabolism	Xenobiotics	LC/MS pos				34.89	5396
M34390	7-methylxanthine	Known	Xanthine metabolism	Xenobiotics	LC/MS pos				29.20	5,901
M00569	caffeine	Known	Xanthine metabolism	Xenobiotics	LC/MS pos				12.51	7719
M18254	paraxanthine	Known	Xanthine metabolism	Xenobiotics	LC/MS pos				14.40	7,504
M18392	theobromine	Known	Xanthine metabolism	Xenobiotics	LC/MS pos				8.61	7768
M18394	theophylline	Known	Xanthine metabolism	Xenobiotics	LC/MS neg				19.30	7,393
M32735	X-01911	Unknown			LC/MS Pos	32735	464.1	4.26	16.70	6360
M32587	X-02249	Unknown			LC/MS Neg	32587	267.200	4.030	6.50	7,818
M32549	X-02269	Unknown			LC/MS Neg	32549	255.1	1.55	21.62	7701
M12593	X-02973	Unknown			GC/MS				21.70	7,759
M12626	X-03003	Unknown			GC/MS				25.79	7689
M32709	X-03056--N-[3-(2-Oxopyrrolidin-1-yl)propyl]acetamide	Identified	Polyamine metabolism	Amino acid	LC/MS Pos				11.80	7,812
M12768	X-03088	Unknown			GC/MS				25.82	7474
M12770	X-03090	Unknown			GC/ms				47.50	1,394
M12774	X-03094	Unknown			GC/MS	12774	299	11.55	14.98	7804
M16634	X-04357	Unknown			GC/MS				32.30	7,502
M16816	X-04494	Unknown			GC/MS				27.07	5007
M16818	X-04495	Unknown			GC/MS				17.10	7,490

© 2021 Wei Y et al. *JAMA Network Open.*

ID	Metabolite	Status	Pathway	Super-pathway	Platform	Compound ID	Quant. Mass.	Retention Time	CV (%)	Total N
M16821	X-04498	Unknown			GC/MS				18.69	7160
M16822	X-04499--3,4-dihydroxybutyrate	Identified	Butanoate metabolism	Amino acid	GC/MS	16822	189.000	7.220	21.10	6,948
M16823	X-04500	Unknown			GC/MS				19.51	3647
M17059	X-04621	Unknown			GC/ms				0.00	84
M18283	X-05426	Unknown			GC/MS				22.38	6357
M18929	X-05907	Unknown			GC/MS				24.70	7,734
M32557	X-06126	Unknown			LC/MS Neg				6.71	7785
M19362	X-06226	Unknown			GC/MS				23.50	7,623
M19363	X-06227	Unknown			GC/ms				30.70	1690
M19364	X-06246	Unknown			GC/MS				25.00	7,358
M19368	X-06267	Unknown			GC/MS				29.03	7075
M19396	X-06307	Unknown			GC/MS				31.90	6,774
M19414	X-06350	Unknown			GC/MS				29.78	5161
M19415	X-06351	Unknown			GC/MS				24.20	4,791
M32560	X-07765	Unknown			LC/MS Neg				5.82	2170
M21630	X-08402	Unknown			GC/MS	21630	283.100	15.270	21.20	7,726
M22032	X-08766	Unknown			GC/MS				26.90	6050
M22481	X-08988	Unknown			GC/MS	22481	160.100	7.340	58.40	7,776
M22548	X-09026	Unknown			GC/MS				26.44	7627
M22649	X-09108	Unknown			GC/MS				28.30	6,910
M24074	X-09706	Unknown			GC/MS				22.41	7697
M32753	X-09789	Unknown			LC/MS Neg	32753	153.100	2.620	7.20	7,805
M33782	X-10346	Unknown			LC/MS Neg				17.76	4207
M25459	X-10395	Unknown			GC/MS	25459	156.000	9.940	18.60	7,784
M25599	X-10429	Unknown			GC/MS				24.60	6807
M27256	X-10500	Unknown			GC/MS				15.90	7,751
M27273	X-10506	Unknown			GC/MS				23.90	7710
M27278	X-10510	Unknown			GC/MS	27278	297.200	15.770	19.10	7,792
M28354	X-10675	Unknown			GC/ms				33.15	1241
M30805	X-10810	Unknown			GC/MS				31.50	7,232

© 2021 Wei Y et al. *JAMA Network Open.*

ID	Metabolite	Status	Pathway	Super-pathway	Platform	Compound ID	Quant. Mass.	Retention Time	CV (%)	Total N
M32518	X-11204	Unknown			LC/MS Pos				12.28	7799
M32564	X-11247	Unknown			LC/MS Neg				15.30	7,403
M32578	X-11261	Unknown			LC/MS Pos	32578	286.2	3.69	7.20	7771
M32616	X-11299	Unknown			LC/MS Neg				7.60	7,347
M32632	X-11315	Unknown			LC/MS Pos	32632	130.2	1.19	7.79	7785
M32634	X-11317	Unknown			LC/MS Neg				14.70	7,811
M32644	X-11327	Unknown			LC/MS Pos				15.99	7671
M32651	X-11334	Unknown			LC/MS Pos				18.00	5,462
M32689	X-11372	Unknown			LC/MS Neg				11.69	6043
M32691	X-11374	Unknown			LC/MS Pos				52.80	2,609
M32698	X-11381	Unknown			LC/MS Pos	32698	186.2	1.11	16.06	7753
M32729	X-11412	Unknown			LC/MS Pos				20.70	6,935
M32739	X-11422--xanthine	Identified	Purine metabolism, (hypo)xanthine/inosine containing	Nucleotide	LC/MS Neg				30.67	6346
M32740	X-11423--O-sulfo-L-tyrosine	Identified	Phenylalanine & tyrosine metabolism	Amino acid	LC/MS Neg				8.60	7,765
M32754	X-11437	Unknown			LC/MS Neg				7.21	6782
M32755	X-11438	Unknown			LC/MS Neg	32755	241.200	3.100	14.10	7,084
M32757	X-11440	Unknown			LC/MS Neg	32757	246.3	3.58	8.04	7686
M32758	X-11441	Unknown			LC/MS Neg	32758	331.100	3.780	10.90	7,072
M32759	X-11442	Unknown			LC/MS Neg	32759	331.1	3.91	9.03	7142
M32761	X-11444	Unknown			LC/MS Neg	32761	541.200	3.990	8.10	7,758
M32762	X-11445--5-alpha-pregnan-	Identified	Sterol/Steroid	Lipid	LC/MS Neg				10.28	2570

© 2021 Wei Y et al. *JAMA Network Open.*

ID	Metabolite	Status	Pathway	Super-pathway	Platform	Compound ID	Quant. Mass.	Retention Time	CV (%)	Total N
	3beta,20alpha-disulfate									
M32769	X-11452	Unknown			LC/MS Neg				10.80	6,519
M32786	X-11469	Unknown			LC/MS Pos	32786	239.1	3.82	38.22	7779
M32787	X-11470	Unknown			LC/MS Neg	32787	525.200	4.160	12.00	7,271
M32795	X-11478	Unknown			LC/MS Neg	32795	165.2	4.3	16.81	6593
M32800	X-11483	Unknown			LC/MS Neg				12.50	4,608
M32802	X-11485	Unknown			LC/MS Neg				20.22	4544
M32808	X-11491	Unknown			LC/MS Neg	32808	567.300	4.850	12.70	6,584
M32814	X-11497	Unknown			LC/MS Neg				25.72	7481
M32838	X-11521	Unknown			LC/MS Pos				14.60	7,193
M32846	X-11529	Unknown			LC/MS Neg	32846	624.3	4.85	8.90	6664
M32847	X-11530	Unknown			LC/MS Neg	32847	313.200	4.870	31.10	7,409
M32854	X-11537	Unknown			LC/MS Pos				31.56	2719
M32855	X-11538	Unknown			LC/MS Neg	32855	311.300	4.930	9.10	7,804
M32857	X-11540	Unknown			LC/MS Pos				35.23	2688
M32863	X-11546	Unknown			LC/MS Neg				10.30	2,015
M32867	X-11550	Unknown			LC/MS Neg	32867	250.2	5.15	14.65	7776
M32869	X-11552	Unknown			LC/MS Pos				40.70	2,170
M32910	X-11593--O-methylascorbate*	Identified	Ascorbate and aldarate metabolism	Cofactors and vitamins	LC/MS Neg	32910	189.2	0.79	11.62	7788
M33131	X-11786--methylcysteine	Identified	Cysteine, methionine, SAM, taurine metabolism	Amino acid	LC/MS Pos				27.30	2,811
M33132	X-11787	Unknown			LC/MS Pos	33132	148.1	1.13	9.53	7811

© 2021 Wei Y et al. *JAMA Network Open.*

ID	Metabolite	Status	Pathway	Super-pathway	Platform	Compound ID	Quant. Mass.	Retention Time	CV (%)	Total N
M33137	X-11792	Unknown			LC/MS Pos	33137	989.800	3.570	21.80	2,442
M33138	X-11793--oxidized bilirubin*	Identified	Hemoglobin and porphyrin metabolism	Cofactors and vitamins	LC/MS Pos	33138	601.1	3.57	20.18	7611
M33140	X-11795	Unknown			LC/MS Pos				25.60	7,763
M33144	X-11799	Unknown			LC/MS Pos	33144	226	1.58	16.95	5038
M33150	X-11805	Unknown			LC/MS Pos				NA	2,814
M33154	X-11809	Unknown			LC/MS Pos				27.76	1764
M33163	X-11818	Unknown			LC/MS Pos				16.60	6,790
M33165	X-11820	Unknown			LC/MS Pos	33165	948.3	4.68	33.16	7710
M33183	X-11838	Unknown			LC/MS Neg				34.00	1,793
M33188	X-11843	Unknown			LC/MS Neg				7.61	3938
M33190	X-11845	Unknown			LC/MS Neg				15.50	2,741
M33192	X-11847	Unknown			LC/MS Neg				28.40	6059
M33194	X-11849	Unknown			LC/MS Neg				13.10	5,350
M33195	X-11850	Unknown			LC/MS Neg				10.53	4868
M33197	X-11852	Unknown			LC/MS Neg				16.30	2,974
M33203	X-11858	Unknown			LC/MS Neg				18.42	3427
M33204	X-11859	Unknown			LC/MS Neg				11.30	2,721
M33221	X-11876	Unknown			LC/MS Neg				28.75	4965
M33225	X-11880	Unknown			LC/MS Neg				28.40	1,743
M33250	X-11905	Unknown			LC/MS Neg				23.50	4761
M33353	X-12007	Unknown			LC/MS Neg				16.00	1,362

© 2021 Wei Y et al. *JAMA Network Open.*

ID	Metabolite	Status	Pathway	Super-pathway	Platform	Compound ID	Quant. Mass.	Retention Time	CV (%)	Total N
M33359	X-12013	Unknown			LC/MS Neg				18.05	1948
M33380	X-12029	Unknown			LC/MS Neg				31.20	7,564
M33389	X-12038	Unknown			LC/MS Neg	33389	245.3	5.82	16.44	7758
M33390	X-12039	Unknown			LC/MS Neg				7.50	5,461
M33391	X-12040	Unknown			LC/MS Neg				16.54	559
M33408	X-12056	Unknown			LC/MS Pos				17.80	4,130
M33415	X-12063	Unknown			LC/MS Neg	33415	427.2	4.82	15.66	7197
M33507	X-12092	Unknown			LC/MS Pos	33507	144.200	1.130	15.30	7,500
M33508	X-12093	Unknown			LC/MS Pos	33508	203.2	1.05	28.43	2854
M33509	X-12094	Unknown			LC/MS Pos				17.20	7,467
M33510	X-12095--N1-methyl-3-pyridone-4-carboxamide	Identified	NAD metabolism	Nucleotide	LC/MS Pos	33510	153.1	1.6	13.11	7711
M33515	X-12100--hydroxytryptophan*	Identified	Tryptophan metabolism	Amino acid	LC/MS Pos	33515	221.100	1.760	20.00	7,499
M33531	X-12116	Unknown			LC/MS Pos				23.29	3039
M33609	X-12188	Unknown			LC/MS Neg				9.30	832
M33610	X-12189	Unknown			LC/MS Neg				6.75	481
M33616	X-12195	Unknown			LC/MS Neg				0.00	381
M33627	X-12206	Unknown			LC/MS Neg				22.99	2795
M33633	X-12212	Unknown			LC/MS Neg				17.60	4,566
M33637	X-12216	Unknown			LC/MS Neg				17.98	5369
M33638	X-12217	Unknown			LC/MS Neg				12.40	6,469
M33652	X-12230	Unknown			LC/MS Neg				13.13	5649

© 2021 Wei Y et al. *JAMA Network Open.*

ID	Metabolite	Status	Pathway	Super-pathway	Platform	Compound ID	Quant. Mass.	Retention Time	CV (%)	Total N
M33653	X-12231	Unknown			LC/MS Neg				15.40	5,111
M33658	X-12236	Unknown			LC/MS Neg				NA	1924
M33666	X-12244--N-acetylcarnosine	Identified	Dipeptide	Peptide	LC/MS Pos				14.40	6,608
M33675	X-12253	Unknown			LC/MS Neg				8.98	6004
M33683	X-12261	Unknown			LC/MS Neg				14.60	1,050
M33685	X-12263	Unknown			LC/MS Neg				18.19	247
M33751	X-12329	Unknown			LC/MS Neg				31.30	867
M33833	X-12405	Unknown			LC/MS Neg				17.52	6676
M33835	X-12407	Unknown			LC/MS Neg				18.60	2,814
M33864	X-12428	Unknown			LC/MS Neg				16.74	1439
M33877	X-12435	Unknown			LC/MS Neg				NA	231
M33883	X-12441--12-hydroxyeicosatetraenoate (12-HETE)	Identified	Eicosanoid	Lipid	LC/MS Neg				27.42	2761
M33884	X-12442--5,8-tetradecadienoate	Identified	Long chain fatty acid	Lipid	LC/MS Neg	33884	223.400	5.280	14.80	7,780
M33885	X-12443	Unknown			LC/MS Neg				132.93	5357
M33892	X-12450	Unknown			LC/MS Neg				25.60	6,432
M33901	X-12456	Unknown			LC/MS Neg	33901	427.2	4.34	18.29	4774
M33910	X-12465	Unknown			LC/MS Pos				13.80	5,883
M34040	X-12510--2-aminooctanoic acid	Identified	Amino fatty acid	Amino acid	LC/MS Pos				16.82	7566
M34062	X-12524	Unknown			LC/MS Neg				13.00	7,809
M34102	X-12539	Unknown			LC/MS Neg				29.53	1000
M34112	X-12544	Unknown			LC/MS Pos				9.50	5,893

© 2021 Wei Y et al. *JAMA Network Open.*

ID	Metabolite	Status	Pathway	Super-pathway	Platform	Compound ID	Quant. Mass.	Retention Time	CV (%)	Total N
M34123	X-12556	Unknown			GC/MS	34123	116.9	6.61	20.76	7483
M34221	X-12627	Unknown			LC/MS Neg	34221	357.400	5.840	27.90	7,419
M34244	X-12644	Unknown			LC/MS Neg				41.19	7795
M34245	X-12645	Unknown			LC/MS Neg				64.10	5,254
M34289	X-12680	Unknown			LC/MS Pos				22.01	2311
M34306	X-12696	Unknown			LC/MS Neg				12.40	7,409
M34314	X-12704	Unknown			LC/MS Neg				0.00	1813
M34321	X-12711	Unknown			LC/MS Neg				14.70	393
M34322	X-12712	Unknown			LC/MS Neg				0.00	260
M34327	X-12717	Unknown			LC/MS Neg				NA	1,632
M34329	X-12719	Unknown			LC/MS Neg				NA	1539
M34336	X-12726	Unknown			LC/MS Neg				29.00	5,495
M34338	X-12728	Unknown			LC/MS Neg				12.05	537
M34339	X-12729	Unknown			LC/MS Neg				17.00	1,753
M34344	X-12734	Unknown			LC/MS Neg				21.64	5616
M34350	X-12740	Unknown			LC/MS Neg				12.40	3,731
M34359	X-12749	Unknown			LC/MS Pos	34359	262.1	1.51	13.16	7178
M34369	X-12759	Unknown			LC/MS Pos				NA	63
M34441	X-12771	Unknown			GC/MS				35.16	4734
M34453	X-12776	Unknown			LC/MS Neg				13.90	7,764
M34469	X-12786	Unknown			GC/MS				28.89	6566
M34481	X-12798	Unknown			LC/MS Pos	34481	240.100	1.840	17.80	7,552

© 2021 Wei Y et al. *JAMA Network Open.*

ID	Metabolite	Status	Pathway	Super-pathway	Platform	Compound ID	Quant. Mass.	Retention Time	CV (%)	Total N
M34499	X-12816	Unknown			LC/MS Neg				13.10	4524
M34513	X-12830	Unknown			LC/MS Neg				15.70	3,415
M34516	X-12833	Unknown			LC/MS Neg				25.26	243
M34527	X-12844	Unknown			LC/MS Neg	34527	539.300	4.120	9.30	7,768
M34530	X-12847	Unknown			LC/MS Neg				15.86	4036
M34533	X-12850	Unknown			LC/MS Neg	34533	263.800	4.820	11.10	6,251
M34535	X-12851	Unknown			LC/MS Neg				11.09	4808
M34539	X-12855	Unknown			LC/MS Pos				25.70	5,120
M34674	X-12990--docosapentaenoic acid (n6-DPA)	Identified	Essential fatty acid	Lipid	LC/MS Neg				13.67	2581
M34761	X-13069	Unknown			LC/MS Neg				21.00	6,770
M34826	X-13134	Unknown			LC/MS Neg				36.71	975
M34878	X-13183--stearamide	Identified	Fatty acid amide	Lipid	LC/MS Pos				48.50	2,503
M34912	X-13215	Unknown			LC/MS Neg	34912	149.2	5.2	18.56	6305
M35072	X-13372	Unknown			LC/MS Neg				18.40	1,500
M35187	X-13429	Unknown			LC/MS Neg	35187	263.7	4.84	13.51	6344
M35189	X-13431--nonanoylcarnitine*	Identified	Carnitine metabolism	Lipid	LC/MS Pos	35189	302.300	4.180	25.80	6,591
M35193	X-13435	Unknown			LC/MS Pos	35193	314.3	4.61	15.21	6970
M35240	X-13477	Unknown			LC/MS Pos	35240	176.200	0.850	21.10	5,952
M35270	X-13496	Unknown			GC/MS				17.73	7656
M35326	X-13548	Unknown			LC/MS Pos	35326	295.100	2.330	19.90	6,022
M35327	X-13549	Unknown			LC/MS Pos				18.78	7330

© 2021 Wei Y et al. *JAMA Network Open.*

ID	Metabolite	Status	Pathway	Super-pathway	Platform	Compound ID	Quant. Mass.	Retention Time	CV (%)	Total N
M35331	X-13553	Unknown			LC/MS Neg				15.10	3,085
M35397	X-13619	Unknown			GC/MS				20.87	7791
M35422	X-13640	Unknown			LC/MS Neg				0.00	660
M35451	X-13658	Unknown			LC/MS Neg				23.08	1412
M35464	X-13671	Unknown			LC/MS Neg	35464	315.300	5.290	13.20	7,311
M35508	X-13699	Unknown			LC/MS Pos				11.86	146
M35551	X-13741	Unknown			LC/MS Neg				10.10	5,325
M35754	X-13859	Unknown			LC/MS Neg				31.24	7002
M35977	X-14056	Unknown			LC/MS Pos				20.90	7,259
M35978	X-14057	Unknown			LC/MS Pos				33.14	4716
M36009	X-14086	Unknown			LC/MS Pos				NA	2,686
M36115	X-14189--leucylalanine	Identified	Dipeptide	Peptide	LC/MS Pos				8.28	2745
M36131	X-14205--alpha-glutamyltyrosine	Identified	Dipeptide	Peptide	LC/MS Pos				13.50	1,789
M36134	X-14208--phenylalanylserine	Identified	Dipeptide	Peptide	LC/MS Pos				20.68	2455
M36230	X-14304--leucylalanine	Identified	Dipeptide	Peptide	LC/MS Neg				13.20	2,434
M36300	X-14374	Unknown			LC/MS Pos				20.61	7627
M36376	X-14450--phenylalanylleucine	Identified	Dipeptide	Peptide	LC/MS Pos				16.00	2,555
M36394	X-14468	Unknown			LC/MS Pos				NA	368
M36399	X-14473	Unknown			LC/MS Pos	36399	211.200	3.270	24.20	6,884
M36468	X-14541	Unknown			LC/MS Neg				NA	1941
M36515	X-14588	Unknown			LC/MS Neg				9.20	7,777

© 2021 Wei Y et al. *JAMA Network Open.*

ID	Metabolite	Status	Pathway	Super-pathway	Platform	Compound ID	Quant. Mass.	Retention Time	CV (%)	Total N
M36552	X-14625	Unknown			LC/MS Neg	36552	308.1	0.72	14.88	7511
M36553	X-14626	Unknown			LC/MS Neg	36553	288.800	4.800	26.20	6,904
M36559	X-14632	Unknown			LC/MS Neg				20.47	1703
M36585	X-14658	Unknown			LC/MS Neg				16.90	3,533
M36589	X-14662	Unknown			LC/MS Neg				13.70	4212
M36590	X-14663	Unknown			LC/MS Neg				26.90	319
M36673	X-14745	Unknown			GC/MS				43.55	5602
M37004	X-14977--vanillin	Identified	Food component/Plant	Xenobiotics	LC/MS Pos				22.10	1,789
M17807	X-18601	Unknown			LC/MS neg				7.91	7663

© 2021 Wei Y et al. *JAMA Network Open.*

eTable 3. Association Results Derived From mQTL Analysis of SNPs on Mannose With P Value<5×10^{-8}

Chr	Position (hg19)	SNP	Major allele	Minor allele	MAF	Coefficient	Standard Error	*P*
2	27544285	rs1122227	T	C	0.2050	0.0148	0.0026	8.81E-09
2	27873326	rs8731	C	G	0.7955	-0.0169	0.0026	4.02E-11
2	27504312	rs1992291	A	G	0.7845	-0.0153	0.0026	2.03E-09
2	27825715	rs12467476	T	G	0.7355	0.0336	0.0025	1.03E-42
2	28418189	rs13000936	A	G	0.3138	0.0135	0.0024	3.27E-08
2	28225861	rs11692105	T	G	0.2450	0.0141	0.0025	1.74E-08
2	28199424	rs12465000	A	G	0.7840	-0.0152	0.0026	2.85E-09
2	28344285	rs4401177	A	G	0.1204	-0.0201	0.0036	2.56E-08
2	27853245	rs13002853	C	G	0.7434	0.0327	0.0025	1.69E-40
2	27839539	rs2068834	T	C	0.7229	0.0324	0.0024	2.29E-40
2	27688601	rs1647276	T	C	0.3894	0.0248	0.0024	6.91E-25
2	27508710	rs11686131	T	C	0.2051	0.0148	0.0026	7.45E-09
2	27579231	rs6760828	T	C	0.6099	-0.0257	0.0024	5.09E-27
2	27743154	rs780092	A	G	0.8479	-0.0226	0.0032	3.54E-12
2	27519153	rs13404327	T	G	0.2530	0.0193	0.0025	1.09E-14
2	27961344	rs1317580	C	G	0.2568	-0.0167	0.0025	2.91E-11
2	28088580	rs4666028	A	T	0.1739	0.0152	0.0026	5.99E-09
2	27811722	rs12478841	A	G	0.7347	0.0336	0.0025	9.05E-43
2	27528692	rs4665963	T	C	0.7462	-0.0194	0.0025	9.29E-15
2	27767107	rs1972669	T	G	0.5182	0.0132	0.0024	3.61E-08
2	28107692	rs6708889	T	C	0.7928	-0.0152	0.0026	2.60E-09
2	27644464	rs1728922	A	C	0.6098	-0.0253	0.0024	4.32E-26
2	27995931	rs3792252	A	G	0.7527	-0.0176	0.0025	2.11E-12
2	27722416	rs1260320	A	G	0.3855	0.0249	0.0024	2.45E-25
2	27855397	rs10173720	A	T	0.7974	-0.0181	0.0025	1.05E-12
2	27550967	rs1049817	A	G	0.6106	-0.0251	0.0024	9.82E-26
2	27750546	rs2911711	A	T	0.5511	0.0363	0.0024	2.37E-53

© 2021 Wei Y et al. *JAMA Network Open.*

Chr	Position (hg19)	SNP	Major allele	Minor allele	MAF	Coefficient	Standard Error	*P*
2	27933642	rs4616435	T	C	0.2107	-0.0291	0.0025	8.41E-31
2	27715207	rs8395	A	T	0.3838	0.0256	0.0024	2.91E-26
2	28200152	rs2337374	A	G	0.7840	-0.0152	0.0026	2.84E-09
2	28367761	rs6751559	T	G	0.8993	0.0183	0.0034	4.96E-08
2	28301540	rs937813	T	C	0.9025	0.0271	0.0039	2.65E-12
2	27801418	rs1919126	A	C	0.4816	-0.0132	0.0024	3.48E-08
2	28281545	rs6547820	A	G	0.7939	-0.0150	0.0026	4.43E-09
2	28523834	rs12617913	T	C	0.5964	-0.0131	0.0024	4.68E-08
2	28232279	rs2839791	A	C	0.2158	0.0150	0.0026	4.01E-09
2	27839832	rs7571558	C	G	0.7974	-0.0200	0.0026	5.09E-15
2	27789861	rs965813	A	T	0.2018	0.0192	0.0026	5.14E-14
2	28396483	rs7586645	A	G	0.3229	0.0134	0.0024	4.02E-08
2	27910706	rs13023094	A	C	0.7918	0.0298	0.0025	2.74E-32
2	27995781	rs3736594	A	C	0.2560	0.0179	0.0025	9.02E-13
2	28090059	rs10171517	T	C	0.2015	0.0174	0.0025	7.85E-12
2	28079344	rs4233719	A	C	0.2159	0.0153	0.0025	2.06E-09
2	27802805	rs3811644	A	G	0.7989	-0.0192	0.0026	5.28E-14
2	27741237	rs780094	T	C	0.3977	-0.0428	0.0024	6.89E-73
2	27952406	rs12994085	T	C	0.7705	-0.0180	0.0025	1.07E-12
2	27840640	rs4666002	C	G	0.2757	-0.0386	0.0042	9.98E-20
2	27801759	rs1919128	A	G	0.7347	0.0336	0.0024	8.39E-43
2	27574953	rs4665969	T	C	0.6105	-0.0256	0.0024	1.01E-26
2	27706640	rs2272417	T	C	0.5930	-0.0224	0.0024	2.75E-20
2	27663416	rs1260342	T	G	0.3894	0.0248	0.0024	6.93E-25
2	27488432	rs6547521	C	G	0.2826	0.0167	0.0025	1.32E-11
2	27846645	rs1528403	C	G	0.5290	0.0138	0.0024	1.19E-08
2	27895073	rs2178198	T	C	0.1351	-0.0254	0.0033	1.98E-14
2	28082494	rs4578809	A	G	0.1739	0.0152	0.0026	5.99E-09
2	27426878	rs1275522	T	C	0.7113	-0.0167	0.0025	1.29E-11
2	27721971	rs813592	T	C	0.6118	-0.0254	0.0024	3.51E-26

© 2021 Wei Y et al. *JAMA Network Open.*

Chr	Position (hg19)	SNP	Major allele	Minor allele	MAF	Coefficient	Standard Error	*P*
2	28256831	rs9309658	A	G	0.2157	0.0150	0.0026	4.26E-09
2	27637235	rs2010087	T	C	0.3855	0.0255	0.0024	3.01E-26
2	27613617	rs11891554	A	G	0.0427	0.0337	0.0055	1.18E-09
2	27424636	rs1395	A	G	0.7024	-0.0170	0.0025	5.97E-12
2	27455726	rs13399758	T	C	0.9349	-0.0275	0.0049	1.47E-08
2	27449293	rs7593448	C	G	0.7116	-0.0169	0.0025	7.32E-12
2	27731212	rs3817588	T	C	0.8135	-0.0239	0.0026	1.08E-19
2	28418031	rs6712705	T	C	0.6814	-0.0135	0.0024	3.31E-08
2	27783801	rs4665382	T	C	0.7353	0.0336	0.0024	7.91E-43
2	27393030	rs1659689	A	G	0.2752	0.0166	0.0025	1.92E-11
2	27958855	rs6753736	C	G	0.2214	0.0193	0.0025	2.78E-14
2	27975394	rs6547796	T	C	0.2557	0.0178	0.0025	1.00E-12
2	28520083	rs10189899	A	G	0.4003	0.0137	0.0024	1.15E-08
2	27659491	rs780102	T	C	0.6099	-0.0248	0.0024	6.94E-25
2	28162861	rs4390729	T	C	0.2161	0.0152	0.0026	2.89E-09
2	27746832	rs8179252	A	C	0.6073	-0.0252	0.0024	1.26E-25
2	27604279	rs4582	A	G	0.6103	-0.0253	0.0024	4.81E-26
2	28162274	rs13416434	T	C	0.2161	0.0152	0.0026	2.89E-09
2	27742603	rs780093	T	C	0.3985	-0.0433	0.0024	1.48E-75
2	27703495	rs1260345	A	G	0.5881	-0.0223	0.0024	3.53E-20
2	27611469	rs7594812	A	G	0.6097	-0.0253	0.0024	4.63E-26
2	27452784	rs6717980	T	C	0.7122	-0.0169	0.0025	7.43E-12
2	27519254	rs13404446	A	G	0.2530	0.0194	0.0025	8.83E-15
2	27841305	rs6756238	A	G	0.4721	-0.0133	0.0024	2.77E-08
2	27852637	rs6547738	A	G	0.5281	0.0132	0.0024	2.89E-08
2	28530720	rs4666052	A	G	0.4003	0.0137	0.0024	1.17E-08
2	28113911	rs2305929	A	G	0.8169	0.0260	0.0025	2.05E-24
2	27443196	rs4665958	A	G	0.7125	-0.0169	0.0025	7.01E-12
2	27987046	rs12477908	A	C	0.2557	0.0178	0.0025	9.97E-13
2	28443050	rs7349418	T	C	0.5484	0.0154	0.0024	1.85E-10

© 2021 Wei Y et al. *JAMA Network Open.*

Chr	Position (hg19)	SNP	Major allele	Minor allele	MAF	Coefficient	Standard Error	*P*
2	27652153	rs780100	T	G	0.3894	0.0248	0.0024	6.99E-25
2	28144067	rs1870325	A	G	0.7922	-0.0152	0.0026	2.84E-09
2	27471025	rs1561535	T	C	0.2818	0.0163	0.0025	4.13E-11
2	28255302	rs937812	T	C	0.7843	-0.0150	0.0026	4.26E-09
2	27851918	rs3749147	A	G	0.2755	-0.0363	0.0043	1.72E-17
2	27932587	rs6727388	A	G	0.7893	0.0291	0.0025	8.35E-31
2	27801493	rs1919127	T	C	0.7340	0.0336	0.0024	8.37E-43
2	28213424	rs10175508	T	C	0.7840	-0.0144	0.0026	1.69E-08
2	27748624	rs1260333	A	G	0.4501	-0.0363	0.0024	2.37E-53
2	28018293	rs4666012	A	G	0.8018	-0.0152	0.0026	3.43E-09
2	28258027	rs7593127	A	G	0.7843	-0.0150	0.0026	4.27E-09
2	28517861	rs4666051	A	G	0.4003	0.0137	0.0024	1.16E-08
2	28140349	rs2337372	A	G	0.1736	0.0144	0.0026	3.96E-08
2	28219941	rs1458398	A	G	0.2159	0.0151	0.0026	3.40E-09
2	27968454	rs9967838	A	C	0.2494	0.0178	0.0025	1.01E-12
2	27818721	rs1919129	C	G	0.7974	-0.0200	0.0026	5.11E-15
2	27536380	rs4665965	T	C	0.7468	-0.0184	0.0025	2.41E-13
2	27693485	rs1647266	T	C	0.6106	-0.0248	0.0024	6.91E-25
2	27450724	rs3769143	A	G	0.2889	0.0170	0.0025	5.12E-12
2	27648726	rs4665978	T	C	0.5593	-0.0311	0.0024	1.28E-39
2	27432547	rs1275530	A	G	0.2878	0.0170	0.0025	4.62E-12
2	27755825	rs4665987	A	G	0.2653	-0.0336	0.0024	8.06E-43
2	27441397	rs1275538	A	G	0.2574	0.0153	0.0025	6.67E-10
2	28094270	rs13016573	C	G	0.8258	-0.0154	0.0026	2.84E-09
2	27430781	rs1275528	A	G	0.2878	0.0169	0.0025	6.79E-12
2	27812252	rs6760250	A	G	0.2653	-0.0336	0.0025	9.09E-43
2	27567407	rs6743819	T	G	0.3901	0.0257	0.0024	5.25E-27
2	27791555	rs4665383	C	G	0.7335	0.0334	0.0025	1.63E-41
2	27600239	rs13472	A	G	0.3844	0.0261	0.0024	1.93E-27
2	27972833	rs12104449	A	G	0.8737	0.0253	0.0033	3.24E-14

© 2021 Wei Y et al. *JAMA Network Open.*

Chr	Position (hg19)	SNP	Major allele	Minor allele	MAF	Coefficient	Standard Error	*P*
2	27398196	rs2580754	A	T	0.7098	-0.0166	0.0025	1.45E-11
2	27566520	rs11689803	A	T	0.2661	0.0251	0.0025	6.04E-24
2	27860258	rs2141371	A	G	0.6820	-0.0198	0.0024	3.27E-16
2	28202936	rs898032	C	G	0.2160	0.0152	0.0026	2.84E-09
2	28124171	rs10177845	A	G	0.7839	-0.0152	0.0025	2.60E-09
2	27416702	rs1275504	A	T	0.2976	0.0168	0.0025	9.83E-12
2	28101938	rs6706209	T	C	0.7857	-0.0148	0.0025	6.05E-09
2	27589810	rs2280737	T	C	0.6215	-0.0255	0.0024	1.67E-25
2	27728777	rs2293572	C	G	0.6148	-0.0264	0.0024	1.17E-27
2	27635582	rs1060525	A	G	0.6112	-0.0257	0.0024	5.82E-27
2	28529165	rs10165668	A	G	0.5997	-0.0137	0.0024	1.16E-08
2	28085311	rs7595986	A	G	0.7834	-0.0153	0.0025	2.06E-09
2	27839369	rs4666000	T	C	0.7221	0.0323	0.0024	4.85E-40
2	28276643	rs1395324	C	G	0.2157	0.0150	0.0026	4.29E-09
2	27389195	rs1275541	A	G	0.2833	0.0166	0.0025	1.92E-11
2	27743215	rs814295	A	G	0.8452	-0.0217	0.0032	1.94E-11
2	27830067	rs6719960	A	G	0.7982	-0.0201	0.0026	3.16E-15
2	27848397	rs4665384	A	G	0.5280	0.0133	0.0024	2.66E-08
2	28085347	rs13021285	T	C	0.8261	-0.0152	0.0026	5.99E-09
2	28019175	rs4666014	A	G	0.2072	0.0146	0.0026	1.33E-08
2	27778091	rs6706610	A	G	0.4818	-0.0132	0.0024	3.51E-08
2	28168415	rs7577437	A	G	0.2161	0.0152	0.0026	2.89E-09
2	28087205	rs13004096	T	C	0.1732	0.0152	0.0026	5.99E-09
2	28080214	rs13025314	A	G	0.1739	0.0152	0.0026	5.99E-09
2	27685388	rs780110	A	G	0.4424	0.0306	0.0024	4.06E-38
2	28081630	rs4578808	T	G	0.1739	0.0152	0.0026	5.99E-09
2	27420690	rs1659685	T	C	0.7025	-0.0165	0.0025	2.25E-11
2	27542041	rs1975384	T	C	0.2051	0.0148	0.0026	8.80E-09
2	28086765	rs11902158	A	G	0.8261	-0.0152	0.0026	5.99E-09
2	27657167	rs704791	T	C	0.6106	-0.0248	0.0024	6.94E-25

© 2021 Wei Y et al. *JAMA Network Open.*

Chr	Position (hg19)	SNP	Major allele	Minor allele	MAF	Coefficient	Standard Error	*P*
2	28271435	rs13402420	T	G	0.2150	0.0150	0.0026	4.29E-09
2	27515105	rs4665962	A	G	0.7949	-0.0148	0.0026	7.43E-09
2	27847606	rs6749426	T	C	0.5280	0.0133	0.0024	2.70E-08
2	27981655	rs12471347	C	G	0.2455	0.0170	0.0025	1.10E-11
2	28199736	rs12465103	C	G	0.7840	-0.0152	0.0026	2.85E-09
2	27677691	rs780104	A	G	0.3894	0.0248	0.0024	6.92E-25
2	27830990	rs6547734	T	G	0.4808	-0.0131	0.0024	3.87E-08
2	27718474	rs780090	T	C	0.0854	-0.0282	0.0041	8.28E-12
2	28209283	rs10209126	T	C	0.7840	-0.0152	0.0026	2.96E-09
2	28023120	rs11127125	T	C	0.7947	-0.0146	0.0026	1.38E-08
2	28098724	rs13411592	A	G	0.7844	-0.0152	0.0025	2.29E-09
2	28118662	rs7607546	A	G	0.8264	-0.0151	0.0026	7.21E-09
2	27934731	rs6727215	A	G	0.2100	-0.0291	0.0025	8.53E-31
2	28090820	rs898034	T	C	0.2015	0.0174	0.0025	7.85E-12
2	27980944	rs10209020	T	C	0.7443	-0.0178	0.0025	9.99E-13
2	27774702	rs2384628	T	G	0.5304	0.0141	0.0024	4.03E-09
2	27684734	rs780107	A	G	0.6106	-0.0248	0.0024	6.92E-25
2	27558252	rs11684134	A	G	0.5674	-0.0250	0.0024	1.04E-25
2	27568565	rs10205219	T	C	0.6099	-0.0257	0.0024	5.22E-27
2	28020966	rs3935148	A	T	0.1966	0.0152	0.0026	3.64E-09
2	27796526	rs10165098	T	C	0.4810	-0.0132	0.0024	3.43E-08
2	28136351	rs1458396	A	G	0.2160	0.0152	0.0025	2.68E-09
2	27460968	rs1141313	A	G	0.7122	-0.0169	0.0025	7.46E-12
2	27730817	rs8179219	A	G	0.0381	0.0343	0.0062	2.76E-08
2	27712408	rs7583698	T	C	0.0483	0.0338	0.0049	8.55E-12
2	27613031	rs12476704	A	C	0.3843	0.0261	0.0024	1.79E-27
2	27951658	rs867282	T	C	0.2214	0.0167	0.0025	3.43E-11
2	27715416	rs2303369	T	C	0.3838	0.0256	0.0024	2.91E-26
2	28189280	rs1902966	A	G	0.7839	-0.0152	0.0026	2.87E-09
2	27892023	rs2272406	A	T	0.7912	0.0293	0.0025	2.19E-31

© 2021 Wei Y et al. *JAMA Network Open.*

Chr	Position (hg19)	SNP	Major allele	Minor allele	MAF	Coefficient	Standard Error	*P*
2	28155195	rs6547815	A	T	0.7924	-0.0152	0.0026	3.28E-09
2	27698343	rs780117	C	G	0.6106	-0.0248	0.0024	6.90E-25
2	27988821	rs13013484	A	G	0.7379	-0.0186	0.0025	9.33E-14
2	27772879	rs6749052	A	T	0.5182	0.0132	0.0024	3.56E-08
2	28268467	rs6738887	T	C	0.7846	-0.0149	0.0025	4.61E-09
2	27631191	rs7563162	T	C	0.3896	0.0247	0.0024	4.15E-24
2	27681598	rs780106	A	C	0.6106	-0.0248	0.0024	6.92E-25
2	27646770	rs6547626	T	C	0.3902	0.0253	0.0024	4.32E-26
2	28394293	rs17759746	T	C	0.9007	0.0184	0.0034	4.96E-08
2	28235184	rs7602328	A	G	0.7842	-0.0150	0.0026	4.09E-09
2	27663215	rs1260341	A	T	0.3894	0.0248	0.0024	6.93E-25
2	27441302	rs1275537	C	G	0.2766	0.0157	0.0025	1.93E-10
2	27743423	rs11681351	A	G	0.3941	0.0259	0.0024	5.45E-27
2	28261163	rs6731650	A	C	0.7843	-0.0150	0.0026	4.27E-09
2	27608115	rs1647284	T	C	0.3896	0.0253	0.0024	4.71E-26
2	27808154	rs6734059	T	C	0.7991	-0.0189	0.0025	1.18E-13
2	27595756	rs1528533	C	G	0.3897	0.0253	0.0024	5.15E-26
2	28143017	rs10173848	T	G	0.7975	-0.0155	0.0026	1.89E-09
2	28003174	rs13030345	T	G	0.1794	-0.0257	0.0025	6.33E-24
2	28029649	rs4665392	A	G	0.7946	-0.0146	0.0026	1.35E-08
2	27640325	rs4665976	A	G	0.6098	-0.0253	0.0024	4.33E-26
2	27844601	rs1881396	T	G	0.7975	-0.0187	0.0025	1.71E-13
2	27725761	rs715326	T	C	0.3789	0.0313	0.0041	1.78E-14
2	27667297	rs4803	A	G	0.6106	-0.0254	0.0024	4.15E-26
2	27462405	rs2014252	A	T	0.2878	0.0169	0.0025	7.46E-12
2	28079775	rs4233720	A	G	0.8261	-0.0152	0.0026	5.99E-09
2	27851120	rs12615690	T	C	0.5272	0.0133	0.0024	2.51E-08
2	27642734	rs11675428	A	C	0.8181	-0.0235	0.0026	2.23E-19
2	27584666	rs7586601	A	G	0.5621	-0.0311	0.0024	2.29E-39
2	27796927	rs6704596	A	G	0.7982	-0.0192	0.0026	5.26E-14

© 2021 Wei Y et al. *JAMA Network Open.*

Chr	Position (hg19)	SNP	Major allele	Minor allele	MAF	Coefficient	Standard Error	*P*
2	27847364	rs2384654	T	C	0.4830	-0.0131	0.0024	4.49E-08
2	27592423	rs7602534	T	C	0.3844	0.0261	0.0024	2.09E-27
2	28164553	rs10170872	T	C	0.7839	-0.0152	0.0026	2.89E-09
2	27716494	rs704795	A	G	0.3881	0.0248	0.0024	6.89E-25
2	27878686	rs4666010	T	C	0.7926	-0.0162	0.0026	2.62E-10
2	27832055	rs2384656	A	G	0.7348	0.0335	0.0025	1.17E-42
2	27733452	rs4425043	A	G	0.3851	0.0267	0.0024	1.86E-28
2	27766284	rs4665991	A	G	0.2653	-0.0336	0.0024	7.98E-43
2	28078994	rs7426295	A	G	0.2159	0.0153	0.0025	2.06E-09
2	28151354	rs10185472	A	G	0.7915	-0.0152	0.0026	2.89E-09
2	27815510	rs13022873	A	C	0.7348	0.0336	0.0025	9.32E-43
2	27831607	rs6547735	T	C	0.8120	-0.0171	0.0029	3.24E-09
2	28121190	rs7592580	T	C	0.1736	0.0144	0.0026	3.90E-08
2	27794150	rs4260197	C	G	0.5326	0.0138	0.0024	8.09E-09
2	27827196	rs2141372	T	G	0.2026	0.0200	0.0026	4.63E-15
2	27419282	rs1275501	T	G	0.2864	0.0168	0.0025	1.25E-11
2	27729480	rs2293571	A	G	0.3843	0.0263	0.0024	1.01E-27
2	27807624	rs13026621	A	G	0.4816	-0.0132	0.0024	3.55E-08
2	27626945	rs2911712	A	T	0.6104	-0.0253	0.0024	4.57E-26
2	27730940	rs1260326	T	C	0.4088	-0.0439	0.0024	1.30E-77
2	28205581	rs7577342	T	C	0.7856	-0.0157	0.0025	7.29E-10
2	27786188	rs10208529	A	T	0.7347	0.0336	0.0024	7.92E-43
2	28191629	rs1841068	T	C	0.7863	-0.0145	0.0026	1.58E-08
2	27928797	rs13030973	T	C	0.7885	0.0284	0.0025	2.01E-29
2	27967260	rs13023194	C	G	0.1818	-0.0281	0.0031	1.20E-19
2	27556721	rs3739095	A	G	0.4294	0.0295	0.0024	1.46E-35

Note: In total, 233 SNPs significantly associated with mannose ($P_{mQTL} \leq 5\times10^{-8}$, LD-$r^2$ >0.1) were identified as candidate genetic IVs. The remaining 12 IVs were further examined for potential pleiotropic effects by heterogeneity in dependent instruments (HEIDI) outlier detection. In the end, there are 12 SNPs that meet the requirements of the IVs, including rs2068834, rs10189899, rs4666052, rs2305929, rs7349418, rs1561535, rs11684134, rs13013484, rs11681351, rs6547735, rs2141372, rs13030973, which are

© 2021 Wei Y et al. *JAMA Network Open.*

marked in gray background.

© 2021 Wei Y et al. *JAMA Network Open.*

eTable 4. Association Between SNPs Selected as Genetic IV for Mannose and ARDS Risk in MEARDS GWAS

Chr	Position (hg19)	SNP	Major allele	Minor allele	MAF	*N*	*OR*	Standard Error	*P*
2	27544285	rs1122227	T	C	0.2147	1630	0.7972	0.1078	0.0355
2	27873326	rs8731	G	C	0.2101	1630	1.0640	0.1070	0.5619
2	27504312	rs1992291	G	A	0.2291	1630	0.8154	0.1048	0.0514
2	27825715	rs12467476	C	T	0.2819	1630	1.1150	0.0951	0.2518
2	28418189	rs13000936	A	G	0.3233	1630	1.0260	0.0938	0.7831
2	28225861	rs11692105	T	G	0.2098	1630	0.8616	0.1090	0.1717
2	28199424	rs12465000	G	A	0.2144	1630	1.0120	0.1066	0.9120
2	28344285	rs4401177	A	G	0.1184	1630	1.0790	0.1312	0.5602
2	27853245	rs13002853	G	C	0.2764	1630	1.1250	0.0951	0.2158
2	27839539	rs2068834	C	T	0.2966	1630	1.1000	0.0944	0.3114
2	27688601	rs1647276	T	C	0.3709	1630	0.8070	0.0897	0.0168
2	27508710	rs11686131	T	C	0.2135	1630	0.8091	0.1077	0.0492
2	27579231	rs6760828	C	T	0.3736	1630	0.7967	0.0892	0.0109
2	27743154	rs780092	G	A	0.1512	1630	1.0400	0.1210	0.7439
2	27519153	rs13404327	T	G	0.2672	1630	0.8340	0.1001	0.0698
2	27961344	rs1317580	C	G	0.2748	1630	1.1180	0.0944	0.2370
2	28088580	rs4666028	A	T	0.1709	1630	1.1340	0.1148	0.2739
2	27811722	rs12478841	G	A	0.2825	1630	1.1120	0.0951	0.2647
2	27528692	rs4665963	C	T	0.2678	1630	0.8386	0.1000	0.0784
2	27767107	rs1972669	T	G	0.4972	1630	0.8714	0.0860	0.1093
2	28107692	rs6708889	C	T	0.2064	1630	1.0240	0.1087	0.8264
2	27644464	rs1728922	C	A	0.3715	1630	0.8094	0.0895	0.0181
2	27995931	rs3792252	G	A	0.2488	1630	1.0850	0.1005	0.4154
2	27722416	rs1260320	A	G	0.3699	1630	0.7876	0.0899	0.0079
2	27855397	rs10173720	T	A	0.2107	1630	1.0620	0.1070	0.5746
2	27550967	rs1049817	G	A	0.3736	1630	0.7945	0.0890	0.0098
2	27750546	rs2911711	T	A	0.4822	1630	1.2340	0.0849	0.0134
2	27933642	rs4616435	T	C	0.2347	1630	1.1210	0.1011	0.2583

© 2021 Wei Y et al. *JAMA Network Open.*

Chr	Position (hg19)	SNP	Major allele	Minor allele	MAF	*N*	*OR*	Standard Error	*P*
2	27715207	rs8395	A	T	0.3675	1630	0.8150	0.0898	0.0227
2	28200152	rs2337374	G	A	0.2141	1630	1.0140	0.1066	0.8973
2	28367761	rs6751559	G	T	0.1003	1630	1.2930	0.1361	0.0589
2	28301540	rs937813	C	T	0.1215	1630	1.1540	0.1297	0.2702
2	27801418	rs1919126	C	A	0.4975	1630	0.8754	0.0860	0.1218
2	28281545	rs6547820	G	A	0.2061	1630	1.0260	0.1087	0.8146
2	28523834	rs12617913	C	T	0.3699	1630	0.9063	0.0899	0.2737
2	28232279	rs2839791	A	C	0.2135	1630	1.0190	0.1066	0.8616
2	27839832	rs7571558	G	C	0.2064	1630	1.0790	0.1064	0.4768
2	27789861	rs965813	A	T	0.2043	1630	1.0940	0.1067	0.3999
2	28396483	rs7586645	A	G	0.3196	1630	1.0420	0.0935	0.6585
2	27910706	rs13023094	C	A	0.2298	1630	1.1370	0.1019	0.2071
2	27995781	rs3736594	A	C	0.2546	1630	1.0630	0.0995	0.5375
2	28090059	rs10171517	T	C	0.2080	1630	1.0110	0.1073	0.9153
2	28079344	rs4233719	A	C	0.2150	1630	1.0070	0.1065	0.9457
2	27802805	rs3811644	G	A	0.2043	1630	1.0760	0.1069	0.4959
2	27741237	rs780094	T	C	0.4239	1630	1.2400	0.0861	0.0125
2	27952406	rs12994085	C	T	0.2273	1630	1.1090	0.1025	0.3126
2	27840640	rs4666002	C	G	0.2825	1630	1.1110	0.0951	0.2668
2	27801759	rs1919128	G	A	0.2822	1630	1.1140	0.0951	0.2563
2	27574953	rs4665969	C	T	0.3718	1630	0.7821	0.0894	0.0060
2	27706640	rs2272417	C	T	0.3883	1630	0.7955	0.0888	0.0100
2	27663416	rs1260342	T	G	0.3709	1630	0.8071	0.0895	0.0166
2	27488432	rs6547521	C	G	0.3052	1630	0.8675	0.0949	0.1342
2	27846645	rs1528403	G	C	0.4865	1630	1.1290	0.0861	0.1580
2	27895073	rs2178198	T	C	0.1374	1630	1.1420	0.1235	0.2810
2	28082494	rs4578809	A	G	0.1712	1630	1.1400	0.1147	0.2545
2	27426878	rs1275522	C	T	0.3028	1630	0.8708	0.0948	0.1446
2	27721971	rs813592	C	T	0.3702	1630	0.7910	0.0899	0.0091
2	28256831	rs9309658	A	G	0.2135	1630	1.0190	0.1066	0.8616
2	27637235	rs2010087	T	C	0.3681	1630	0.8144	0.0896	0.0220

© 2021 Wei Y et al. *JAMA Network Open.*

Chr	Position (hg19)	SNP	Major allele	Minor allele	MAF	*N*	*OR*	Standard Error	*P*
2	27613617	rs11891554	A	G	0.0454	1630	1.0270	0.2049	0.8977
2	27424636	rs1395	G	A	0.3172	1630	0.8615	0.0937	0.1115
2	27455726	rs13399758	C	T	0.0629	1630	0.9127	0.1813	0.6144
2	27449293	rs7593448	G	C	0.3043	1630	0.8708	0.0947	0.1440
2	27731212	rs3817588	C	T	0.1748	1630	0.9346	0.1170	0.5633
2	28418031	rs6712705	C	T	0.3252	1630	1.0120	0.0940	0.8949
2	27783801	rs4665382	C	T	0.2822	1630	1.1140	0.0951	0.2563
2	27393030	rs1659689	A	G	0.3064	1630	0.8807	0.0938	0.1758
2	27958855	rs6753736	C	G	0.2279	1630	1.1070	0.1025	0.3221
2	27975394	rs6547796	T	C	0.2503	1630	1.1180	0.0991	0.2591
2	28520083	rs10189899	A	G	0.3613	1630	0.8875	0.0904	0.1866
2	27659491	rs780102	C	T	0.3709	1630	0.8071	0.0895	0.0166
2	28162861	rs4390729	T	C	0.2138	1630	1.0170	0.1066	0.8762
2	27746832	rs8179252	C	A	0.3733	1630	0.7942	0.0898	0.0103
2	27604279	rs4582	G	A	0.3709	1630	0.8053	0.0894	0.0154
2	28162274	rs13416434	T	C	0.2135	1630	1.0190	0.1066	0.8616
2	27742603	rs780093	T	C	0.4236	1630	1.2270	0.0859	0.0172
2	27703495	rs1260345	G	A	0.3926	1630	0.7854	0.0889	0.0066
2	27611469	rs7594812	G	A	0.3712	1630	0.8045	0.0894	0.0149
2	27452784	rs6717980	C	T	0.3037	1630	0.8744	0.0949	0.1571
2	27519254	rs13404446	A	G	0.2672	1630	0.8340	0.1001	0.0698
2	27841305	rs6756238	G	A	0.4969	1630	0.8797	0.0863	0.1372
2	27852637	rs6547738	G	A	0.4896	1630	1.1400	0.0860	0.1285
2	28530720	rs4666052	A	G	0.3623	1630	0.8939	0.0906	0.2157
2	28113911	rs2305929	G	A	0.2015	1630	1.1260	0.1066	0.2673
2	27443196	rs4665958	G	A	0.3037	1630	0.8743	0.0946	0.1559
2	27987046	rs12477908	A	C	0.2552	1630	1.0700	0.0991	0.4932
2	28443050	rs7349418	C	T	0.4745	1630	1.1210	0.0861	0.1843
2	27652153	rs780100	T	G	0.3709	1630	0.8071	0.0895	0.0166
2	28144067	rs1870325	G	A	0.2058	1630	1.0270	0.1087	0.8081
2	27471025	rs1561535	T	C	0.3009	1630	0.8645	0.0952	0.1262

© 2021 Wei Y et al. *JAMA Network Open.*

Chr	Position (hg19)	SNP	Major allele	Minor allele	MAF	*N*	*OR*	Standard Error	*P*
2	28255302	rs937812	C	T	0.2135	1630	1.0190	0.1066	0.8616
2	27851918	rs3749147	A	G	0.2767	1630	1.1150	0.0951	0.2509
2	27932587	rs6727388	G	A	0.2347	1630	1.1210	0.1011	0.2583
2	27801493	rs1919127	C	T	0.2825	1630	1.1120	0.0951	0.2647
2	28213424	rs10175508	C	T	0.2141	1630	1.0140	0.1066	0.8973
2	27748624	rs1260333	A	G	0.4718	1630	1.2240	0.0847	0.0170
2	28018293	rs4666012	G	A	0.1951	1630	1.0550	0.1100	0.6276
2	28258027	rs7593127	G	A	0.2135	1630	1.0190	0.1066	0.8616
2	28517861	rs4666051	A	G	0.3607	1630	0.8916	0.0906	0.2052
2	28140349	rs2337372	A	G	0.1678	1630	1.1540	0.1152	0.2147
2	28219941	rs1458398	A	G	0.2141	1630	1.0140	0.1066	0.8973
2	27968454	rs9967838	A	C	0.2509	1630	1.1410	0.0987	0.1823
2	27818721	rs1919129	G	C	0.2061	1630	1.0860	0.1061	0.4346
2	27536380	rs4665965	C	T	0.2681	1630	0.8251	0.1000	0.0547
2	27693485	rs1647266	C	T	0.3709	1630	0.8070	0.0897	0.0168
2	27450724	rs3769143	A	G	0.3043	1630	0.8708	0.0947	0.1440
2	27648726	rs4665978	C	T	0.4184	1630	0.8159	0.0882	0.0211
2	27432547	rs1275530	A	G	0.3028	1630	0.8708	0.0948	0.1446
2	27755825	rs4665987	A	G	0.2822	1630	1.1430	0.0950	0.1600
2	27441397	rs1275538	A	G	0.3031	1630	0.8697	0.0948	0.1409
2	28094270	rs13016573	G	C	0.1709	1630	1.1340	0.1148	0.2739
2	27430781	rs1275528	A	G	0.3028	1630	0.8708	0.0948	0.1446
2	27812252	rs6760250	A	G	0.2825	1630	1.1120	0.0951	0.2647
2	27567407	rs6743819	T	G	0.3733	1630	0.7921	0.0892	0.0090
2	27791555	rs4665383	G	C	0.2822	1630	1.1140	0.0951	0.2563
2	27600239	rs13472	A	G	0.3663	1630	0.8106	0.0897	0.0192
2	27972833	rs12104449	G	A	0.1279	1630	1.0800	0.1268	0.5462
2	27398196	rs2580754	T	A	0.3083	1630	0.8585	0.0938	0.1040
2	27566520	rs11689803	A	T	0.2436	1630	0.8032	0.1039	0.0349
2	27860258	rs2141371	G	A	0.2721	1630	0.8677	0.0983	0.1486
2	28202936	rs898032	C	G	0.2144	1630	1.0120	0.1066	0.9120

© 2021 Wei Y et al. *JAMA Network Open.*

Chr	Position (hg19)	SNP	Major allele	Minor allele	MAF	*N*	*OR*	Standard Error	*P*
2	28124171	rs10177845	G	A	0.2144	1630	1.0130	0.1065	0.9037
2	27416702	rs1275504	A	T	0.3393	1630	0.8779	0.0919	0.1564
2	28101938	rs6706209	C	T	0.2163	1630	1.0240	0.1054	0.8202
2	27589810	rs2280737	C	T	0.3681	1630	0.8121	0.0895	0.0201
2	27728777	rs2293572	G	C	0.3681	1630	0.8150	0.0898	0.0227
2	27635582	rs1060525	G	A	0.3715	1630	0.8094	0.0895	0.0181
2	28529165	rs10165668	G	A	0.3623	1630	0.8939	0.0906	0.2157
2	28085311	rs7595986	G	A	0.2150	1630	1.0070	0.1065	0.9457
2	27839369	rs4666000	C	T	0.2966	1630	1.1000	0.0944	0.3114
2	28276643	rs1395324	C	G	0.2135	1630	1.0190	0.1066	0.8616
2	27389195	rs1275541	A	G	0.3052	1630	0.8762	0.0942	0.1606
2	27743215	rs814295	G	A	0.1546	1630	1.0170	0.1197	0.8861
2	27830067	rs6719960	G	A	0.2061	1630	1.0860	0.1061	0.4346
2	27848397	rs4665384	G	A	0.4902	1630	1.1400	0.0862	0.1294
2	28085347	rs13021285	C	T	0.1712	1630	1.1400	0.1147	0.2545
2	28019175	rs4666014	A	G	0.2028	1630	1.0300	0.1082	0.7859
2	27778091	rs6706610	G	A	0.4979	1630	0.8735	0.0861	0.1160
2	28168415	rs7577437	A	G	0.2135	1630	1.0190	0.1066	0.8616
2	28087205	rs13004096	T	C	0.1681	1630	1.1520	0.1152	0.2181
2	28080214	rs13025314	A	G	0.1712	1630	1.1400	0.1147	0.2545
2	27685388	rs780110	A	G	0.4175	1630	0.8201	0.0886	0.0252
2	28081630	rs4578808	T	G	0.1712	1630	1.1400	0.1147	0.2545
2	27420690	rs1659685	C	T	0.3028	1630	0.8708	0.0948	0.1446
2	27542041	rs1975384	T	C	0.2135	1630	0.8078	0.1077	0.0475
2	28086765	rs11902158	G	A	0.1712	1630	1.1400	0.1147	0.2545
2	27657167	rs704791	C	T	0.3709	1630	0.8071	0.0895	0.0166
2	28271435	rs13402420	T	G	0.2135	1630	1.0190	0.1066	0.8616
2	27515105	rs4665962	G	A	0.2135	1630	0.8091	0.1077	0.0492
2	27847606	rs6749426	C	T	0.4902	1630	1.1400	0.0862	0.1294
2	27981655	rs12471347	C	G	0.2417	1630	1.1020	0.1012	0.3384
2	28199736	rs12465103	G	C	0.2141	1630	1.0140	0.1066	0.8973

© 2021 Wei Y et al. *JAMA Network Open.*

Chr	Position (hg19)	SNP	Major allele	Minor allele	MAF	*N*	*OR*	Standard Error	*P*
2	27677691	rs780104	A	G	0.3702	1630	0.8085	0.0896	0.0177
2	27830990	rs6547734	G	T	0.4975	1630	0.8754	0.0860	0.1218
2	27718474	rs780090	T	C	0.0844	1630	1.4760	0.1457	0.0076
2	28209283	rs10209126	C	T	0.2141	1630	1.0140	0.1066	0.8973
2	28023120	rs11127125	C	T	0.2028	1630	1.0080	0.1085	0.9410
2	28098724	rs13411592	G	A	0.2150	1630	1.0070	0.1065	0.9457
2	28118662	rs7607546	G	A	0.1712	1630	1.1280	0.1145	0.2936
2	27934731	rs6727215	A	G	0.2347	1630	1.1210	0.1011	0.2583
2	28090820	rs898034	T	C	0.2080	1630	1.0110	0.1073	0.9153
2	27980944	rs10209020	C	T	0.2497	1630	1.1020	0.0992	0.3294
2	27774702	rs2384628	G	T	0.4905	1630	1.1670	0.0861	0.0724
2	27684734	rs780107	G	A	0.3709	1630	0.8070	0.0897	0.0168
2	27558252	rs11684134	G	A	0.4153	1630	0.8430	0.0860	0.0470
2	27568565	rs10205219	C	T	0.3736	1630	0.7913	0.0892	0.0087
2	28020966	rs3935148	A	T	0.1939	1630	1.0330	0.1108	0.7686
2	27796526	rs10165098	C	T	0.4988	1630	0.8688	0.0862	0.1027
2	28136351	rs1458396	A	G	0.2144	1630	1.0130	0.1065	0.9066
2	27460968	rs1141313	G	A	0.3037	1630	0.8744	0.0949	0.1571
2	27730817	rs8179219	A	G	0.0472	1630	0.9546	0.2046	0.8202
2	27712408	rs7583698	T	C	0.0485	1630	1.0200	0.2000	0.9192
2	27613031	rs12476704	A	C	0.3669	1630	0.8095	0.0896	0.0183
2	27951658	rs867282	T	C	0.2202	1630	1.1070	0.1046	0.3307
2	27715416	rs2303369	T	C	0.3669	1630	0.8186	0.0897	0.0256
2	28189280	rs1902966	G	A	0.2138	1630	1.0150	0.1066	0.8868
2	27892023	rs2272406	T	A	0.2294	1630	1.1410	0.1019	0.1957
2	28155195	rs6547815	T	A	0.2058	1630	1.0270	0.1087	0.8081
2	27698343	rs780117	G	C	0.3702	1630	0.8085	0.0896	0.0177
2	27988821	rs13013484	G	A	0.2663	1630	1.0780	0.0978	0.4442
2	27772879	rs6749052	A	T	0.4979	1630	0.8735	0.0861	0.1160
2	28268467	rs6738887	C	T	0.2135	1630	1.0190	0.1066	0.8616
2	27631191	rs7563162	T	C	0.3718	1630	0.8088	0.0895	0.0177

© 2021 Wei Y et al. *JAMA Network Open.*

Chr	Position (hg19)	SNP	Major allele	Minor allele	MAF	*N*	*OR*	Standard Error	*P*
2	27681598	rs780106	C	A	0.3709	1630	0.8070	0.0897	0.0168
2	27646770	rs6547626	T	C	0.3715	1630	0.8094	0.0895	0.0181
2	28394293	rs17759746	C	T	0.0997	1630	1.2800	0.1364	0.0699
2	28235184	rs7602328	G	A	0.2135	1630	1.0190	0.1066	0.8616
2	27663215	rs1260341	A	T	0.3709	1630	0.8071	0.0895	0.0166
2	27441302	rs1275537	C	G	0.3031	1630	0.8697	0.0948	0.1409
2	27743423	rs11681351	A	G	0.3733	1630	0.7942	0.0898	0.0103
2	28261163	rs6731650	C	A	0.2135	1630	1.0190	0.1066	0.8616
2	27608115	rs1647284	T	C	0.3712	1630	0.8045	0.0894	0.0149
2	27808154	rs6734059	C	T	0.2052	1630	1.0850	0.1062	0.4442
2	27595756	rs1528533	C	G	0.3709	1630	0.8011	0.0895	0.0132
2	28143017	rs10173848	G	T	0.2003	1630	1.0020	0.1099	0.9823
2	28003174	rs13030345	T	G	0.1985	1630	1.1030	0.1062	0.3556
2	28029649	rs4665392	G	A	0.2040	1630	1.0060	0.1084	0.9533
2	27640325	rs4665976	G	A	0.3715	1630	0.8094	0.0895	0.0181
2	27844601	rs1881396	G	T	0.2046	1630	1.0640	0.1069	0.5632
2	27725761	rs715326	T	C	0.3666	1630	0.8041	0.0899	0.0153
2	27667297	rs4803	G	A	0.3709	1630	0.8070	0.0897	0.0168
2	27462405	rs2014252	A	T	0.3040	1630	0.8739	0.0948	0.1552
2	28079775	rs4233720	G	A	0.1712	1630	1.1400	0.1147	0.2545
2	27851120	rs12615690	C	T	0.4902	1630	1.1400	0.0862	0.1294
2	27642734	rs11675428	C	A	0.1433	1630	0.8326	0.1283	0.1534
2	27584666	rs7586601	G	A	0.4144	1630	0.8177	0.0876	0.0216
2	27796927	rs6704596	G	A	0.2037	1630	1.0800	0.1072	0.4725
2	27847364	rs2384654	C	T	0.4957	1630	0.8836	0.0862	0.1511
2	27592423	rs7602534	T	C	0.3672	1630	0.8082	0.0898	0.0177
2	28164553	rs10170872	C	T	0.2135	1630	1.0190	0.1066	0.8616
2	27716494	rs704795	A	G	0.3712	1630	0.8095	0.0897	0.0184
2	27878686	rs4666010	C	T	0.2098	1630	1.0570	0.1073	0.6033
2	27832055	rs2384656	G	A	0.2822	1630	1.1130	0.0951	0.2601
2	27733452	rs4425043	A	G	0.3675	1630	0.8118	0.0899	0.0205

© 2021 Wei Y et al. *JAMA Network Open.*

Chr	Position (hg19)	SNP	Major allele	Minor allele	MAF	*N*	*OR*	Standard Error	*P*
2	27766284	rs4665991	A	G	0.2804	1630	1.1290	0.0954	0.2049
2	28078994	rs7426295	A	G	0.2150	1630	1.0070	0.1065	0.9457
2	28151354	rs10185472	G	A	0.2058	1630	1.0270	0.1087	0.8081
2	27815510	rs13022873	C	A	0.2825	1630	1.1100	0.0950	0.2715
2	27831607	rs6547735	C	T	0.2061	1630	1.0860	0.1061	0.4346
2	28121190	rs7592580	T	C	0.1681	1630	1.1520	0.1152	0.2181
2	27794150	rs4260197	G	C	0.4868	1630	1.1550	0.0862	0.0947
2	27827196	rs2141372	T	G	0.2061	1630	1.0860	0.1061	0.4346
2	27419282	rs1275501	T	G	0.3028	1630	0.8708	0.0948	0.1446
2	27729480	rs2293571	A	G	0.3681	1630	0.8150	0.0898	0.0227
2	27807624	rs13026621	G	A	0.4979	1630	0.8735	0.0861	0.1160
2	27626945	rs2911712	T	A	0.3718	1630	0.8129	0.0893	0.0203
2	27730940	rs1260326	T	C	0.4310	1630	1.2070	0.0861	0.0290
2	28205581	rs7577342	C	T	0.2150	1630	1.0080	0.1067	0.9422
2	27786188	rs10208529	T	A	0.2825	1630	1.1120	0.0951	0.2647
2	28191629	rs1841068	C	T	0.2132	1630	1.0020	0.1072	0.9862
2	27928797	rs13030973	C	T	0.2347	1630	1.1210	0.1011	0.2583
2	27967260	rs13023194	C	G	0.1933	1630	1.0940	0.1069	0.4025
2	27556721	rs3739095	A	G	0.4120	1630	0.8015	0.0878	0.0117

Note: The remaining 12 IVs were further examined for potential pleiotropic effects by heterogeneity in dependent instruments (HEIDI) outlier detection. In the end, there are 12 SNPs that meet the requirements of the IVs, including rs2068834, rs10189899, rs4666052, rs2305929, rs7349418, rs1561535, rs11684134, rs13013484, rs11681351, rs6547735, rs2141372, rs13030973, which are marked in gray gackground.

© 2021 Wei Y et al. *JAMA Network Open.*

eTable 5. Pearson's Correlation Coefficient *r* Between Pairs of Metabolites (X-11440 and X-12850) Measured in Metabolome-wide Association Study

Metabolite	Pathway	Super pathway	X-11440	X-12850
4-androsten-3beta,17beta-diol disulfate 1*	Sterol, Steroid	Lipid	0.77	0.26
4-androsten-3beta,17beta-diol disulfate 2*	Sterol, Steroid	Lipid	0.76	0.19
dehydroisoandrosterone sulfate (DHEA-S)	Sterol, Steroid	Lipid	0.66	0.11
5alpha-androstan-3beta,17beta-diol disulfate	Sterol, Steroid	Lipid	0.61	0.22
androsterone sulfate	Sterol, Steroid	Lipid	0.47	0.08
epiandrosterone sulfate	Sterol, Steroid	Lipid	0.46	0.06
5alpha-pregnan-3beta,20alpha-diol disulfate	Sterol, Steroid	Lipid	0.35	0.08
3-(4-hydroxyphenyl)lactate	Phenylalanine & tyrosine metabolism	Amino acid	0.23	0.13
alpha-hydroxyisovalerate	Valine, leucine and isoleucine metabolism	Amino acid	0.23	0.20
urate	Purine metabolism, urate metabolism	Nucleotide	0.21	0.07
X-12244--N-acetylcarnosine	Dipeptide	Peptide	0.20	0.03
3-methyl-2-oxovalerate	Valine, leucine and isoleucine metabolism	Amino acid	0.20	0.13
4-methyl-2-oxopentanoate	Valine, leucine and isoleucine metabolism	Amino acid	0.18	0.08
1,5-anhydroglucitol (1,5-AG)	Glycolysis, gluconeogenesis, pyruvate metabolism	Carbohydrate	0.14	0.06
creatinine	Creatine metabolism	Amino acid	0.14	0.07
cortisone	Sterol, Steroid	Lipid	0.14	0.10
leucine	Valine, leucine and isoleucine metabolism	Amino acid	0.13	0.07
isoleucine	Valine, leucine and isoleucine metabolism	Amino acid	0.12	0.11
3-methyl-2-oxobutyrate	Valine, leucine and isoleucine metabolism	Amino acid	0.12	0.05
cortisol	Sterol, Steroid	Lipid	0.12	0.13
lactate	Glycolysis, gluconeogenesis, pyruvate metabolism	Carbohydrate	0.12	0.08
phenyllactate (PLA)	Phenylalanine & tyrosine metabolism	Amino acid	0.11	0.16
2-hydroxybutyrate (AHB)	Cysteine, methionine, SAM, taurine metabolism	Amino acid	0.11	0.02

© 2021 Wei Y et al. *JAMA Network Open.*

10-undecenoate (11:1n1)	Medium chain fatty acid	Lipid	0.11	0.00
aspartate	Alanine and aspartate metabolism	Amino acid	0.10	0.11
proline	Urea cycle; arginine-, proline-, metabolism	Amino acid	0.10	0.11
2-aminobutyrate	Butanoate metabolism	Amino acid	0.10	0.00
carbamazepine*	Drug	Xenobiotics	0.10	0.12
bilirubin (E,E)*	Hemoglobin and porphyrin metabolism	Cofactors and vitamins	0.10	0.04
biliverdin	Hemoglobin and porphyrin metabolism	Cofactors and vitamins	0.09	0.04
metoprolol acid metabolite*	Drug	Xenobiotics	0.09	0.03
gamma-glutamylglutamate	gamma-glutamyl	Peptide	0.09	0.21
X-12510--2-aminooctanoic acid	Amino fatty acid	Amino acid	0.09	0.10
1-arachidonoylglycerophosphoinositol*	Lysolipid	Lipid	0.09	0.13
hexadecanedioate	Fatty acid, dicarboxylate	Lipid	0.09	0.16
valine	Valine, leucine and isoleucine metabolism	Amino acid	0.09	0.03
bilirubin (E,Z or Z,E)*	Hemoglobin and porphyrin metabolism	Cofactors and vitamins	0.08	0.06
pipecolate	Lysine metabolism	Amino acid	0.08	0.11
oleoylcarnitine	Carnitine metabolism	Lipid	0.08	0.06
adrenate (22:4n6)	Long chain fatty acid	Lipid	0.08	0.07
taurochenodeoxycholate	Bile acid metabolism	Lipid	0.08	0.33
pyroglutamine*	Glutamate metabolism	Amino acid	0.08	0.09
gamma-glutamylvaline	gamma-glutamyl	Peptide	0.08	0.03
beta-hydroxyisovalerate	Valine, leucine and isoleucine metabolism	Amino acid	0.08	0.04
piperine	Food component, Plant	Xenobiotics	0.08	0.05
X-12441--12-hydroxyeicosatetraenoate (12-HETE)	Eicosanoid	Lipid	0.08	0.05
gamma-glutamylleucine	gamma-glutamyl	Peptide	0.08	0.04
X-12990--docosapentaenoic acid (n6-DPA)	Essential fatty acid	Lipid	0.08	0.05
dihomo-linoleate (20:2n6)	Long chain fatty acid	Lipid	0.08	0.04
threonine	Glycine, serine and threonine metabolism	Amino acid	0.08	0.09
indolelactate	Tryptophan metabolism	Amino acid	0.08	0.08
2-tetradecenoyl carnitine	Carnitine metabolism	Lipid	0.07	0.07
palmitoylcarnitine	Carnitine metabolism	Lipid	0.07	0.05
2-methylbutyroylcarnitine	Valine, leucine and isoleucine metabolism	Amino acid	0.07	0.07

© 2021 Wei Y et al. *JAMA Network Open.*

malate	Krebs cycle	Energy	0.07	0.12
gamma-glutamylmethionine*	gamma-glutamyl	Peptide	0.07	0.03
1,3,7-trimethylurate	Xanthine metabolism	Xenobiotics	0.07	0.10
palmitate (16:0)	Long chain fatty acid	Lipid	0.07	0.03
3-hydroxybutyrate (BHBA)	Ketone bodies	Lipid	0.07	0.07
methionine	Cysteine, methionine, SAM, taurine metabolism	Amino acid	0.07	0.09
phenylalanine	Phenylalanine & tyrosine metabolism	Amino acid	0.07	0.08
gamma-glutamylisoleucine*	gamma-glutamyl	Peptide	0.07	0.09
tetradecanedioate	Fatty acid, dicarboxylate	Lipid	0.07	0.12
isovalerylcarnitine	Valine, leucine and isoleucine metabolism	Amino acid	0.07	0.02
X-12100--hydroxytryptophan*	Tryptophan metabolism	Amino acid	0.07	0.05
4-vinylphenol sulfate	Benzoate metabolism	Xenobiotics	0.07	0.09
tryptophan betaine	Tryptophan metabolism	Amino acid	0.06	0.06
aspartylphenylalanine	Dipeptide	Peptide	0.06	0.13
taurolithocholate 3-sulfate	Bile acid metabolism	Lipid	0.06	0.35
isovalerate	Fatty acid metabolism	Lipid	0.06	0.03
N2,N2-dimethylguanosine	Purine metabolism, guanine containing	Nucleotide	0.06	0.09
tryptophan	Tryptophan metabolism	Amino acid	0.06	0.04
1-palmitoleoylglycerophosphocholine*	Lysolipid	Lipid	0.06	0.08
gamma-tocopherol	Tocopherol metabolism	Cofactors and vitamins	0.06	0.00
gamma-glutamylthreonine*	gamma-glutamyl	Peptide	0.06	0.06
laurylcarnitine	Carnitine metabolism	Lipid	0.06	0.08
2-linoleoylglycerophosphocholine*	Lysolipid	Lipid	0.06	0.08
octadecanedioate	Fatty acid, dicarboxylate	Lipid	0.06	0.12
glycocholate	Bile acid metabolism	Lipid	0.06	0.39
5-oxoproline	Glutathione metabolism	Amino acid	0.06	0.04
hyodeoxycholate	Bile acid metabolism	Lipid	0.06	0.05
glycoursodeoxycholate	Bile acid metabolism	Lipid	0.06	0.32
mannose	Fructose, mannose, galactose, starch, and sucrose metabolism	Carbohydrate	0.06	0.00
X-11593--O-methylascorbate*	Ascorbate and aldarate metabolism	Cofactors and vitamins	0.06	0.03
acetylcarnitine	Carnitine metabolism	Lipid	0.06	0.04

© 2021 Wei Y et al. *JAMA Network Open.*

gamma-glutamylphenylalanine	gamma-glutamyl	Peptide	0.06	0.08
glutaroyl carnitine	Lysine metabolism	Amino acid	0.06	0.02
glutamate	Glutamate metabolism	Amino acid	0.05	0.03
arachidonate (20:4n6)	Long chain fatty acid	Lipid	0.05	0.00
1-arachidonoylglycerophosphoethanolamine*	Lysolipid	Lipid	0.05	0.11
2-palmitoylglycerophosphocholine*	Lysolipid	Lipid	0.05	0.05
glycylvaline	Dipeptide	Peptide	0.05	0.07
N-acetylthreonine	Glycine, serine and threonine metabolism	Amino acid	0.05	0.06
oleate (18:1n9)	Long chain fatty acid	Lipid	0.05	0.02
N-acetylalanine	Alanine and aspartate metabolism	Amino acid	0.05	0.05
N-(2-furoyl)glycine	Food component, Plant	Xenobiotics	0.05	-0.02
carnitine	Carnitine metabolism	Lipid	0.04	-0.01
1-linoleoylglycerophosphoethanolamine*	Lysolipid	Lipid	0.04	0.14
dihomo-linolenate (20:3n3 or n6)	Essential fatty acid	Lipid	0.04	0.00
2-hydroxypalmitate	Fatty acid, monohydroxy	Lipid	0.04	0.07
cotinine	Tobacco metabolite	Xenobiotics	0.04	0.09
decanoylcarnitine	Carnitine metabolism	Lipid	0.04	0.01
stearate (18:0)	Long chain fatty acid	Lipid	0.04	0.02
7-methylguanine	Purine metabolism, guanine containing	Nucleotide	0.04	0.03
1-linoleoylglycerophosphocholine	Lysolipid	Lipid	0.04	0.07
phenylalanylphenylalanine	Dipeptide	Peptide	0.04	0.04
glycochenodeoxycholate	Bile acid metabolism	Lipid	0.04	0.39
1-palmitoylglycerophosphoinositol*	Lysolipid	Lipid	0.04	0.12
1-arachidonoylglycerophosphocholine*	Lysolipid	Lipid	0.04	0.02
histidine	Histidine metabolism	Amino acid	0.04	0.02
eicosenoate (20:1n9 or 11)	Long chain fatty acid	Lipid	0.04	0.02
serotonin (5HT)	Tryptophan metabolism	Amino acid	0.04	0.04
pseudouridine	Pyrimidine metabolism, uracil containing	Nucleotide	0.04	0.05
2-oleoylglycerophosphocholine*	Lysolipid	Lipid	0.04	0.08
hexanoylcarnitine	Carnitine metabolism	Lipid	0.04	0.02
1-oleoylglycerophosphoethanolamine	Lysolipid	Lipid	0.04	0.19
lathosterol	Sterol, Steroid	Lipid	0.03	-0.02

© 2021 Wei Y et al. *JAMA Network Open.*

alpha-ketoglutarate	Krebs cycle	Energy	0.03	0.06
cyclo(leu-pro)	Dipeptide	Peptide	0.03	0.12
1-oleoylglycerol (1-monoolein)	Monoacylglycerol	Lipid	0.03	0.01
hydroxyisovaleroyl carnitine	Valine, leucine and isoleucine metabolism	Amino acid	0.03	0.02
guanosine	Purine metabolism, guanine containing	Nucleotide	0.03	-0.05
thymol sulfate	Food component, Plant	Xenobiotics	0.03	0.03
2-hydroxyglutarate	Fatty acid, dicarboxylate	Lipid	0.03	0.09
xanthine	Purine metabolism, (hypo)xanthine, inosine containing	Nucleotide	0.03	-0.01
theophylline	Xanthine metabolism	Xenobiotics	0.03	-0.01
1-eicosatrienoylglycerophosphocholine*	Lysolipid	Lipid	0.03	0.04
linoleate (18:2n6)	Essential fatty acid	Lipid	0.03	0.02
butyrylcarnitine	Fatty acid metabolism (also BCAA metabolism)	Lipid	0.03	0.04
taurocholate	Bile acid metabolism	Lipid	0.03	0.26
hypoxanthine	Purine metabolism, (hypo)xanthine, inosine containing	Nucleotide	0.03	0.03
1-oleoylglycerophosphocholine	Lysolipid	Lipid	0.03	0.06
stearoylcarnitine	Carnitine metabolism	Lipid	0.03	0.01
atenolol	Drug	Xenobiotics	0.03	0.02
1-palmitoylglycerophosphocholine	Lysolipid	Lipid	0.03	0.02
choline	Glycerolipid metabolism	Lipid	0.03	0.07
palmitoleate (16:1n7)	Long chain fatty acid	Lipid	0.02	0.02
glucose	Glycolysis, gluconeogenesis, pyruvate metabolism	Carbohydrate	0.02	-0.01
theobromine	Xanthine metabolism	Xenobiotics	0.02	0.00
cis-4-decenoyl carnitine	Carnitine metabolism	Lipid	0.02	0.02
cholate	Bile acid metabolism	Lipid	0.02	0.08
1-palmitoylplasmenylethanolamine*	Lysolipid	Lipid	0.02	0.01
HWESASXX*	Polypeptide	Peptide	0.02	0.01
cysteine-glutathione disulfide	Glutathione metabolism	Amino acid	0.02	-0.05
asparagine	Alanine and aspartate metabolism	Amino acid	0.02	0.04
1-eicosadienoylglycerophosphocholine*	Lysolipid	Lipid	0.02	0.05
pyruvate	Glycolysis, gluconeogenesis, pyruvate metabolism	Carbohydrate	0.02	0.00
ornithine	Urea cycle; arginine-, proline-, metabolism	Amino acid	0.02	0.03

© 2021 Wei Y et al. *JAMA Network Open.*

3-methylhistidine	Histidine metabolism	Amino acid	0.02	-0.01
7-alpha-hydroxy-3-oxo-4-cholestenoate (7-Hoca)	Sterol, Steroid	Lipid	0.02	0.08
X-12442--5,8-tetradecadienoate	Long chain fatty acid	Lipid	0.02	0.02
betaine	Glycine, serine and threonine metabolism	Amino acid	0.02	0.04
N1-methyladenosine	Purine metabolism, adenine containing	Nucleotide	0.02	0.05
trans-4-hydroxyproline	Urea cycle; arginine-, proline-, metabolism	Amino acid	0.02	0.06
1-linoleoylglycerol (1-monolinolein)	Monoacylglycerol	Lipid	0.02	0.08
bradykinin, des-arg(9)	Polypeptide	Peptide	0.02	-0.02
octanoylcarnitine	Carnitine metabolism	Lipid	0.02	-0.01
10-nonadecenoate (19:1n9)	Long chain fatty acid	Lipid	0.02	0.00
heme*	Hemoglobin and porphyrin metabolism	Cofactors and vitamins	0.02	0.03
gamma-glutamyltyrosine	gamma-glutamyl	Peptide	0.02	0.05
glycerophosphorylcholine (GPC)	Glycerolipid metabolism	Lipid	0.01	-0.03
ADSGEGDFXAEGGGVR*	Fibrinogen cleavage peptide	Peptide	0.01	0.00
caffeine	Xanthine metabolism	Xenobiotics	0.01	0.03
1-stearoylglycerophosphocholine	Lysolipid	Lipid	0.01	0.00
allantoin	Purine metabolism, urate metabolism	Nucleotide	0.01	0.07
leucylleucine	Dipeptide	Peptide	0.01	0.01
1-myristoylglycerophosphocholine	Lysolipid	Lipid	0.01	0.01
X-14450--phenylalanylleucine	Dipeptide	Peptide	0.01	0.11
bilirubin (Z,Z)	Hemoglobin and porphyrin metabolism	Cofactors and vitamins	0.01	0.00
1-stearoylglycerophosphoinositol	Lysolipid	Lipid	0.01	0.04
glycodeoxycholate	Bile acid metabolism	Lipid	0.01	0.20
alanine	Alanine and aspartate metabolism	Amino acid	0.01	-0.03
X-14205--alpha-glutamyltyrosine	Dipeptide	Peptide	0.01	0.07
erythrose	Fructose, mannose, galactose, starch, and sucrose metabolism	Carbohydrate	0.01	0.03
erythritol	Sugar, sugar substitute, starch	Xenobiotics	0.01	0.06
tyrosine	Phenylalanine & tyrosine metabolism	Amino acid	0.01	0.04
X-04499--3,4-dihydroxybutyrate	Butanoate metabolism	Amino acid	0.01	0.10
linolenate [alpha or gamma; (18:3n3 or 6)]	Essential fatty acid	Lipid	0.01	0.01
7-methylxanthine	Xanthine metabolism	Xenobiotics	0.00	-0.03

© 2021 Wei Y et al. *JAMA Network Open.*

pantothenate	Pantothenate and CoA metabolism	Cofactors and vitamins	0.00	-0.04
indoleacetate	Tryptophan metabolism	Amino acid	0.00	0.00
DSGEGDFXAEGGGVR*	Fibrinogen cleavage peptide	Peptide	0.00	0.00
3-methoxytyrosine	Phenylalanine & tyrosine metabolism	Amino acid	0.00	0.02
dimethylarginine (SDMA + ADMA)	Urea cycle; arginine-, proline-, metabolism	Amino acid	0.00	0.02
2-stearoylglycerophosphocholine*	Lysolipid	Lipid	0.00	0.01
2-hydroxystearate	Fatty acid, monohydroxy	Lipid	0.00	0.03
pyroglutamylglycine	Dipeptide	Peptide	0.00	0.07
lysine	Lysine metabolism	Amino acid	0.00	-0.03
paraxanthine	Xanthine metabolism	Xenobiotics	0.00	-0.02
X-13431--nonanoylcarnitine*	Carnitine metabolism	Lipid	0.00	0.02
5-dodecenoate (12:1n7)	Medium chain fatty acid	Lipid	0.00	0.05
cysteine	Cysteine, methionine, SAM, taurine metabolism	Amino acid	0.00	0.01
n-Butyl Oleate	Fatty acid, ester	Lipid	0.00	0.01
uridine	Pyrimidine metabolism, uracil containing	Nucleotide	0.00	-0.15
15-methylpalmitate (isobar with 2-methylpalmitate)	Fatty acid, branched	Lipid	0.00	-0.02
pro-hydroxy-pro	Dipeptide	Peptide	-0.01	0.01
ursodeoxycholate	Bile acid metabolism	Lipid	-0.01	0.07
10-heptadecenoate (17:1n7)	Long chain fatty acid	Lipid	-0.01	-0.02
myristate (14:0)	Long chain fatty acid	Lipid	-0.01	0.00
indolepropionate	Tryptophan metabolism	Amino acid	-0.01	-0.01
glycerol 2-phosphate	Chemical	Xenobiotics	-0.01	0.00
inosine	Purine metabolism, (hypo)xanthine, inosine containing	Nucleotide	-0.01	-0.04
erythronate*	Aminosugars metabolism	Carbohydrate	-0.01	0.02
kynurenine	Tryptophan metabolism	Amino acid	-0.01	0.03
N-acetylornithine	Urea cycle; arginine-, proline-, metabolism	Amino acid	-0.01	0.06
X-11786--methylcysteine	Cysteine, methionine, SAM, taurine metabolism	Amino acid	-0.02	-0.05
4-hydroxyhippurate	Benzoate metabolism	Xenobiotics	-0.02	0.06
taurodeoxycholate	Bile acid metabolism	Lipid	-0.02	0.09
margarate (17:0)	Long chain fatty acid	Lipid	-0.02	-0.04
arginine	Urea cycle; arginine-, proline-, metabolism	Amino acid	-0.02	-0.01

© 2021 Wei Y et al. *JAMA Network Open.*

valerate	Short chain fatty acid	Lipid	-0.02	0.01
C-glycosyltryptophan*	Tryptophan metabolism	Amino acid	-0.02	0.01
succinylcarnitine	Krebs cycle	Energy	-0.02	0.01
scyllo-inositol	Inositol metabolism	Lipid	-0.02	0.05
myo-inositol	Inositol metabolism	Lipid	-0.02	0.02
1-palmitoylglycerol (1-monopalmitin)	Monoacylglycerol	Lipid	-0.02	-0.01
nonadecanoate (19:0)	Long chain fatty acid	Lipid	-0.02	0.00
glycerol	Glycerolipid metabolism	Lipid	-0.02	0.04
glycerol 3-phosphate (G3P)	Glycerolipid metabolism	Lipid	-0.02	-0.03
ADpSGEGDFXAEGGGVR*	Fibrinogen cleavage peptide	Peptide	-0.02	0.00
4-acetamidobutanoate	Guanidino and acetamido metabolism	Amino acid	-0.02	0.05
arabinose	Nucleotide sugars, pentose metabolism	Carbohydrate	-0.02	0.06
docosapentaenoate (n3 DPA; 22:5n3)	Essential fatty acid	Lipid	-0.03	-0.06
3-indoxyl sulfate	Tryptophan metabolism	Amino acid	-0.03	-0.05
X-13183--stearamide	Fatty acid amide	Lipid	-0.03	-0.02
propionylcarnitine	Fatty acid metabolism (also BCAA metabolism)	Lipid	-0.03	-0.03
dodecanedioate	Fatty acid, dicarboxylate	Lipid	-0.03	0.06
1,7-dimethylurate	Xanthine metabolism	Xenobiotics	-0.03	-0.03
ibuprofen	Drug	Xenobiotics	-0.03	0.00
serine	Glycine, serine and threonine metabolism	Amino acid	-0.03	-0.02
myristoleate (14:1n5)	Long chain fatty acid	Lipid	-0.03	0.00
chiro-inositol	Inositol metabolism	Lipid	-0.03	0.03
creatine	Creatine metabolism	Amino acid	-0.04	-0.07
gamma-glutamylglutamine	gamma-glutamyl	Peptide	-0.04	-0.04
1-stearoylglycerol (1-monostearin)	Monoacylglycerol	Lipid	-0.04	-0.02
fructose	Fructose, mannose, galactose, starch, and sucrose metabolism	Carbohydrate	-0.04	0.00
glutamine	Glutamate metabolism	Amino acid	-0.04	-0.03
X-14304--leucylalanine	Dipeptide	Peptide	-0.04	-0.02
phenol sulfate	Phenylalanine & tyrosine metabolism	Amino acid	-0.04	0.00
homocitrulline	Urea cycle; arginine-, proline-, metabolism	Amino acid	-0.04	0.06
pentadecanoate (15:0)	Long chain fatty acid	Lipid	-0.04	-0.06
3-methylxanthine	Xanthine metabolism	Xenobiotics	-0.04	-0.02
stachydrine	Food component, Plant	Xenobiotics	-0.04	0.02

© 2021 Wei Y et al. *JAMA Network Open.*

caproate (6:0)	Medium chain fatty acid	Lipid	-0.04	0.00
1-methylxanthine	Xanthine metabolism	Xenobiotics	-0.04	-0.02
mannitol	Fructose, mannose, galactose, starch, and sucrose metabolism	Carbohydrate	-0.04	0.04
saccharin	Food component, Plant	Xenobiotics	-0.04	0.05
1-stearoylglycerophosphoethanolamine	Lysolipid	Lipid	-0.04	0.02
undecanoate (11:0)	Medium chain fatty acid	Lipid	-0.05	-0.03
X-14977--vanillin	Food component/Plant	Xenobiotics	-0.05	0.04
1-heptadecanoylglycerophosphocholine	Lysolipid	Lipid	-0.05	-0.05
1,6-anhydroglucose	Glycolysis, gluconeogenesis, pyruvate metabolism	Carbohydrate	-0.05	0.08
X-12095--N1-methyl-3-pyridone-4-carboxamide	NAD metabolism	Nucleotide	-0.05	-0.08
2-hydroxyisobutyrate	Valine, leucine and isoleucine metabolism	Amino acid	-0.05	-0.04
deoxycholate	Bile acid metabolism	Lipid	-0.05	-0.03
alpha-tocopherol	Tocopherol metabolism	Cofactors and vitamins	-0.05	-0.05
levulinate (4-oxovalerate)	Valine, leucine and isoleucine metabolism	Amino acid	-0.05	-0.01
threonate	Ascorbate and aldarate metabolism	Cofactors and vitamins	-0.06	-0.06
N-acetylglycine	Glycine, serine and threonine metabolism	Amino acid	-0.06	-0.02
1-palmitoylglycerophosphoethanolamine	Lysolipid	Lipid	-0.06	0.05
glycerate	Glycolysis, gluconeogenesis, pyruvate metabolism	Carbohydrate	-0.06	-0.04
4-ethylphenylsulfate	Benzoate metabolism	Xenobiotics	-0.06	0.00
X-14189--leucylalanine	Dipeptide	Peptide	-0.06	-0.04
heptanoate (7:0)	Medium chain fatty acid	Lipid	-0.06	-0.03
1-methylurate	Xanthine metabolism	Xenobiotics	-0.06	-0.01
citrate	Krebs cycle	Energy	-0.07	-0.02
caprate (10:0)	Medium chain fatty acid	Lipid	-0.07	0.03
caprylate (8:0)	Medium chain fatty acid	Lipid	-0.07	0.02
3-(3-hydroxyphenyl)propionate	Phenylalanine & tyrosine metabolism	Amino acid	-0.07	0.00
salicylate	Drug	Xenobiotics	-0.07	-0.01
cholesterol	Sterol, Steroid	Lipid	-0.07	-0.08
pyridoxate	Vitamin B6 metabolism	Cofactors and vitamins	-0.07	-0.07

© 2021 Wei Y et al. *JAMA Network Open.*

benzoate	Benzoate metabolism	Xenobiotics	-0.07	-0.01
ergothioneine	Food component, Plant	Xenobiotics	-0.07	-0.07
3-dehydrocarnitine*	Carnitine metabolism	Lipid	-0.07	0.02
2-hydroxyhippurate (salicylurate)	Benzoate metabolism	Xenobiotics	-0.07	0.02
estrone 3-sulfate	Sterol, Steroid	Lipid	-0.08	-0.06
laurate (12:0)	Medium chain fatty acid	Lipid	-0.08	-0.01
X-14208--phenylalanylserine	Dipeptide	Peptide	-0.08	0.07
acetylphosphate	Oxidative phosphorylation	Energy	-0.08	-0.06
palmitoyl sphingomyelin	Sphingolipid	Lipid	-0.08	-0.06
pelargonate (9:0)	Medium chain fatty acid	Lipid	-0.09	-0.06
docosahexaenoate (DHA; 22:6n3)	Essential fatty acid	Lipid	-0.10	-0.07
stearidonate (18:4n3)	Long chain fatty acid	Lipid	-0.10	-0.05
1-docosahexaenoylglycerophosphocholine*	Lysolipid	Lipid	-0.10	-0.10
salicyluric glucuronide*	Drug	Xenobiotics	-0.10	-0.05
urea	Urea cycle; arginine-, proline-, metabolism	Amino acid	-0.11	-0.06
phosphate	Oxidative phosphorylation	Energy	-0.11	-0.05
3-phenylpropionate (hydrocinnamate)	Phenylalanine & tyrosine metabolism	Amino acid	-0.11	-0.05
quinate	Food component, Plant	Xenobiotics	-0.11	-0.04
catechol sulfate	Benzoate metabolism	Xenobiotics	-0.12	-0.01
citrulline	Urea cycle; arginine-, proline-, metabolism	Amino acid	-0.12	0.01
eicosapentaenoate (EPA; 20:5n3)	Essential fatty acid	Lipid	-0.12	-0.09
threitol	Nucleotide sugars, pentose metabolism	Carbohydrate	-0.13	0.01
glycine	Glycine, serine and threonine metabolism	Amino acid	-0.13	-0.10
p-cresol sulfate	Phenylalanine & tyrosine metabolism	Amino acid	-0.14	-0.13
phenylacetate	Phenylalanine & tyrosine metabolism	Amino acid	-0.14	-0.07
3-carboxy-4-methyl-5-propyl-2-furanpropanoate (CMPF)	Fatty acid, dicarboxylate	Lipid	-0.14	-0.14
naproxen	Drug	Xenobiotics	-0.14	-0.18
isobutyrylcarnitine	Valine, leucine and isoleucine metabolism	Amino acid	-0.15	-0.06
ascorbate (Vitamin C)	Ascorbate and aldarate metabolism	Cofactors and vitamins	-0.15	-0.10
hippurate	Benzoate metabolism	Xenobiotics	-0.16	-0.06
homostachydrine*	Food component, Plant	Xenobiotics	-0.17	0.01
hydroquinone sulfate	Drug	Xenobiotics	-0.17	-0.16

© 2021 Wei Y et al. *JAMA Network Open.*

phenylacetylglutamine	Phenylalanine & tyrosine metabolism	Amino acid	-0.18	-0.10
4-acetamidophenol	Drug	Xenobiotics	-0.20	0.03
3-(cystein-S-yl)acetaminophen*	Drug	Xenobiotics	-0.28	0.00
2-methoxyacetaminophen sulfate*	Drug	Xenobiotics	-0.29	0.02
p-acetamidophenylglucuronide	Drug	Xenobiotics	-0.35	-0.02
2-hydroxyacetaminophen sulfate*	Drug	Xenobiotics	-0.35	-0.01
4-acetaminophen sulfate	Drug	Xenobiotics	-0.35	-0.06

Note: Pearson's correlation coefficient r between pairs of metabolites measured in TwinsUK and KORA F4 cohort study.

© 2021 Wei Y et al. *JAMA Network Open.*

eTable 6. Sensitivity Analyses for Association Between Mannose and ARDS Risk Using Different Mendelian Randomization Methods and Different Sources of Association Results Between Genetic Instrumental Variables (SNPs) and ARDS Risk

Method	Source of association results between genetic instrumental variables (SNPs) and ARDS risk							
	All participants in MEARDS (N_{case}=403, $N_{control}$=1227)		All participants in iSPAAR cohort (N_{case}=983, $N_{control}$=1227)		All cases from ARDSNet and controls from MEARDS (N_{case}=580, $N_{control}$=1227)		All participants in MEARDS ALI excluded (N_{case}=352, $N_{control}$=1227)	
	OR (95%CI)	P	OR (95%CI)	P	OR (95%CI)	P	OR (95%CI)	P
GSMR	0.64 (0.53, 0.78)	$7.46×10^{-6}$	0.80 (0.68, 0.94)	$7.49×10^{-3}$	0.85 (0.71, 0.92)	$8.33×10^{-3}$	0.81 (0.69, 0.96)	$1.66×10^{-2}$
**Sensitivity analysis**								
IVW	0.70 (0.62, 0.78)	$2.79×10^{-9}$	0.82 (0.74, 0.91)	$1.57×10^{-4}$	0.82 (0.73, 0.92)	$6.35×10^{-4}$	0.84 (0.76, 0.93)	$1.20×10^{-3}$
MR-PRESSO	0.70 (0.63, 0.79)	$8.77×10^{-5}$	0.82 (0.76, 0.88)	$3.44×10^{-4}$	0.81 (0.74, 0.88)	$3.41×10^{-4}$	0.84 (0.77, 0.91)	$1.90×10^{-3}$
MR-Median	0.63 (0.51, 0.79)	$4.87×10^{-5}$	0.79 (0.66, 0.95)	0.01	0.81 (0.67, 0.99)	0.04	0.84 (0.70, 0.93)	0.04
MR-Maxlik	0.70 (0.62, 0.79)	$9.20×10^{-9}$	0.82 (0.74, 0.91)	$1.67×10^{-4}$	0.82 (0.73, 0.92)	$6.34×10^{-4}$	0.84 (0.76, 0.94)	$1.40×10^{-3}$
MR-ModeBase	0.61 (0.44, 0.83)	$1.96×10^{-3}$	0.74 (0.57, 0.96)	0.02	0.82 (0.64, 1.05)	0.01	0.78 (0.60, 0.98)	0.05
MR-RAPS (MLE)	0.70 (0.59, 0.83)	$4.58×10^{-5}$	0.82 (0.71, 0.94)	$5.84×10^{-3}$	0.81 (0.69, 0.95)	$8.68×10^{-3}$	0.84 (0.72, 0.97)	0.02
MR-RAPS (Shrinkage)	0.70 (0.59, 0.83)	$3.43×10^{-5}$	0.82 (0.71, 0.94)	$4.91×10^{-3}$	0.81 (0.69, 0.94)	$7.38×10^{-3}$	0.84 (0.72, 0.97)	0.02

* A series of comparable MR methods to verify the robustness of results including inverse-variance weighted (IVW), MR-PRESSO, weighted median (MR-Median), maximum likelihood (MR-Maxlik), mode-based estimation (MR-ModeBase), and Robust Adjusted Profile Score (MR-RAPS). Causal

© 2021 Wei Y et al. *JAMA Network Open.*

association between mannose and ARDS risk was inferred using different MR analytical methods for association between genetic variants and metabolites, including GSMR, IVW MR-PRESSO, MR-Median, MR-Maxlik, MR-ModeBase, MR-RAPS(MLE), and MR-RAPS (Shrinkage).

© 2021 Wei Y et al. *JAMA Network Open.*

eFigure 1. Standard Quality Control Procedure for GWAS Data

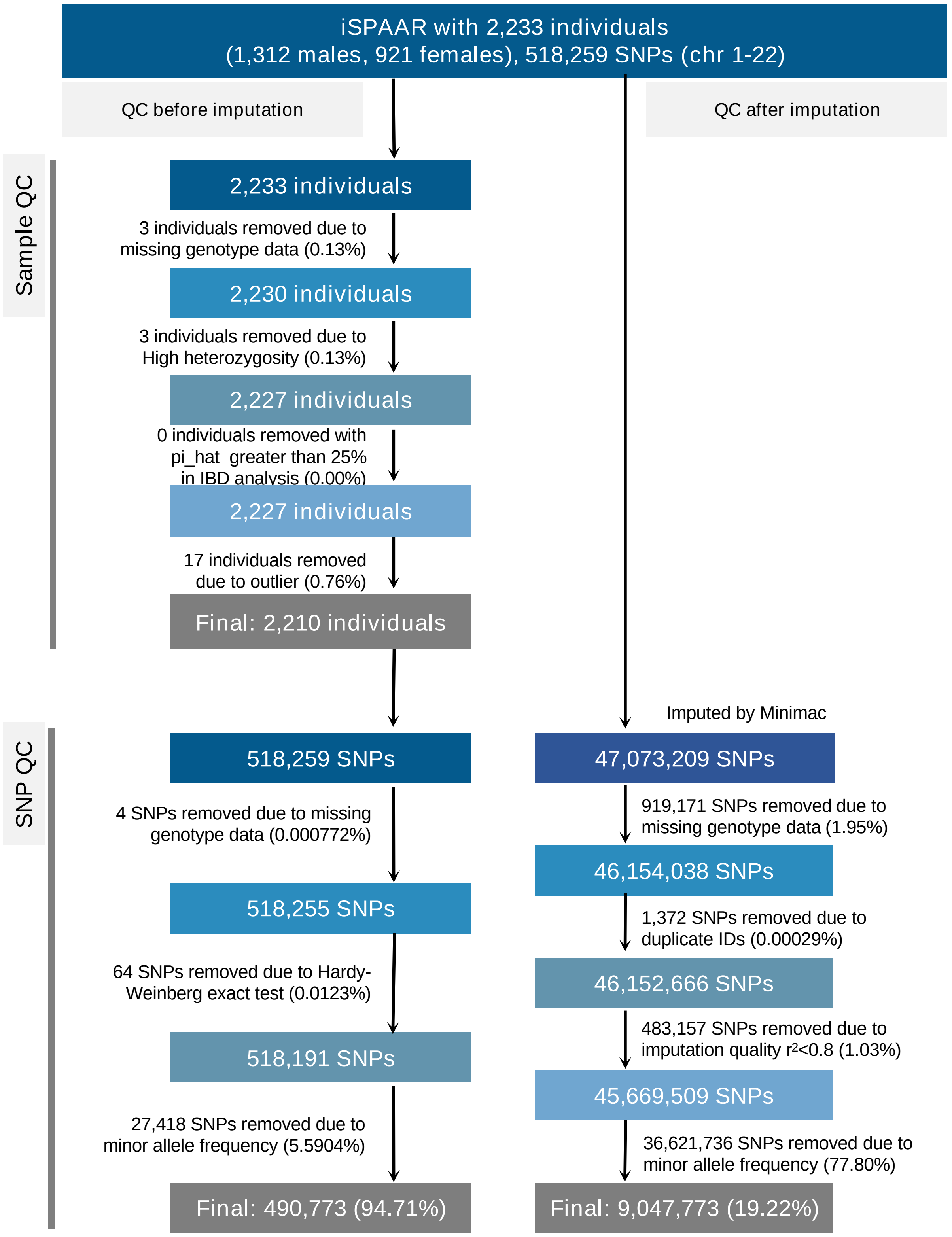

© 2021 Wei Y et al. *JAMA Network Open.*

eFigure 2. GSMR Analysis Workflow

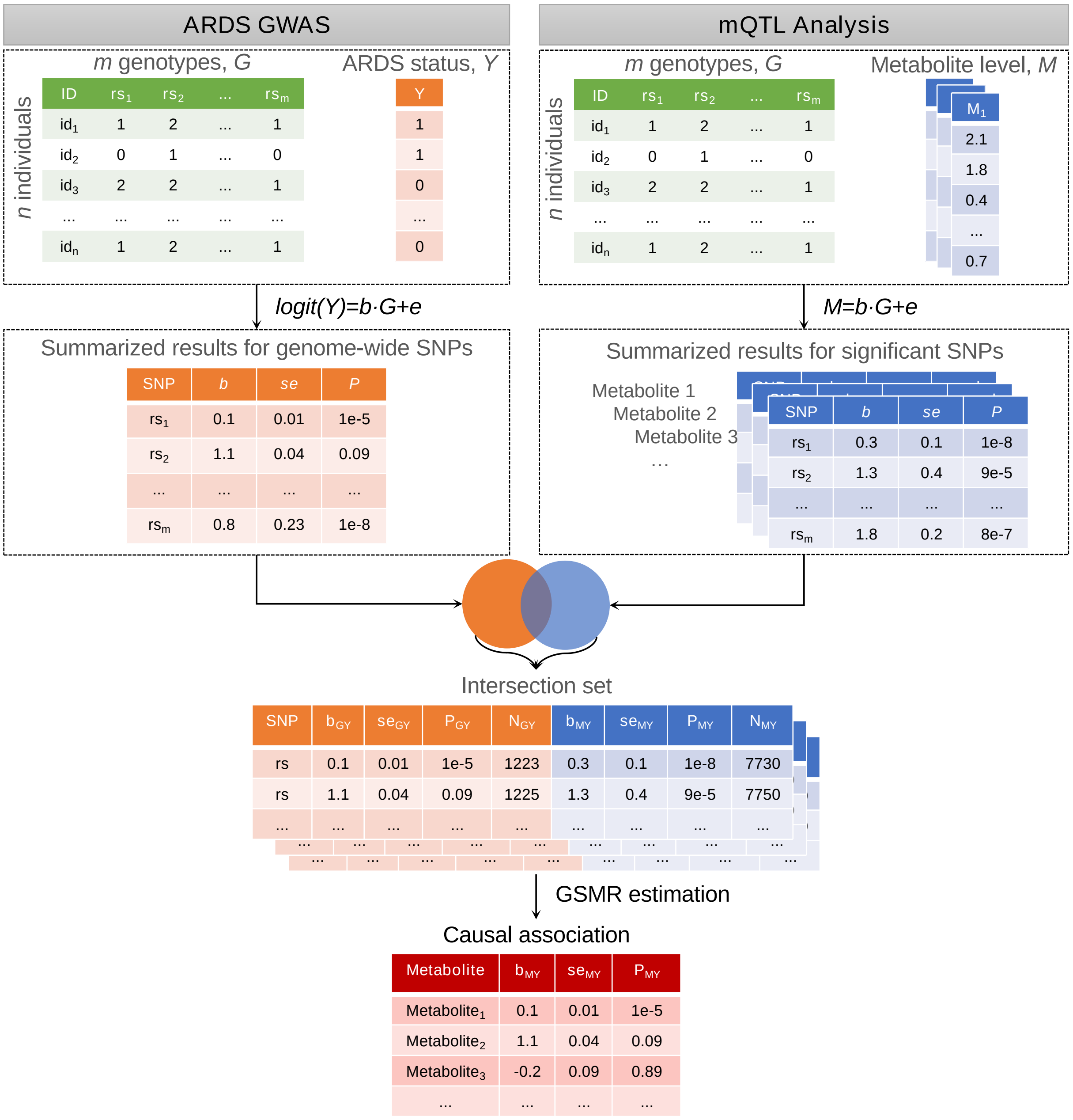

© 2021 Wei Y et al. *JAMA Network Open.*

eFigure 3. Framework Diagram of Mendelian Randomization

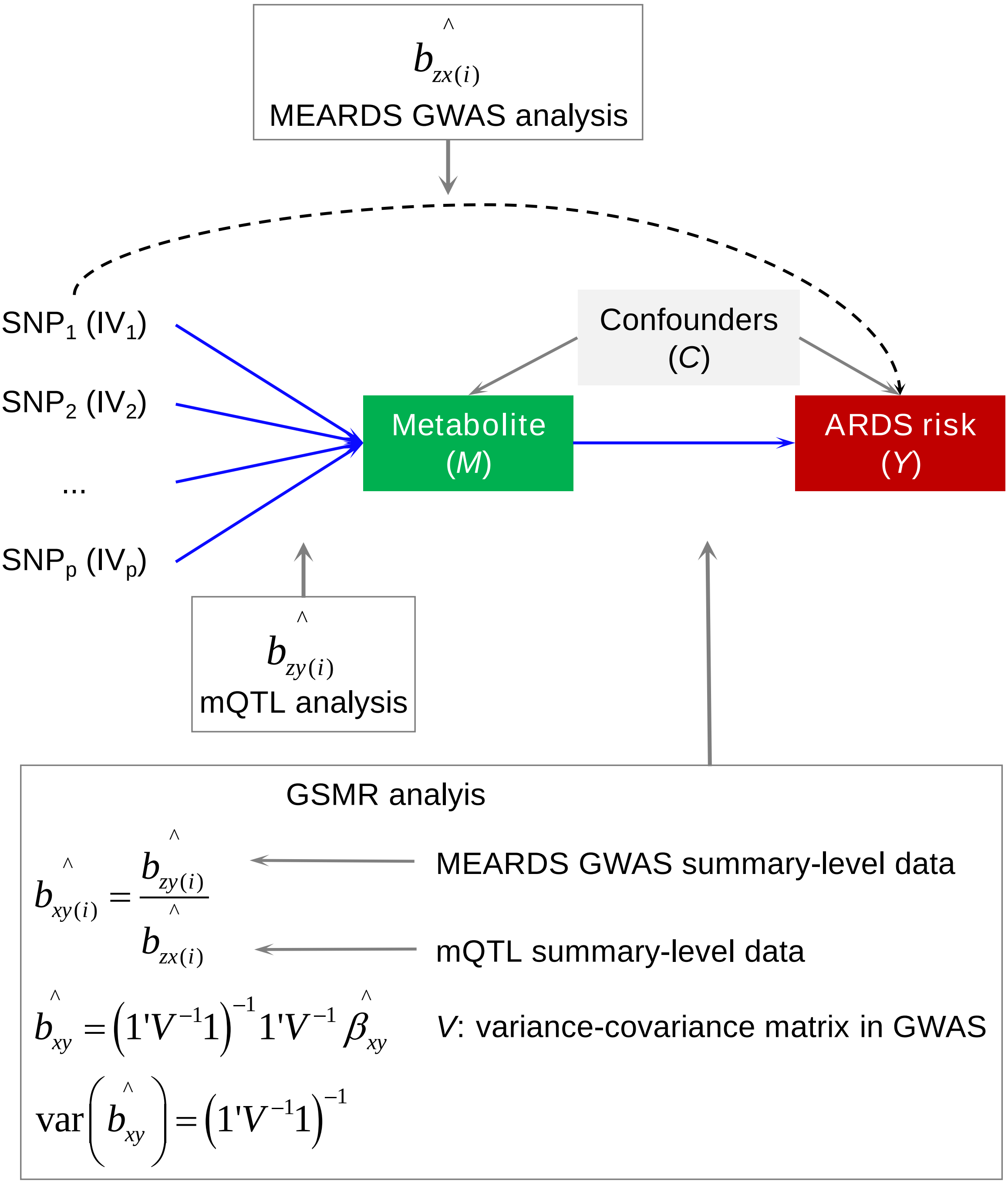

© 2021 Wei Y et al. *JAMA Network Open.*

eFigure 4. Sensitivity Analysis for Candidate Metabolites Associated With ARDS Risk

Causal associations between X-11440, X-12850, tryptophan betaine, and alpha-hydroxyisovalerate and ARDS risk were inferred after a series of comparable MR analytical methods, including a generalized summary data-based Mendelian randomization analysis (GSMR), inverse-variance weighting (IVW), MR-PRESSO, MR-Median, MR-Maxlik, MR-ModeBase, MR-RAPS (MLE), and MR-RAPS (Shrinkage).

The horizontal axis represents the effect size of the selected instrumental variable on metabolites (X-11440, X-12850, tryptophan betaine, and alpha-hydroxyisovalerate), and the vertical axis represents the effect size of the instrumental variable on ARDS risk. Each cross represents an instrumental variable and confidence intervals regarding x- and y- axis. The lines with different colors were estimated by different Mendelian Randomization methods; the slope of each line represents the estimated association effect between metabolites and ARDS risk of different Mendelian Randomization methods.

© 2021 Wei Y et al. *JAMA Network Open.*

A

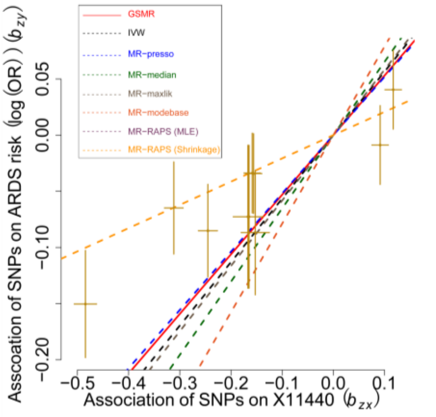

Method		OR(95%CI)	*P*
——	GSMR	1.70 (1.37, 2.11)	1.36×10^{-6}
Sensitivity analysis			
-----	IVW	1.76 (1.28, 2.42)	1.93×10^{-6}
-----	MR-PRESSO	1.68 (1.21, 2.34)	1.23×10^{-6}
-----	MR-Median	1.92 (1.37, 2.68)	9.62×10^{-5}
-----	MR-Maxlik	1.79 (1.30, 2.48)	2.17×10^{-6}
-----	MR-ModeBase	2.20 (1.37, 3.53)	3.37×10^{-4}
-----	MR-RAPS (MLE)	1.23 (1.19, 1.27)	2.73×10^{-8}
-----	MR-RAPS (Shrinkage)	1.23 (1.19, 1.27)	5.16×10^{-8}

B

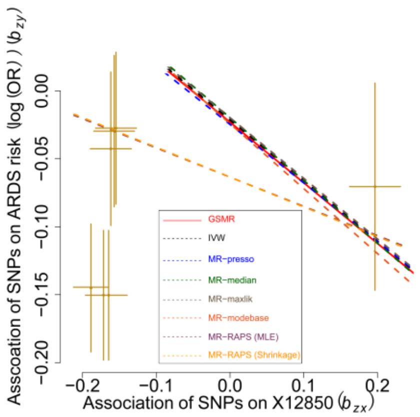

Method		OR(95%CI)	*P*
——	GSMR	0.64 (0.53, 0.78)	1.46×10^{-6}
Sensitivity analysis			
-----	IVW	0.64 (0.54, 0.77)	5.19×10^{-4}
-----	MR-PRESSO	0.65 (0.58, 0.73)	0.02
-----	MR-Median	0.63 (0.48, 0.83)	1.31×10^{-4}
-----	MR-Maxlik	0.64 (0.53, 0.78)	4.17×10^{-4}
-----	MR-ModeBase	0.62 (0.44, 0.86)	1.09×10^{-3}
-----	MR-RAPS (MLE)	0.95 (0.89, 1.01)	0.09
-----	MR-RAPS (Shrinkage)	0.95 (0.89, 1.01)	0.08

C

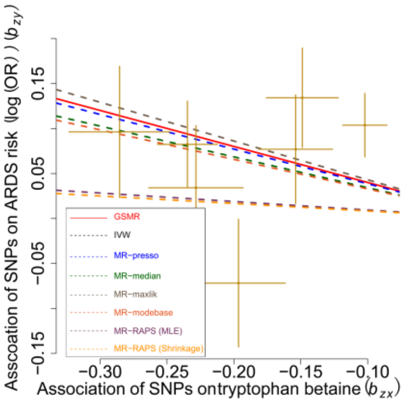

Method		OR(95%CI)	*P*
——	GSMR	0.67 (0.54, 0.83)	2.22×10^{-4}
Sensitivity analysis			
-----	IVW	0.65 (0.50, 0.86)	2.49×10^{-3}
-----	MR-PRESSO	0.68 (0.51, 0.91)	0.04
-----	MR-Median	0.71 (0.53, 0.95)	0.02
-----	MR-Maxlik	0.65 (0.49, 0.86)	2.42×10^{-3}
-----	MR-ModeBase	0.72 (0.51, 1.02)	0.06
-----	MR-RAPS (MLE)	0.91 (0.87, 0.95)	1.06×10^{-4}
-----	MR-RAPS (Shrinkage)	0.92 (0.88, 0.97)	2.50×10^{-3}

D

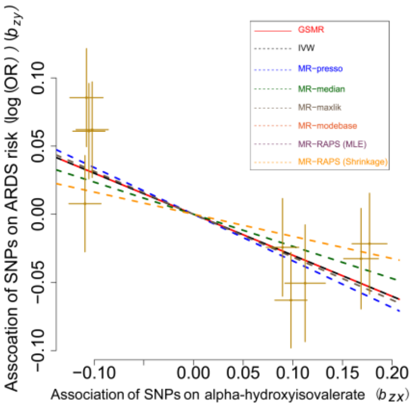

Method		OR(95%CI)	*P*
——	GSMR	0.74 (0.63, 0.87)	2.81×10^{-4}
Sensitivity analysis			
-----	IVW	0.74 (0.63, 0.86)	1.57×10^{-4}
-----	MR-PRESSO	0.71 (0.60, 0.84)	3.96×10^{-3}
-----	MR-Median	0.79 (0.61, 1.03)	0.08
-----	MR-Maxlik	0.73 (0.62, 0.86)	1.65×10^{-4}
-----	MR-ModeBase	0.85 (0.60, 1.20)	0.35
-----	MR-RAPS (MLE)	0.85 (0.81, 0.89)	5.61×10^{-12}
-----	MR-RAPS (Shrinkage)	0.85 (0.81, 0.89)	7.11×10^{-12}

© 2021 Wei Y et al. *JAMA Network Open.*

eFigure 5. Association Between Serum Mannose and Acute physiology severity using GSMR

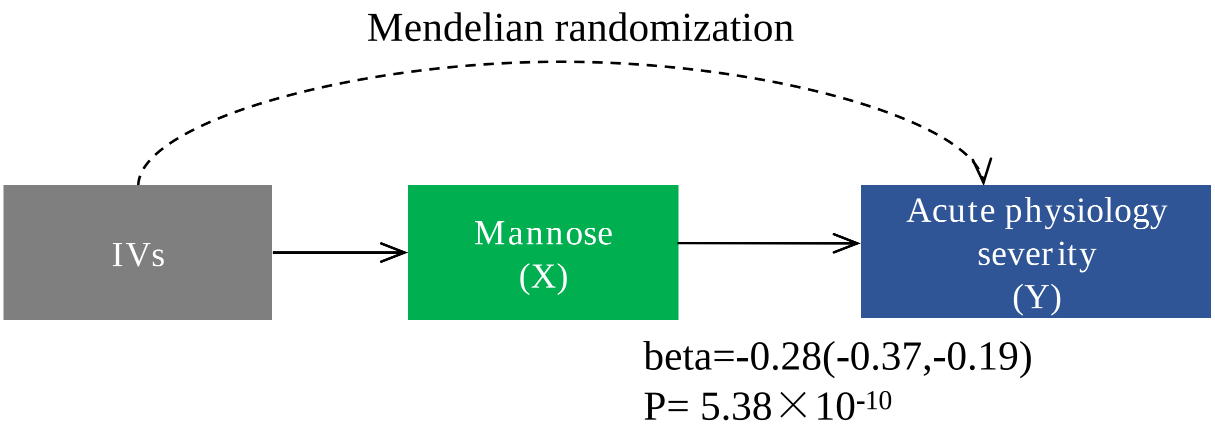

© 2021 Wei Y et al. *JAMA Network Open.*

eAppendix. Supplementary Methods

© 2021 Wei Y et al. *JAMA Network Open.*

1. GSMR Algorithm

Y is the outcome (risk of ARDS), *x* is the exposure (metabolite), and *z* is an instrumental variable. Four steps were performed in GSMR and presented in Supplementary Figure S5 and Supplementary Figure S3 (Nat Commun. Jan 15 2018;9(1):224):

i. The effect of each metabolite on ARDS ($\hat{b}_{xy}$) through: $\hat{b}_{xy(i)} = \hat{b}_{zy(i)}/\hat{b}_{zx(i)}$, $\hat{\beta}_{xy} = \{\hat{b}_{xy(1)}, \hat{b}_{xy(2)}, \cdots, \hat{b}_{xy(m)}\}$, where $\hat{b}_{zy(i)}$ is the estimate of effect of SNP on ARDS from MEARDS GWAS (Genome-Wide Association Study Of Acute Lung Injury Risk Loci In The Ispaar Consortium. 2011:A5536-A5536) and $\hat{b}_{zx(i)}$ is the estimate of a SNP effect on the abundance of a metabolite from mQTL (Nat Genet. Jun 2014;46(6):543-550).

ii. The sample variance of the effect of a metabolite on ARDS $\hat{b}_{xy}$ was estimated by $var\left(\hat{b}_{xy(i)}\right) = b_{xy(i)}^2\left[\frac{var\left(\hat{b}_{zx(i)}\right)}{b_{zx(i)}^2} + \frac{var\left(\hat{b}_{zy(i)}\right)}{b_{zy(i)}^2} - \frac{var^2\left(\hat{b}_{zx(i)}\right)}{b_{zx(i)}^4}\right]$, and the covariance of the effect calculated according to linkage disequilibrium (LD) correlation between the two SNPs (provided by MEARDS GWAS dataset).

iii. The estimated effect value of $\hat{b}_{xy}$ was calculated by the generalized least square method $\hat{b}_{xy} = (1'V^{-1}1)^{-1}1'V^{-1}\hat{\beta}_{xy}$.

iv. The approximate χ^2 test statistic was used to test the statistical significance of $\hat{b}_{xy}$: $T_{GSMR} = \hat{b}_{xy}^2/\,var\left(\hat{b}_{xy}\right)$.

© 2021 Wei Y et al. *JAMA Network Open.*

2. Metabolomics Testing Procedures and Platform in Metabolon Inc.

Sample Accessioning: Following receipt, samples were inventoried and immediately stored at -80°C. Each sample was accessioned into the Metabolon LIMS system and assigned a unique identifier associated with the original source identifier. This identifier was used to track all sample handling, tasks, results, etc. The samples (and all derived aliquots) were tracked by the LIMS system. All portions of any sample were automatically assigned their own unique identifiers by LIMS when a new task was created; the relationship of these samples was also tracked. All samples were maintained at -80°C until processing.

Sample Preparation: Samples were prepared using the automated MicroLab STAR® system from Hamilton Company. Several recovery standards were added before the first step in the extraction process for QC purposes. To remove protein and dissociate small molecules bound to protein or trapped in a precipitated protein matrix, proteins were precipitated with methanol under vigorous shaking for 2 min (Glen Mills GenoGrinder 2000) followed by centrifugation to recover chemically diverse metabolites. The resulting extract was divided into five fractions: two for analysis by two separate reverse-phase (RP)/UPLC-MS/MS methods with positive ion mode electrospray ionization (ESI), one for analysis by RP/UPLC-MS/MS with negative ion mode ESI, one for analysis by HILIC/UPLC-MS/MS with negative ion mode ESI, and one reserved for backup. Samples were placed briefly on a TurboVap® (Zymark) to remove organic solvent. Sample extracts were stored overnight under nitrogen before analysis.

QA/QC: Controls were analyzed in concert with experimental samples: a pooled matrix sample generated by a small volume of each experimental sample (or alternatively, a pool of well-characterized human plasma) served as a technical replicate throughout the data set; extracted water samples served as process blanks; and a cocktail of QC standards that were carefully chosen not to interfere with measurement of endogenous compounds was added to every analyzed sample, allowing instrument performance monitoring and chromatographic alignment. Instrument variability was determined by the median relative standard deviation (RSD) for standards added to each sample before injection into mass spectrometers. Overall process variability was determined by calculating median RSD for all endogenous metabolites (i.e., non-instrument

© 2021 Wei Y et al. *JAMA Network Open.*

standards) present in 100% of pooled matrix samples.

Ultrahigh Performance Liquid Chromatography-Tandem Mass Spectroscopy (UPLC-MS/MS): All methods used a Waters ACQUITY ultra-performance liquid chromatography (UPLC) and Thermo Scientific Q-Exactive high resolution/accurate mass spectrometer interfaced with a heated electrospray ionization (HESI-II) source and Orbitrap mass analyzer operated at 35,000 mass resolution. Sample extract was dried and reconstituted in solvents compatible with each of the four methods. Each reconstitution solvent contained a series of standards at fixed concentrations to ensure injection and chromatographic consistency. One aliquot was analyzed using acidic positive ion conditions and chromatographically optimized for more hydrophilic compounds. Extracts were gradient-eluted from a C18 column (Waters UPLC BEH C18-2.1x100 mm, 1.7 µm) using water and methanol containing 0.05% perfluoropentanoic acid (PFPA) and 0.1% formic acid (FA). Another aliquot was analyzed using acidic positive ion conditions; however, it was chromatographically optimized for more hydrophobic compounds. Extracts were gradient eluted from the C18 column using methanol, acetonitrile, water, 0.05% PFPA, and 0.01% FA and operated at an overall higher organic content. Another aliquot was analyzed using basic negative ion optimized conditions using a separate dedicated C18 column. Basic extracts were gradient eluted from the column using methanol and water with 6.5 mM ammonium bicarbonate at pH 8. The fourth aliquot was analyzed by negative ionization following elution from a HILIC column (Waters UPLC BEH Amide 2.1x150 mm, 1.7 µm) using a gradient consisting of water and acetonitrile with 10 mM ammonium formate, pH 10.8. The MS analysis alternated between MS and data-dependent MSn scans using dynamic exclusion. The scan range varied slightly between methods but covered 70–1000 m/z.

Bioinformatics: The informatics system consisted of four major components, the Laboratory Information Management System (LIMS), data extraction and peak-identification software, data processing tools for QC and compound identification, and a collection of information interpretation and visualization tools for use by data analysts. The hardware and software foundations for these informatics components were the LAN backbone and a database server running Oracle 10.2.0.1 Enterprise Edition.

LIMS: The purpose of the Metabolon LIMS system was to enable fully auditable

© 2021 Wei Y et al. *JAMA Network Open.*

laboratory automation through a secure, easy to use, and highly specialized system. The scope of the Metabolon LIMS system encompasses sample accessioning, sample preparation and instrumental analysis, and reporting and advanced data analysis. All subsequent software systems are grounded in LIMS data structures. It has been modified to leverage and interface with in-house information extraction and data visualization systems as well as third-party instrumentation and data analysis software.

Data Extraction and Compound Identification: Raw data were extracted, peak-identified, and QC-processed using Metabolon's hardware and software. These systems are built on a web-service platform using Microsoft's NET technologies, which runs on high-performance application servers and fiber-channel storage arrays in clusters to provide active failover and load-balancing. Compounds were compared to library entries of purified standards or recurrent unknown entities. Metabolon maintains a library based on authenticated standards that contains the retention time/index (RI), mass to charge ratio (m/z), and chromatographic data (including MS/MS spectral data) on all molecules present in the library. Further, biochemical identifications are based on three criteria: retention index within a narrow RI window of the proposed identification, accurate mass match to the library ±10 ppm, and MS/MS forward and reverse scores between experimental data and authentic standards. MS/MS scores are based on comparison of ions present in the experimental spectrum and ions present in the library spectrum. While there may be similarities between these molecules based on one of these factors, use of all three data points can be utilized to distinguish and differentiate biochemicals. More than 3,300 commercially available purified standard compounds have been acquired and registered in LIMS for analyses on all platforms for determination of their analytical characteristics. Additional mass spectral entries have been created for structurally unnamed biochemicals, which have been identified by virtue of their recurrent nature (both chromatographic and mass spectral). These compounds have the potential to be identified by future acquisition of a matching purified standard or by classical structural analysis.

Curation procedure: A variety of curation procedures were carried out to ensure that a high-quality dataset was available for statistical analyses and data interpretation. QC and curation processes were designed to ensure accurate and consistent identification of true chemical entities and to remove those representing system artifacts, mis-

© 2021 Wei Y et al. *JAMA Network Open.*

assignments, and background noise. Metabolon data analysts use proprietary visualization and interpretation software to confirm the consistency of peak identification among samples. Library matches for each compound were checked for each sample and corrected if necessary.

Metabolite Quantification and Data Normalization: Peaks were quantified using area-under-the-curve. For studies spanning multiple days, a data normalization step was performed to correct variation resulting from instrument inter-day tuning differences. Essentially, each compound was corrected in run-day blocks by registering the medians to equal one (1.00) and normalizing each data point proportionately (termed “block correction”). For studies that did not require more than one day of analysis, no normalization was necessary, other than for data visualization. In certain instances, biochemical data may have been normalized to an additional factor (e.g., cell counts, total protein as determined by Bradford assay, osmolality) to account for differences in metabolite levels due to differences in amount of material present in each sample.

© 2021 Wei Y et al. *JAMA Network Open.*
